# Resource-Mediated Indirect Effects of Grassland Management on Arthropod Diversity

**DOI:** 10.1371/journal.pone.0107033

**Published:** 2014-09-04

**Authors:** Nadja K. Simons, Martin M. Gossner, Thomas M. Lewinsohn, Steffen Boch, Markus Lange, Jörg Müller, Esther Pašalić, Stephanie A. Socher, Manfred Türke, Markus Fischer, Wolfgang W. Weisser

**Affiliations:** 1 Terrestrial Ecology Research Group, Department of Ecology and Ecosystem Management, School of Life Sciences Weihenstephan, Technische Universität München, Freising, Germany; 2 Department of Animal Biology, Institute of Biology, University of Campinas, Campinas, Sao Paulo, Brazil; 3 Institute of Plant Sciences, University of Bern, Bern, Switzerland; 4 Max-Planck-Institute for Biogeochemistry, Jena, Germany; 5 Institute of Biochemistry and Biology, University of Potsdam, Potsdam, Germany; 6 Biodiversity and Climate Research Centre, Senckenberg Gesellschaft für Naturforschung, Frankfurt/Main, Germany; WSL Institute for Snow and Avalanche Research SLF, Switzerland

## Abstract

Intensive land use is a driving force for biodiversity decline in many ecosystems. In semi-natural grasslands, land-use activities such as mowing, grazing and fertilization affect the diversity of plants and arthropods, but the combined effects of different drivers and the chain of effects are largely unknown. In this study we used structural equation modelling to analyse how the arthropod communities in managed grasslands respond to land use and whether these responses are mediated through changes in resource diversity or resource quantity (biomass). Plants were considered resources for herbivores which themselves were considered resources for predators. Plant and arthropod (herbivores and predators) communities were sampled on 141 meadows, pastures and mown pastures within three regions in Germany in 2008 and 2009. Increasing land-use intensity generally increased plant biomass and decreased plant diversity, mainly through increasing fertilization. Herbivore diversity decreased together with plant diversity but showed no response to changes in plant biomass. Hence, land-use effects on herbivore diversity were mediated through resource diversity rather than quantity. Land-use effects on predator diversity were mediated by both herbivore diversity (resource diversity) and herbivore quantity (herbivore biomass), but indirect effects through resource quantity were stronger. Our findings highlight the importance of assessing both direct and indirect effects of land-use intensity and mode on different trophic levels. In addition to the overall effects, there were subtle differences between the different regions, pointing to the importance of regional land-use specificities. Our study underlines the commonly observed strong effect of grassland land use on biodiversity. It also highlights that mechanistic approaches help us to understand how different land-use modes affect biodiversity.

## Introduction

Negative effects of intensive grassland land use on biodiversity have been found for many taxa including plants [1], herbivorous and carnivorous arthropods [2]–[4], pollinating insects [5] and birds [6]. Despite the growing consensus that intensive land use has generally negative effects on biodiversity [7], the particular mechanisms that lead to a decrease in biodiversity are often not fully understood [8], because land use consists of various modes that can have opposing or additive effects on biodiversity. In semi-natural grasslands important land-use modes are mowing, grazing and fertilization. Several observational and experimental studies found decreasing diversities of plants and arthropods with increasing frequency of mowing events e.g. [4], [9]–[11], with increasing fertilization intensity e.g. [12], [13] or with increasing grazing intensity [14]–[16]. Whereas effects of land-use modes on plants are often direct, e.g. when mowing hinders seed set of late-flowering plants, effects on higher trophic levels such as insect herbivores or carnivores may be either direct or mediated by changes in the plant community.

Several hypotheses have been proposed to describe the effects of differently diverse plant communities on the diversity and abundance of herbivores and predators. The ‘Resource Heterogeneity Hypothesis’ (RHH) predicts that more diverse resources provide more niches for a greater number of specialized species at higher trophic levels [17]–[20], i.e. herbivore diversity is promoted by increased plant diversity and predator diversity increases in response to herbivore diversity – a positive trophic cascade. In contrast to the RHH, the ‘More Individuals Hypothesis’ (MIH) proposes that diversity of consumers increases when resource quantity increases, i.e. plant biomass for herbivores and herbivore biomass for predators [21]. According to the MIH, this positive effect of resource abundance (or biomass) on consumer diversity is mediated by an increase in total consumer abundance. Borer et al. [22] studied plant diversity effects on arthropod diversity in experimental plant communities and found that more diverse plant communities hosted more diverse herbivore communities, but that this effect was mediated by higher overall plant and herbivore biomass. While the RHH assumes a direct link between resource and consumer diversity, the MIH assumes an indirect link through resource and consumer abundance, i.e. resource abundance increases consumer abundance which in turn increases consumer diversity. Thus, arthropod biomass and diversity may be differently affected by increasing land-use intensity depending on the mechanistic relationships between land use and the herbivore and carnivore communities.

We combined detailed information on grassland land-use modes (fertilization, grazing and mowing) with biomass and species richness data of plants and arthropods to analyse effects of land-use intensity on the arthropod community. Although experimental short-term manipulation of land use can elucidate immediate effects of the land-use modes (such as mowing), it is not clear whether these mechanisms operate similarly when different land-use practices are combined in agricultural grasslands and under long term conditions. This can only be assessed by studying grasslands which have been used as meadows or pastures continuously for several years or decades. In our study, we build on the work of Socher et al. [1] who tested for direct and indirect effects of grassland land use on plant diversity and biomass. We studied effects of land use on the arthropod community including both direct and indirect (via the plant community) chains of effects. The study system includes grasslands which have been managed for at least 20 years and comprise a wide range of land-use intensities. By including three different regions in Germany and by sampling in two consecutive years, we were able to consider the generality of observed patterns.

**Figure 1:**
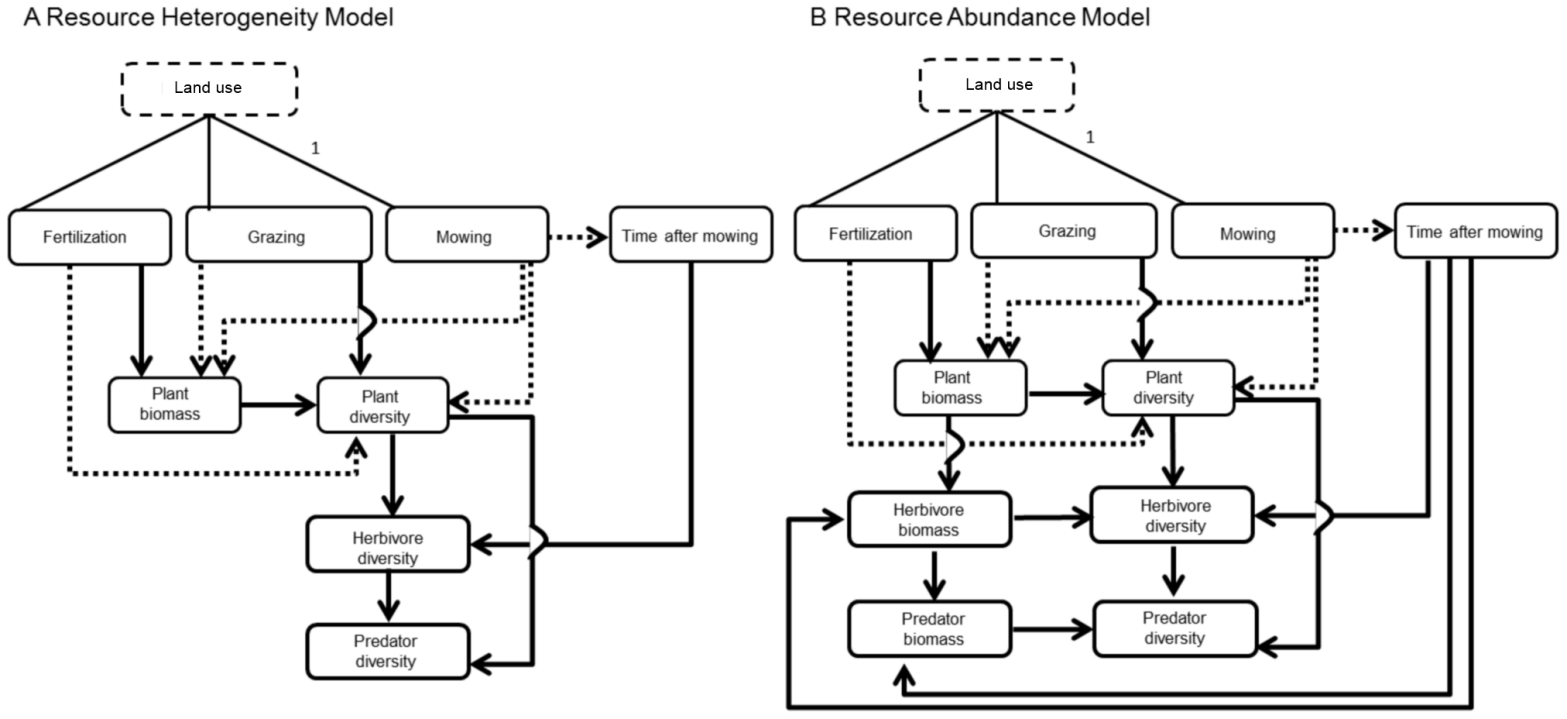
Concepts for structural equation models. ‘Land use’ is a latent (i.e. unmeasured) variable influencing the management components. The path coefficient for the correlation between ‘Land use’ and cutting frequency was a priori set to 1. The arrows indicate expected causal effects between measured variables; solid arrows represent expected positive effects and dotted arrows expected negative effects. For more detailed information on model definition and variable calculation see the Method section and Appendix S1.

Based on the two hypotheses mentioned above and previous knowledge on mechanisms of grassland land use, we defined two alternative models. In the first model (‘Resource Heterogeneity Model’) we tested whether land-use intensity affects herbivore and predator diversity via changes in the diversity of their respective resources (RHH). According to the RHH we expected positive effects of plant diversity on herbivore diversity and of herbivore diversity on predator diversity (Figure 1 A). In the second model (‘Resource Abundance Model’), we tested whether effects of land-use intensity affect arthropods through changes in resource quantity (MIH) and added herbivore and predator biomass to the model (Figure 1 B). According to the MIH, we expected plant biomass to have a positive effect on herbivore diversity through herbivore biomass and positive effects of herbivore biomass on predator diversity, respectively.

Within both models (Figure 1 A & B), we expected mowing to decrease plant diversity (by a loss of disturbance-sensitive species) and to decrease plant biomass (through mechanical disturbances during the growing period). Grazing was expected to increase plant diversity (increasing number of niches for plants or preventing competitive exclusion) and decrease plant biomass (recurrent disturbance of plant growth). Another possible plant species response to grazing or mowing is overcompensating for tissue loss leading to the opposite expectation (i.e. increase of plant biomass following grazing or mowing). However, overcompensation has usually been demonstrated for particular species, not at the community level e.g. [23], [24], where a decrease of plant biomass is more likely. Fertilization was expected to decrease plant diversity (dominance of fast-growing species) and increase plant biomass (increased nutrient input). Based on the findings of Socher et al. [1] we expected plant biomass to be negatively correlated with plant diversity.

In our study we asked the following questions: 1) How do land-use modes, singly or in combination, affect arthropod diversity? 2) How are effects of land-use intensity on herbivore diversity mediated by the responses of plants to land use (do they follow the ‘Resource Heterogeneity Hypothesis’ or the ‘More Individuals Hypothesis’)? 3) Are the responses of predators to land use governed by the same mechanisms as responses of herbivores?

## Materials and Methods

### Study sites and land use

The Biodiversity Exploratory research program (www.biodiversity-exploratories.de) was established in 2006 within three regions in Germany: (1) Schorfheide-Chorin (SCH) in north-east Germany (3–140 m a.s.l., 53°02′ N 13°83′ E, annual mean precipitation 500–600 mm, mean temperature 8–8.5°C). (2) Hainich-Dün (HAI) in central Germany (285–550 m a.s.l., 51°20′ N 10°41′ E, 500–800 mm, 6.5–8°C). (3) Swabian Alb (ALB) in south-west Germany (460–860 m a.s.l., 48°43′ N 9°37′ E, 700–1000 mm, 6–7°C). Within each region, 50 plots of 50 m × 50 m size serve as basis for surveys of biodiversity or ecosystem processes. Those plots were chosen from a total of 500 candidate plots in each region on which initial vegetation and land-use surveys were conducted. The 50 plots per region cover the whole regional gradient of land-use modes and intensity [25].

Land-use modes on the studied grasslands include mowing with different frequency (meadows), grazing by different kinds of livestock (cattle and sheep pastures) or both mowing and grazing (mown pastures). Grasslands are either unfertilized or fertilized with different amounts of fertilizer. During the study years all grassland plots continued to be managed by farmers in the same way as the surrounding grasslands. Grassland land use was assessed each year (since 2006) by standardized interviews with farmers and land-owners. From these interviews, we derived information on fertilization, grazing and mowing: Fertilization intensity was calculated as the total amount of nitrogen applied per hectare and year, in the form of organic or chemical fertilizer. For grazing, information on livestock units and the duration of grazing periods were combined as a measure of grazing intensity. Mowing intensity was calculated as the number of mowing events per year.

For our analyses we used land-use information from the two years prior to sampling and the sampling year. Mowing intensity included mowing events in the sampling year only up to the sampling event (e.g. for samples taken in June, mowing events later than June in the sampling year were not included). As mowing not only has long-term but also short-term effects on arthropods (e.g. Humbert et al. [26] showed that mowing with machinery leads to high mortality in Orthoptera), we included the number of days between the arthropod sampling day and the last mowing event prior to the respective sampling as an additional land-use variable (‘Time after mowing’). As two arthropod samplings were conducted per year (see below), we used the mean number of days for each year. For a detailed description of land-use intensity calculations and an overview of land-use information see Appendix S1 as well as Table S1 & Table S2.

### Plant and arthropod sampling and plot selection

Plant diversity and plant biomass on the plots were assessed between mid-May and mid-June in 2008 & 2009 using vegetation surveys and aboveground biomass harvests following the methods described by Socher et al. [1]: On a 4 m × 4 m subplot in each plot all vascular plant species were identified in the field following the nomenclature of Wisskirchen & Häupler [27], categorized into functional groups (grasses, herbs, legumes) and their cover (%) was estimated. As vegetation was generally relatively uniform across the plot, we consider our subplot sample as representative for the 50 m × 50 m plot. Plant biomass was clipped at a height of 2 to 3 cm above ground and dried for 48 hours at 80°C before weighing. Occasionally occurring shrubs and litter were excluded before drying. In 2008, biomass samples were taken in two 25 cm × 50 cm subplots next to the vegetation survey plot. In 2009, biomass was harvested on eight subplots of 50 cm × 50 cm size adjacent to one side of the vegetation survey plot to better reflect small-scale variability. In both years, total plant biomass [g/m^2^] was recorded as the mean from the subplots. In mown and grazed plots, the subplots were fenced until biomass harvest to obtain productivity estimates unaffected by current year grazing or mowing. For the analyses we used plant species richness per plot as measure for plant diversity.

We sampled arthropods by sweep-netting with a total of 60 double-sweeps (one double-sweep is defined as moving the net from the left to the right and back perpendicular to the walking direction) along three plot borders (150 m in total) in June and August 2008 & 2009 during periods of dry weather conditions. The two sampling months were chosen because they cover the main activity period of arthropods and were found to represent the variation in diversity among plots equally well as sampling in five months (tested with a subset of plots in 2009, data not shown). We chose sweep-netting over suction sampling because of logistic difficulties with suction sampling on our large number of plots and because suction sampling was found to underrepresent Heteroptera (Gossner et al., unpublished data from our plots and [28]).

Sampling was conducted within seven days or fewer per region and within two weeks in all regions. Arthropods were preserved in 70% ethanol, sorted by taxonomic group and identified to species level by taxonomic experts. We focused on Araneae, Hemiptera (Cicadomorpha, Fulgoromorpha and Heteroptera), Coleoptera and Orthoptera because of their numerical dominance in the studied grasslands. Only adult individuals were considered for the analysis, because identification of juveniles is often difficult. Samples from both months within one year were pooled for analysis. Because sampling effort was standardized there was no need to adjust for differences in the number of sampled individuals. Accordingly, we used species richness as diversity measure.

Due to external influences, such as aggressive cows preventing us from entering several plots, the number of plots with a complete dataset (plant and arthropod data) differed somewhat between years: in 2008 we considered 124 plots and in 2009 we considered 141 plots (see Table 1 for the number of plots per region). Of those plots, 117 plots were considered in both years.

**Table 1:** Mean ± standard error for the sampled variables within each of the three regions.

	Vascular plants		Herbivores			Predators		
	No. species	Biomass (g)	No. species	Abundance	Biomass (g)	No. species	Abundance	Biomass (g)
**2008**								
Swabian Alb N = 43	33.3±10.3	598.1±302.9	33.3±10.6	628.6±570.9	15.3±14.3	5.4±3.5	10.6±8.3	0.4±0.4
Hainich-Dün N = 38	25.9±9.6	592.6±228.8	31.4±12.3	307.4±205.0	9.6±8.6	5.6±2.8	15.8±10.0	0.5±0.5
Schorfheide-Chorin N = 43	16.0±3.7	752.0±281.7	26.7±7.7	281.1±145.0	8.8±4.9	5.1±2.7	13.2±9.5	0.3±0.3
F-value_2,121_	46.72	4.56	4.64	11.89	5.09	0.29	3.25	0.91
*P*	<0.001	0.012	0.011	<0.001	0.008	0.752	0.042	0.404
Means over all regions	25.0±2.2	648.0±11.0	30.0±1.9	410.0±19.6	11.2±3.1	5.0±1.3	13.0±2.6	0.4±0.02
**2009**								
Swabian Alb N = 47	32.1±11.2	272.1±123.1	20.5±5.6	180.9±144.5	6.7±4.8	3.8±2.0	4.6±2.8	0.3±0.3
Hainich-Dün N = 48	33.6±12.1	299.7±128.7	16.6±7.1	73.0±93.4	2.8±4.4	4.3±2.5	8.5±7.2	0.2±0.2
Schorfheide-Chorin N = 46	19.8±4.2	331.2±130.5	22.1±8.0	163.2±105.1	4.7±4.0	5.2±2.9	9.8±7.2	0.3±0.6
F-value_2,140_	27.25	2.49	7.648	11.76	9.25	4.1	9.0	0.5
*P*	<0.001	0.086	<0.001	<0.001	<0.001	0.019	<0.001	0.61
Means over all regions	29.0±2.2	301.0±7	20.0±1.6	139.0±10.6	4.7±2.2	4.0±1.2	8.0±2.3	0.3±0.7

N = number of plots sampled in the respective year. Arthropod biomass was estimated from the species' mean length (see text). Means were tested for differences between regions with ANOVA. Degrees of freedom are indicated with the F-value_df Region, df Residuals_. Abundances are expresses as number of adult individuals whereas biomass is given in grams. Species lists of herbivores and predators can be found in Appendix S5 and Appendix S6, original datasets are available from the Dryad Digital Repository: http://doi.org/10.5061/dryad.f3b77.

### Ethics Statement

Field work permits were issued by the responsible state environmental offices of Baden-Württemberg, Thüringen and Brandenburg (according to § 72 BbgNatSchG, i.e. nature conservation law of Brandenburg). Out of the 150 initially sampled plots, 17 are part of grassland areas that are protected under the Habitats Directive (FFH), 58 are protected as LSG (landscape conservation site), 17 are protected as NSG (nature conservation site) and 5 are situated within a former military training area. Except the 5 plots which are situated in the former military training area and 6 plots that are situated on state property, all other grasslands are privately owned by the farmers. One individual of *Phytoecia cylindrica* (Coleoptera, Linnaeus, 1758), which is protected under BArtSchV (Federal Protection of Species Order) was sampled in 2008.

### Classification of trophic guilds and arthropod biomass assessment

All identified arthropod species were assigned to one of three trophic guilds (herbivores, predators and decomposers) based on their known main food resource as adults (Appendix S2). Because decomposers made up less than 2% of individuals in 90% of our samples, they were not included in the analysis. Arthropod biomass per plot was estimated by applying the general power function developed by Rogers et al. [29] to each sampled species: 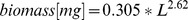



Where L is the mean length (in mm) of a species, derived from the same sources as given for the identification of feeding guilds (Appendix S2). The contribution of a species to total biomass was then calculated as: species-specific biomass × abundance of the species. Biomass for each plot was calculated for herbivores and predators separately by summing over all species. We used biomass rather than abundances as measure for resource abundance to be consistent with plant assessments.

### Statistical analysis

To compare means of the response variables among regions, we used ANOVA (aov) with ‘Region’ as explanatory variable in R [30].

For structural equation modelling, there are, in principle three main approaches [31]: 1) Strictly confirmatory, where a single model is defined and tested against the data. 2) Alternative modelling, where similar models are tested and compared by model selection for best fit. 3) Model generating, where one tentative initial model is simplified until the model fit cannot be improved. We used a combination of approaches 1 and 3 by defining two models based on the ‘Resource Heterogeneity’ (RHH) and ‘More Individuals’ (MIH) hypotheses (confirmatory approach, Figure 1) but using step-wise deletion of paths within these models when the model structure did not fit the data (model generating). The second step was included to assess whether the discrepancies between model and data structure were due to erroneous assumptions about interactions among variables (in this case model fit would be improved by step-wise deletion) or due to missing (i.e. not measured) variables (in that case model fit would not be improved).

Grassland land use was included in the models by the variables fertilization, grazing and mowing as well as ‘Time after mowing’ (as described in the first section of Materials and Methods). In addition, we added a latent (i.e. unmeasured) variable ‘Land use’ to describe the combined effect of land use. This latent variable accounts for correlations between the different land-use modes. As latent variables have no underlying data which the model algorithm can use to calculate its variance, either the latent's variance or one regression weight between the latent and one of the connected variables has to be fixed to 1. We chose to fix the regression weight between the latent variable and mowing frequency because mowing is a good descriptor of land-use intensity and it is correlated with both fertilization and grazing intensity [32]. The two other regression weights (between ‘Land use’ and grazing or fertilization) and the latent's variance can then be estimated by the model algorithm. To test for a non-linear effect of grazing intensity, we tried a quadratic transformation of grazing intensity, but because it did not improve model fit we kept the linear relationship. As the correlations between the land-use modes varied between regions, the model sometimes did not converge; in these cases, we included correlations between mowing and fertilization and between mowing and grazing instead of the latent variable. Except for the additional variables and pathways that were added in the ‘Resource Abundance Model’, all pathways were expected to be identical in both models (Fig. 1 A & B), as motivated in the Introduction.

We used the software package ‘sem’ in R for structural equation modelling [30] which fits models using Maximum Likelihood based on observed and expected covariance matrices. The model fit is estimated as the overall model p-value which indicates if the two covariance matrices are significantly different from each other (p<0.05, bad model fit) or not (p>0.05, good model fit [31], p.128f). A step-wise selection procedure was applied by hand using the corrected Akaike's Information Criterion (AICc) for models without appropriate fit (model p-value <0.05). If a second measure of model quality, the Goodness-of-fit (GoF) index, was above 0.75 for the final model, models without adequate fit were still used for comparison among regions.

To calculate the total effect of ‘Land use’ on the plant and arthropod variables, the standardized path coefficients within each possible pathway between ‘Land use’ and the respective variable were multiplied and all resulting products were summed (e.g. coefficients from ‘Land use’ to fertilization and from fertilization to plant biomass were multiplied and added to the products of pathways including grazing and mowing). Total effects among plant and arthropod variables were calculated accordingly. Data was transformed as necessary (further details in Appendix S3).

## Results

Over all plots we recorded 271 vascular plant species in 2008 and 281 vascular plant species in 2009; 54,660 herbivore individuals from 392 species were sampled in 2008 and 19,507 herbivores from 335 species in 2009; 1,823 predator individuals from 162 species were sampled in 2008 and 1,075 predators from 154 species in 2009. The average plant biomass per plot was 648 g/m^2^ in 2008 and 301 g/m^2^ in 2009, mean estimated herbivore biomass per plot was 11.2 g and 4.7 g (2008 and 2009 respectively) and mean estimated predator biomass per plot was 0.4 g and 0.3 g (including 1 and 5 plots with no predators in 2008 and 2009 respectively). The lower plant and herbivore biomass in 2009 compared to 2008 can be attributed to a shorter time-span between the onset of the vegetation period and sampling (as calculated for ‘Time after mowing’, see Appendix S1). Increasing plant biomass was correlated with a decrease in plant species diversity (see below) and with an increase in relative cover of grass species (compared to herbaceous species) (R^2^_adj_ = 0.269 in 2008 and R^2^_adj_ = 0.081 in 2009, *P*<0.001 in both years and all regions; Spearman's correlation).

In 2008 and 2009, plant and herbivore species richness as well as herbivore abundance and herbivore biomass differed significantly between regions and were highest in ALB (Table 1). Plant biomass also differed significantly between regions in 2008 and was highest in SCH while in 2009 it was similar among regions (Table 1). Predator species richness differed between regions only in 2009 (being highest in SCH) and was very similar among regions in 2008. Predator abundances differed between regions both in 2008 and 2009 and were lowest in ALB. Predator biomass did not differ between regions in both years (Table 1).

### Resource Heterogeneity Model

**Figure 2:**
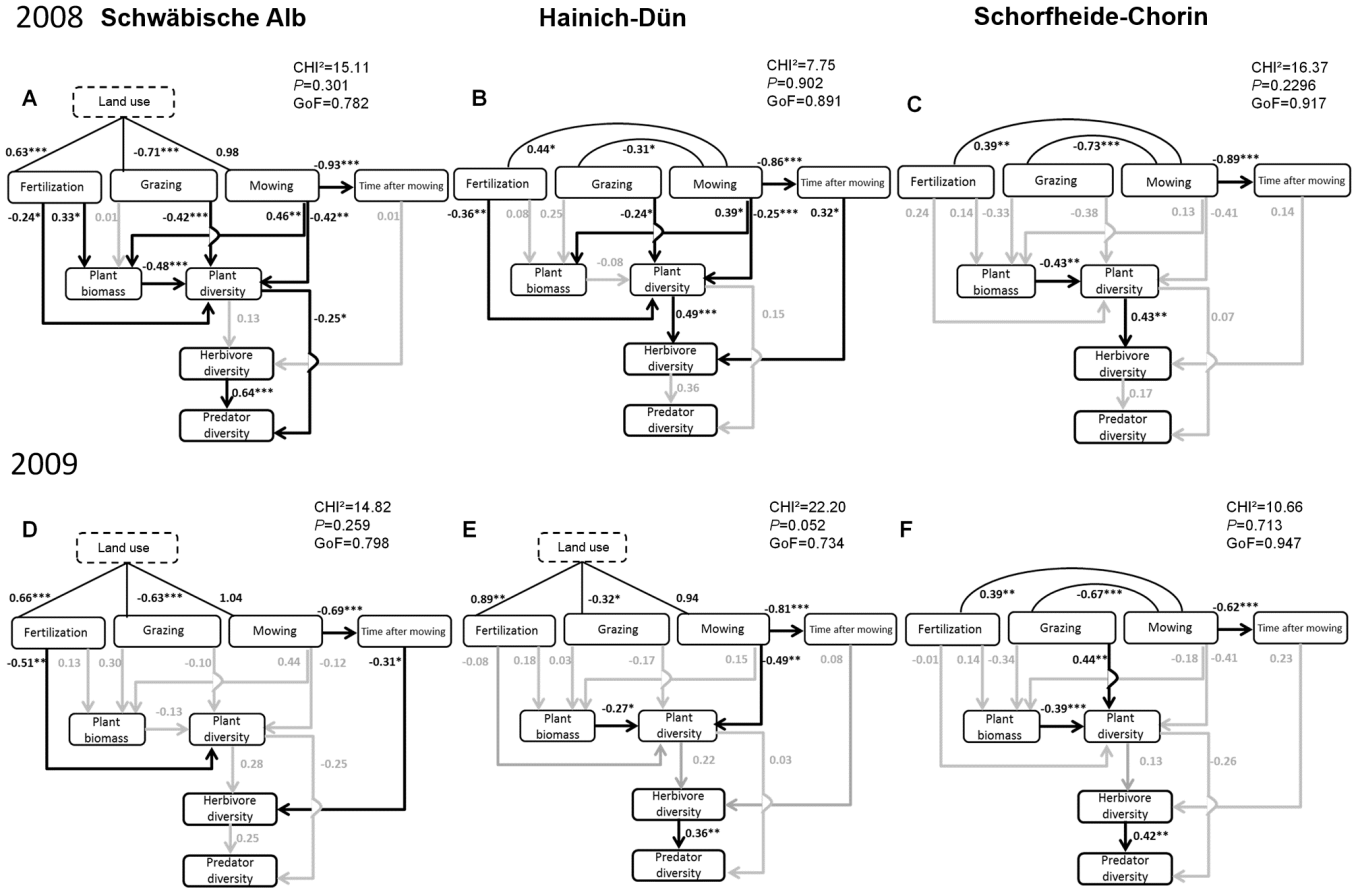
Standardized regression weights and significance levels from the Resource Heterogeneity Model. Models are shown for 2008 (A, B, C) and 2009 (D, E, F) for the Swabian Alb (A, D), Hainich-Dün (B, E) and Schorfheide-Chorin (C, F). Black solid lines and numbers indicate significant paths, grey arrows and numbers indicate non-significant paths. Correlation graphs for plant and arthropod measures can be found in Appendix S4. GoF =  Goodness of fit. Significance levels: P<0.05: */P<0.01: **/P<0.001: ***. Total effects in Tables 2 & 3 were calculated using the standardized regression weights, e.g. plant diversity effects on predator diversity in Swabian Alb 2008 were calculated as 0.13 (coefficient from plant diversity to herbivore diversity)*0.64 (herbivore diversity to predator diversity) *−0.25 (plant diversity to predator diversity)  = −0.17.

The Resource Heterogeneity Model fitted the 2008 and 2009 data well for each region (Figure 2). Except for the omission of the latent variable ‘Land use’ in three cases, no further path-selection procedure was applied. Despite this omission, correlations among the land-use modes were consistent between years and regions, where fertilization was positively correlated with ‘Land use’ (or mowing) and grazing was negatively correlated with ‘Land use’ (or mowing).

Fertilization generally had the predicted negative effect on plant diversity and a positive effect on plant biomass, although effects were significant only in ALB (for diversity in 2008 & 2009 and for biomass only in 2008) and HAI (for diversity in 2008). We found positive but not significant effects of grazing intensity on plant biomass (Figure 2) but grazing intensity consistently reduced plant diversity (except for a significant increase in SCH in 2009). Mowing frequency consistently increased plant biomass and decreased plant diversity (although only significant in ALB and HAI and mostly in 2008). Differences in effect direction were found in SCH (positive effect of fertilization on plant diversity, and negative effects of grazing and mowing on plant biomass) but all of those effects were not significant.

As expected, plant biomass was always negatively related to plant diversity even though the paths for HAI 2008 and ALB 2009 were not significant. Effects of plant diversity on herbivore diversity were positive in all regions and years, as predicted by the RHH (although only significant in HAI and SCH). Similarly, we found a positive effect of herbivore diversity on predator diversity in all regions and years (here it was significant in 3 of 6 cases).

When summing the direct and indirect effects of ‘Land use’ on the target variables, we found total effects to be positive for plant biomass and negative for plant diversity (Table 2). The only exception was SCH, due to the strong influence of grazing (negative on plant biomass in 2008 and positive on plant diversity in 2009). The total effect of ‘Land use’ on herbivore diversity was negative in all regions and both years, whereas total effects on predator diversity were generally very weak (<0.1). The total effects of plant diversity on herbivore diversity were all positive as they are identical to the direct effects in this model. Total effects of plant diversity on predator diversity were, however, generally weak. Overall, ‘Land use’ decreased plant diversity directly and indirectly through an increase in plant biomass, which led to a decrease in herbivore diversity and a following decrease in predator diversity.

**Table 2:** Standardized total effects derived from the Resource Heterogeneity Model for both years.

		Land use (mode) effects on				Plant diversity effects on	
		Plant biomass	Plant diversity	Herbivore diversity	Predator diversity	Herbivore diversity	Predator diversity
Swabian Alb	2008	0.65	−0.59	−0.09	0.09	0.13	−0.17
	2009	0.35	−0.44	0.10	0.03	0.28	0.06
Hainich-Dün	2008					0.49	0.32
Fertilization	2008	0.08	−0.37	−0.18	−0.06		
Grazing	2008	0.25	−0.26	−0.13	−0.05		
Mowing	2008	0.39	−0.56	−0.54	−0.19		
	2009	0.28	−0.56	−0.19	−0.08	0.22	0.11
Schorfheide-Chorin	2008					0.43	0.15
Fertilization	2008	0.41	0.18	0.10	0.01		
Grazing	2008	−0.33	−0.24	−0.10	0.0		
Mowing	2008	0.17	0.01	−0.01	0.0		
	2009					0.13	−0.20
Fertilization	2009	0.14	−0.06	0.0	0.02		
Grazing	2009	−0.03	0.46	0.06	0.02		
Mowing	2009	−0.19	−0.10	−0.16	−0.09		

Total effects were calculated by multiplying the standardized path coefficients on the single pathways between two variables and summing up those values for all possible pathways. Standardized total effects can range between −1 and 1. Effects are shown from the first row on the second row. For a description on how total effects were calculated see last paragraph in Material and Methods and refer to legend of Figure 2 for an example. Original datasets are available from the Dryad Digital Repository: http://doi.org/10.5061/dryad.f3b77.

### Resource Abundance Model

**Figure 3:**
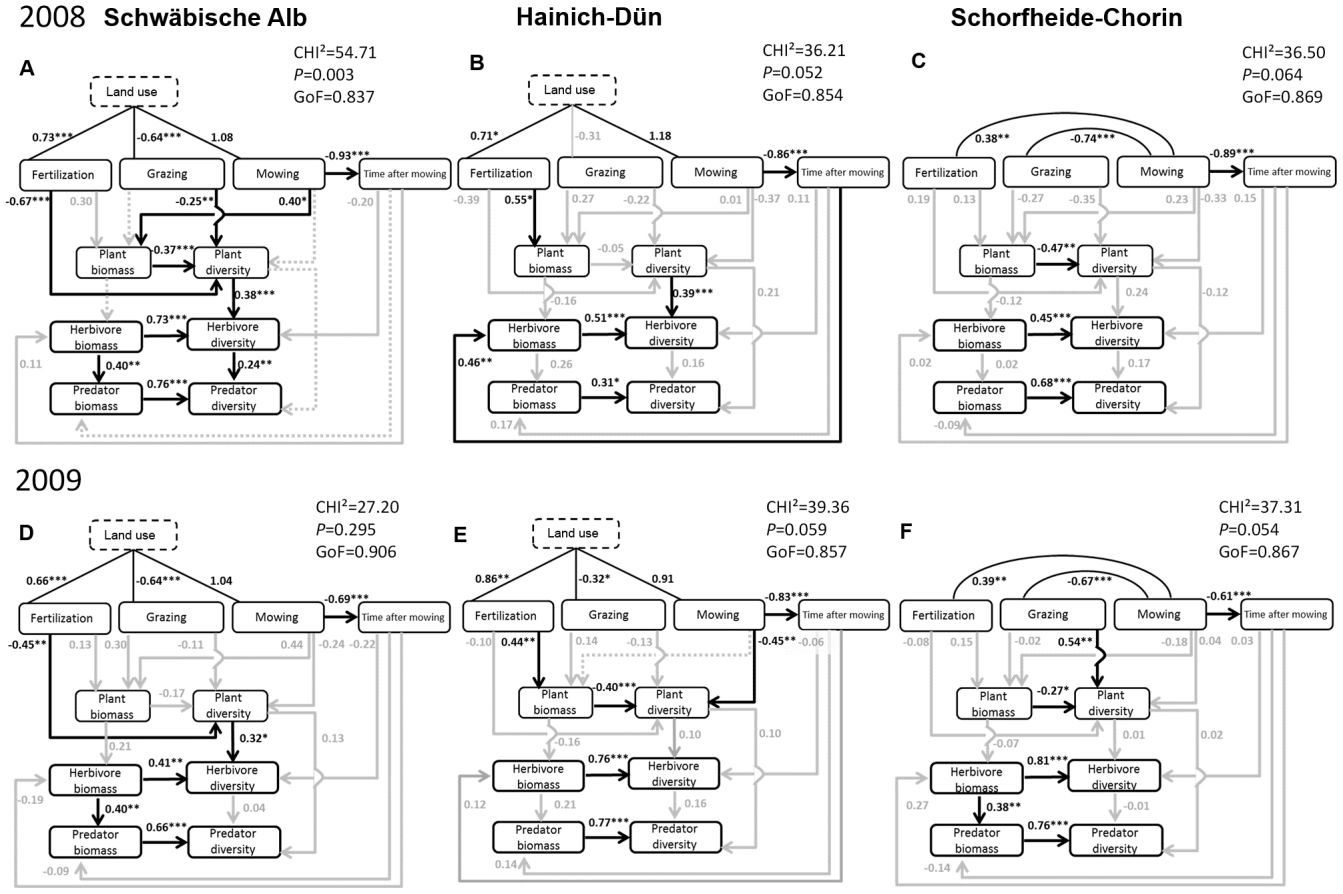
Standardized regression weights and significance levels from the Resource Abundance Model. Models are shown for 2008 (A, B, C) and 2009 (D, E, F) for the Swabian Alb (A, D), Hainich-Dün (B, E) and Schorfheide-Chorin (C, F). Black solid lines and numbers indicate significant paths, grey arrows and numbers indicate non-significant paths. Grey dotted paths were excluded during the step-wise selection procedure. Correlation graphs for plant and arthropod measures can be found in Appendix S4. GoF =  Goodness of fit. Significance levels: *P*<0.05: */*P*<0.01: **/*P*<0.001: ***.

The Resource Abundance Model fitted the 2008 data adequately well for HAI and SCH, but not for ALB, even after applying path-selection procedures. For 2009, we found adequate model fits for all three regions after applying path-selection to HAI (Figure 3). Compared with the Resource Heterogeneity Model, the relationships among land-use modes remained the same, except that the latent variable could be kept in the model for HAI in 2008 (because the model converged in the first run). The direct effects of the land-use modes on plant diversity and biomass changed slightly in magnitude but not in direction compared with the first model and between years. The change in magnitude is due to the fact that effects were standardized (i.e. values are relative to each other) which is influenced by the change in number of variables included in the model. As in the first model, plant biomass was negatively related to plant diversity (although not significant in HAI 2008) and plant diversity had a positive effect on herbivore diversity (although only significant in ALB & HAI 2008 and SCH 2009; Figure 3).

We did not find a significant direct effect of plant biomass on herbivore biomass as predicted by the MIH in any region or year. As the relationship between plant and herbivore biomass may be weakened if there are shifts in the relative abundances of differently sized herbivore species, we also tested the Resource Abundance Model using arthropod abundances instead of biomass but again did not find any effect of plant biomass on herbivore abundances, neither in 2008 or 2009 (Figure S1: Standardized regression weights and significance levels from the resource abundance model including arthropod abundances). Herbivore biomass had a significant positive effect on herbivore diversity in all regions and both years. Herbivore biomass also had a positive effect on predator biomass, significant in three out of six cases (ALB 2008, 2009, SCH 2009). Predator biomass itself had a significant positive effect on predator diversity in all regions and both years.

Total effects of ‘Land use’ on plant and arthropod diversity as well as on plant biomass were identical in direction and similar in effect strength to the Resource Heterogeneity Model for both years, including the reverse effects in SCH due to grazing. Total effects of plant diversity on herbivore diversity were positive, but total effects of plant biomass on herbivore diversity were absent or very weak. Total effects of herbivore biomass on predator diversity were positive (Table 3).

**Table 3:** Standardized total effects derived from the Resource Abundance Model for both years.

		Land use (mode) effects on				Plant diversity effects on		Plant biomass effects on	Herbivore biomass effects on
		Plant biomass	Plant diversity	Herbivore diversity	Predator diversity	Herbivore diversity	Predator diversity	Herbivore diversity	Predator diversity
Swabian Alb	2008	0.65	−0.56	−0.09	−0.06	0.38	0.09	−0.14	0.48
	2009	0.05	−0.48	0.07	0.02	0.32	0.15	0.04	0.28
Hainich-Dün	2008	0.84	−0.71	−0.70	−0.36	0.39	0.27	−0.10	0.16
	2009	−0.37	−0.43	−0.53	−0.38	0.77	0.52	0.03	0.66
Schorfheide-Chorin	2008					0.24	−0.08	0.17	0.09
Fertilization	2008	−0.28	0.32	0.09	−0.02				
Grazing	2008	0.23	−0.46	−0.12	0.03				
Mowing	2008	0.13	−0.39	−0.25	0.06				
	2009					0.01	0.03	−0.06	0.28
Fertilization	2009	−0.02	−0.08	0.0	0.0				
Grazing	2009	−0.18	0.59	0.01	0.02				
Mowing	2009	0.15	0.0	0.01	0.02				

Total effects were calculated by multiplying the standardized path coefficients on the single pathways between two variables and summing up those values for all possible pathways. Standardized total effects can range between −1 and 1. Effects are shown from the first row on second row. For an example on how effects were calculated see legend of Figure 2.

In summary, ‘Land use’ increased plant biomass, but this effect was not leading to an increase in herbivore biomass. Nevertheless, increasing herbivore biomass generally increased predator biomass which in turn increased predator diversity.

## Discussion

We tested whether effects of land-use intensity on the diversity of arthropod herbivores and predators are mediated by changes in the diversity or biomass of their respective resources, i.e. plants and herbivores. Although the strength of effects varied between regions and years, we found negative effects of land-use intensity on the diversity of both arthropod groups which were mediated by different pathways. For herbivores, the results were consistent with the ‘Resource Heterogeneity Hypothesis’, as we found significant effects of plant diversity on herbivore diversity with no evidence for effects mediated by plant biomass. For predators, results were more consistent with the ‘More Individuals Hypothesis’, i.e. predator diversity and biomass were more strongly affected indirectly by changes in herbivore biomass than by direct effects of herbivore diversity.

### Total land-use effects on arthropods and differences between regions

The negative total effect of land-use intensity on herbivore and predator diversity is consistent with previous reports on the consequences of grassland land use on arthropods [2]–[4], but the effects of the individual land-use modes (especially grazing) differed between regions and years and were only partly consistent with expectations. For instance, moderate grazing was found to have a positive effect on arthropod diversity in several studies [33] but we found only weak total effects of grazing intensity on arthropod diversity (<0.2). A wider range of grazing intensities in our study system compared to other studies might explain the absence of a positive effect in our case; e.g. Dennis et al. [34] found higher abundance and diversity of arthropods under moderate grazing intensity of sheep compared to high grazing intensity. The different types of livestock in our study system likely changed effects of grazing as well because herbivore diversity in 2008 was significantly lower on cattle-grazed plots compared to sheep-grazed plots and mixed grazing by cattle and horses had a significant positive effect on predator diversity compared with cattle or sheep grazing (Figure S2: Effects of livestock type on plant and insect species richness in 2008). Differences in the grazing gradients between regions and changes in grazing practices between years can also explain the change from a negative effect of grazing to a positive effect of grazing on plant diversity in SCH.

The different correlations among the land-use modes in the different regions are, in fact, a striking result of our study. Whereas all modes were significantly correlated with the latent variable ‘Land use’ in ALB, fertilization and grazing were sometimes (HAI) or always (SCH) independent of each other in the other regions. The discrepancies were probably caused by the range of land-use options realized in the different regions. In ALB, where most of the grasslands are managed by small farming enterprises or farming families, we find extensively grazed, unfertilized plots, e.g. sheep pastures on nutrient-poor hillsides, as well as intensively grazed, fertilized plots. Thus, grazing and fertilization are closely linked in this region. In the other two regions, plots with low grazing intensity sometimes receive low fertilizer input (for instance, in organic farming practices) but in other cases they are highly fertilized mown pastures that are only grazed at the end of the plant growth period. This weakened the correlation between grazing and fertilization.

### Effects of land-use intensity on plant biomass and diversity

Socher et al. [1] extensively discussed the effects of fertilization, grazing and mowing on plant biomass and diversity using the same plots as the present study, thus here we only summarize the main points: Increasing fertilization intensity generally decreased plant diversity and increased biomass as found in many preceding studies. Negative effects of high mowing frequency on plant diversity support previous findings from different types of grasslands and various regions [35]. The negative effect of grazing on plant diversity appears to contrast the general finding that grazing increases plant diversity [36]. However, most previous studies compared grazed with ungrazed sites and thus found that grazing increased plant diversity via increased sward heterogeneity [11], [37]. In our case, grazing ranged from no grazing to very high grazing intensities, which could result in a non-linear or hump-shaped effect of grazing on plant diversity. As including a non-linear effect of grazing did not improve our model (see Method section) it seems likely that the negative effect of very high grazing intensities is exceeding the positive effect moderate grazing has compared to non-grazed sites.

### Effects of changes in the plant community on herbivores

Our results showed clear evidence that land-use effects on herbivore diversity are mediated by plant diversity as predicted by the ‘Resource Heterogeneity Hypothesis’. The fact that we did not find effects on herbivore diversity mediated by plant biomass is in contrast to findings from experimental plant communities [22], which showed that arthropod diversity was only indirectly affected by plant diversity through increased plant biomass. This indirect effect on arthropod diversity was additionally mediated by arthropod biomass (measured as biovolume) and therefore followed the ‘More Individuals Hypothesis’[22]. The differences between results from our study in managed grasslands and results from experimental plant communities may be due to the absence of correlations between the proportion of particular plant functional groups and plant diversity in biodiversity experiment, as both variables are manipulated similarly. In contrast, the grasslands in our study system showed an increasing cover proportion of grasses with decreasing plant diversity. As found by Haddad et al. [38] the presence of grass species (which are productive but have low nutritional quality for herbivores) led to a decrease in total insect abundance by 25% even though total plant biomass increased. Only when grass species were absent and all plants were of higher nutritional quality, insect abundance was best explained by plant biomass. Hence, the higher cover of grass species on grasslands with low plant diversity in our study system led to a higher plant biomass but at the same time could not sustain higher herbivore biomass possibly because the overall nutritional value for the arthropods did not increase together with plant biomass.

### Effects of changes in the herbivore community on predators

We found direct and indirect effects between herbivore and predator diversity, indicating mechanisms in accordance with both the ‘Resource Heterogeneity’ and the ‘More Individuals Hypothesis’. One example would be the ALB 2008 where both direct and indirect effects were significant and strong. This indicates a complementary role of both mechanisms which might be the result of different predator groups reacting to either one of the mechanisms. In our study, the total effect of herbivore biomass on predator diversity was stronger in five out of six cases than the direct effect of herbivore diversity (compare Figure 3 and Table 3). This agrees with results from a plant diversity experiment, where effects of plants on herbivores were consistent with the ‘Resource Heterogeneity Hypothesis’ but effects of herbivores on predators were more in agreement with the ‘More Individuals Hypothesis’ [12]. Further research is needed to understand how predator diversity is affected by land use, as herbivore biomass was not affected by any of the included factors. This is relevant for sustainable land use of grasslands in agricultural dominated landscapes, because high predator abundance and diversity enhances biocontrol potential e.g. [39].

### Where to go from here

We tested the effect of land-use intensity on important grassland herb-dwelling arthropods over a wide geographic range. To achieve standardized sampling in the vegetation layer across a large number of plots, sweep-net sampling was used which is a suitable method to representatively sample important herbivores (e.g. Heteroptera) as well as predators (e.g. Araneae) among arthropods (e.g. [40]–[42]). Nevertheless, it is a less well-performing method to sample other functional groups such as pollinators (butterflies and bees) [40] or ground-dwelling species [43]. Additional methods (such as suction sampling, pitfall traps or pan traps) might therefore be advised if a study's focus is not only on herb-dwelling species. Disentangling the differences which might apply to different functional groups within herbivores and predators (e.g. sucking vs. chewing herbivores or predators with different hunting strategies) will further increase our understanding of land-use effects. One promising approach was recently proposed by Lavorel et al. [44] who included producer and consumer traits in structural equation models to understand how land use affects ecosystem services through changes in the trait composition of the groups which provide the services.

### Conclusions

Our results emphasize the importance of studying indirect effects of land-use intensity on the arthropod community, as they showed that herbivores and predators respond to changes of different aspects of their resources. We confirm that herbivore diversity is responding positively to higher plant diversity in grasslands, whereas herbivore biomass matters more than diversity for how predator diversity is affected by land use. By including different regions we showed on the one hand that the negative effects of high fertilization intensity and high mowing frequency on arthropod diversity are consistent over large scales; but on the other hand the variability of land-use traditions clearly indicates that findings cannot be easily extended to a wider geographical context. Our results thus not only emphasize the importance of land use for biodiversity changes, but also the need for more differentiated approaches to disentangle how different land-use modes have different effects on biodiversity, and how chains of effects differ for different aspects of biodiversity.

## Supporting Information

Figure S1 **Standardized regression weights and significance levels from the resource abundance model including arthropod abundances.** Models are shown after step-wise deletion of non-significant paths. Black solid lines and numbers indicate significant paths; grey arrows indicate non-significant paths. Grey, dotted paths were excluded during the step-wise selection procedure. Significance level: p<0.05: */p<0.01: **/p<0.001: ***.
(DOCX)

Figure S2 **Effects of livestock type on plant and insect species richness in 2008.** Means per plot and standard errors are shown. Horizontal lines indicate significant differences based on Tukey's HSD test. Significance levels: p<0.05: */p<0.01: **/p<0.001: ***.
(DOCX)

Table S1 **Mean and range of land-use activities in the three regions and during the years considered for the analysis with samplings from 2008.**
(DOC)

Table S2 **Mean and range of land-use activities in the three regions and during the years considered for the analysis with samplings from 2009.**
(DOC)

Appendix S1 **Assessment and calculation of land-use information.**
(DOC)

Appendix S2 **Classification of trophic guilds.**
(DOC)

Appendix S3 **Structural equation model setup and path-selection procedure.**
(DOC)

Appendix S4 **Bivariate correlations between model variables.**
(DOC)

Appendix S5 **List of arthropod species sampled in 2008**
(PDF)

Appendix S6 **List of arthropod species sampled in 2009.**
(PDF)
