# Supplementary material for: Resource-Mediated Indirect Effects of Grassland Management on Arthropod Diversity
Source: PLoS One. 2014 Sep 4;9(9):e107033. doi: 10.1371/journal.pone.0107033 (PMC4154770; doi:10.1371/journal.pone.0107033)
Supplement: Figure S1 — Standardized regression weights and significance levels from the resource abundance model including arthropod abundances. Models are shown after step-wise deletion of non-significant paths. Black solid lines and numbers indicate significant paths; grey arrows indicate non-significant paths. Grey, dotted paths were excluded during the step-wise selection procedure. Significance level: p<0.05: */p<0.01: **/p<0.001: ***. (DOCX) [file pone.0107033.s001.docx]

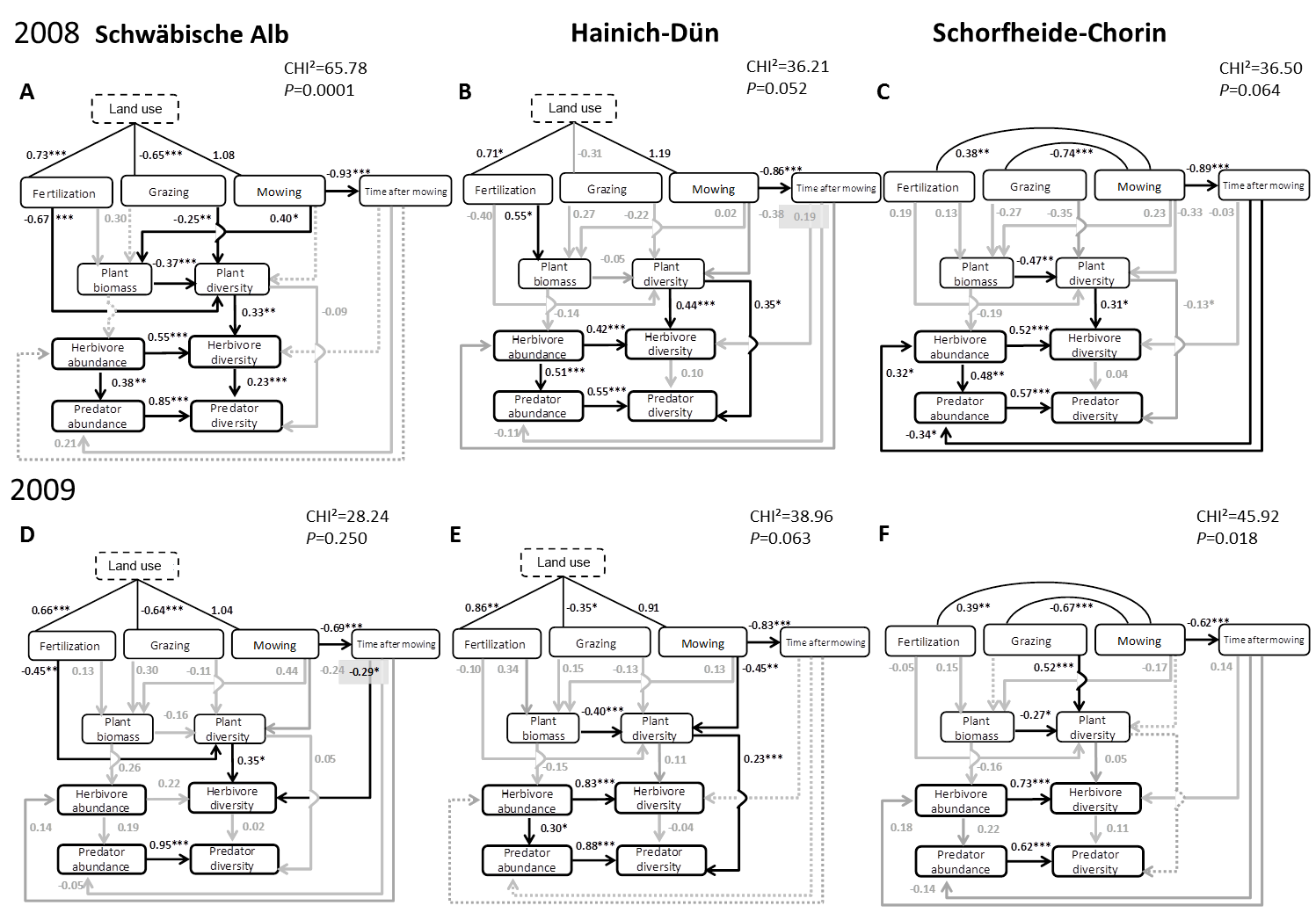


Figure S1: Standardized regression weights and significance levels from the resource abundance model including arthropod abundances. Models are shown after step-wise deletion of non-significant paths. Black solid lines and numbers indicate significant paths; grey arrows indicate non-significant paths. Grey, dotted paths were excluded during the step-wise selection procedure. Significance level: p<0.05: * / p<0.01: ** / p<0.001: ***.
