# Supplementary material for: Resource-Mediated Indirect Effects of Grassland Management on Arthropod Diversity
Source: PLoS One. 2014 Sep 4;9(9):e107033. doi: 10.1371/journal.pone.0107033 (PMC4154770; doi:10.1371/journal.pone.0107033)
Supplement: Figure S2 — Effects of livestock type on plant and insect species richness in 2008. Means per plot and standard errors are shown. Horizontal lines indicate significant differences based on Tukey's HSD test. Significance levels: p<0.05: */p<0.01: **/p<0.001: ***. (DOCX) [file pone.0107033.s002.docx]

Appendix D: Effects of livestock type

Figure S2: Effects of livestock type on plant and insect species richness in 2008. Means per plot and standard errors are shown. Horizontal lines indicate significant differences based on Tukey’s HSD test. Significance levels: p<0.05: * / p<0.01: ** / p<0.001: ***.
