# Supplementary material for: Resource-Mediated Indirect Effects of Grassland Management on Arthropod Diversity
Source: PLoS One. 2014 Sep 4;9(9):e107033. doi: 10.1371/journal.pone.0107033 (PMC4154770; doi:10.1371/journal.pone.0107033)
Supplement: Table S2 — Mean and range of land-use activities in the three regions and during the years considered for the analysis with samplings from 2009. (DOC) [file pone.0107033.s004.doc]

**Table S1: Mean and range of land-use activities in the three regions and during the years considered for the analysis with samplings from 2009.**

|  |  | **Schwäbische Alb** | | | **Hainich-Dün** | | | **Schorfheide-Chorin** | | |
| --- | --- | --- | --- | --- | --- | --- | --- | --- | --- | --- |
|  |  | 2007 | 2008 | 2009 | 2007 | 2008 | 2009 | 2007 | 2008 | 2009 |
| **Fertilization**  **[kg N/ha]** | Number of plots | 26.0 | 26.0 | 28.0 | 22.0 | 21.0 | 20.0 | 10.0 | 11.0 | 8.0 |
|  | Mean intensity | 53.0 | 52.9 | 68.3 | 114.6 | 66.4 | 90.9 | 75.9 | 87.4 | 82.6 |
|  | Max intensity | 148.0 | 148.0 | 444.0 | 1314.4 | 140.0 | 162.0 | 125.0 | 126.0 | 115.0 |
| **Grazing [livestock unit days/ha]** | Number of plots | 26.0 | 26.0 | 24.0 | 41.0 | 45.0 | 30.0 | 26.0 | 22.0 | 25.0 |
|  | Mean intensity | 245.3 | 246.3 | 2.6 | 125.8 | 127.0 | 2.3 | 221.4 | 331.6 | 1.7 |
|  | Max intensity | 1135.8 | 1135.8 | 25.8 | 450.0 | 452.5 | 30.4 | 767.3 | 1428.6 | 11.6 |
| **Mowing**  **[No. cutting events]** | Number of plots | 30.0 | 30.0 | 30.0 | 30.0 | 27.0 | 32.0 | 32.0 | 28.0 | 23.0 |
|  | Mean intensity | 1.9 | 1.9 | 0.8 | 1.5 | 1.5 | 0.9 | 1.5 | 1.6 | 1.0 |
|  | Max intensity | 3.0 | 3.0 | 2.5 | 3.0 | 3.0 | 1.5 | 3.0 | 2.0 | 1.5 |
| **Time after mowing [days]** | Min. number of days |  |  | 11.0 |  |  | 10.5 |  |  | 16.5 |
|  | Mean number of days |  |  | 72.8 |  |  | 76.9 |  |  | 91.8 |
|  | Max. number of days |  |  | 145.5 |  |  | 114.0 |  |  | 136.0 |

Activities were calculated until the sampling day as described in the text of Appendix S1. The numbers of plots indicate the number of plots which were fertilized, grazed or mown. Means are only calculated for those plots.
