# Supplementary material for: Resource-Mediated Indirect Effects of Grassland Management on Arthropod Diversity
Source: PLoS One. 2014 Sep 4;9(9):e107033. doi: 10.1371/journal.pone.0107033 (PMC4154770; doi:10.1371/journal.pone.0107033)
Supplement: Appendix S1 — Assessment and calculation of land-use information. (DOC) [file pone.0107033.s005.doc]

Appendix S1: Land use information

Land-use information for the grasslands was obtained through questionnaires that were filled out by the land owners, together with a staff member of the Biodiversity Exploratory team, every year since 2006. This questionnaire covers all relevant information about the type and intensity of annual land use [1]. In particular, it covers both stable characteristics such as grassland size, and variable characteristics such as grazing days, livestock number, livestock type and age, mowing dates and machinery, and the type and amount of fertilizer applied. We calculated the intensity of management components by considering all measures in the two years preceding the sampling year, and all measures taken by the farmer up to the sampling date, e.g. for the 2008 sampling we included land-use measures in 2006 and 2007 and the months before the sampling in 2008. For the 2009 sampling land use in 2007-2009 was included accordingly. Arthropods were sampled twice per year (see section ‘Plant and arthropod sampling’ in the main text) in June and August but pooled for analysis. When a management action, e.g. cutting, took place in-between the two sampling events, this was accounted for as outlined below.

Fertilization:

Fertilization was calculated as the total amount of nitrogen applied as chemical fertilizer, manure or slurry. Conversion from m³ manure or slurry to kg N was done as outlined in Blüthgen *et al.* [2]: “Fertilization covered organic or inorganic fertilizer applied by farmers, but not excrements by grazing livestock, and was quantified by kg nitrogen (N) per hectare. Because of the low number of P-fertilized sites […] and inaccurate information on P-fertilization provided by farmers, we did not account for P fertilization. […] Moreover, we used total N irrespective of differences in availability to plants between organic and mineral fertilizer […]. When organic fertilizer was provided as volume […], we converted it to kg by multiplying with 3.2 kg nitrogen m^−3^ in case of cattle slurry and with 0.6 kg nitrogen m^−3^ in case of cattle manure (Timmermann and Siegfried, unpublished; see http://www.landwirtschaft-mlr.badenwuerttemberg.de/servlet/PB/menu/1043361 l1/index.html).” As fertilization always took place in early spring, calculations were identical for each of the two sampling dates and it was not necessary to calculate a mean for the analysis.

Grazing:

If the grazing period was overlapping with a sampling date, only the days prior to sampling were counted. As we sampled on two days per year (June and August), the mean grazing intensity was calculated relating to the two dates. The grazing intensity was standardized to plot size (0.25 ha) to account for different pasture sizes and was calculated as the product of grazing days and livestock units. Livestock units were estimated as described in Fischer *et al.* [1]: “For pastures and mown pastures, we recorded livestock species (cattle,horse, sheep, or goat) […]. In addition, to relate grazing data to livestock units we collected data about the age and density of the livestock (cattle < 1 year: 0.3 livestock units (LU), cattle 2 years: 0.6 LU, cattle > 2 years: 1 LU, sheep and goat < 1 year: 0.05 LU, sheep and goat > 1 year: 0.1 LU, horse < 3 years: 0.7 LU, horse > 3 years: 1.1 LU).”

Cutting:

Cutting intensity was included as the number of cutting events up to the sampling event, e.g. in a grassland that was on average mown twice per year, the total number of cuts in the two preceding years and in the sampling year up until the first sampling date was usually five. If an additional cutting took place between the two arthropod sampling days, we separately counted the cutting events prior to each sampling and averaged them. For example, to calculate cutting intensity for arthropod sampling in 2008, the average was 5.5 when there were two cutting events each in 2006 and 2007 and one cutting event in 2008 before first sampling and a second one between the first and second arthropod sampling. Cutting events that took place after the second arthropod sampling date were ignored.

Time after cutting:

For each of the two arthropod sampling dates, the number of days since the last cutting event were counted for each sampling date and averaged. If a plot was not cut in the sampling year or only after sampling, time after cutting was calculated as the number of days since the onset of the vegetation period, based on daily mean temperatures. About 75% of pastures were sampled more than 125 days after the onset of the vegetation period in 2008 and about the same proportion of plots was sampled fewer than 125 days after the onset of the vegetation period in 2009. To calculate the onset of the vegetation period, mean temperature was summed from January 1^st^ until the temperature sum reached a value of 200, which was defined as onset of the vegetation period. Only days with positive temperature means were considered. Temperature means were obtained from meteorological stations near the exploratory regions (run by the German Weather Service DWD). Positive temperature values in January were multiplied with 0.5 and those in February with 0.75 to control for effects of day length inhibiting early vegetation growth [3]. For an example see Table 1.

Table 1: Theoretical example for the calculation of vegetation onset. Calculations are based on the hypothetical daily mean temperature T_mean_. T_i_ is the temperature which is used for calculation (only positive values and corrected with 0.5 in January and 0.75 in February), T_sum_ is the sum over all previous T_i_. In this example, arthropod sampling is assumed to take place on the June 20^th^. (day 170 of the year). This example results in a value for Time after cutting of 102 (170-68 =102).

| Date | 1.1. | 2.1. | 3.1. | 4.1. | 5.1. | 6.1. | 7.1. | 8.1. | 9.1. | 10.1. | … |
| --- | --- | --- | --- | --- | --- | --- | --- | --- | --- | --- | --- |
| Yearday | 1 | 2 | 3 | 4 | 5 | 6 | 7 | 8 | 9 | 10 |  |
| T_mean_ | -5 | -4 | -2 | -0.5 | 0.5 | 0.5 | 2 | 3 | 2 | 3 |  |
| T_i_ | 0 | 0 | 0 | 0 | 0.25 | 0.25 | 1 | 1.5 | 1 | 1.5 |  |
| T_sum_ | 0 | 0 | 0 | 0 | 0.25 | 0.5 | 1.5 | 3 | 4 | 5.5 |  |
|  |  |  |  |  |  |  |  |  |  |  |  |
| Date | 1.2. | 2.2. | 3.2. | 4.2. | 5.2. | 6.2. | 7.2. | 8.2. | 9.2. | 10.2. | … |
| Yearday | 31 | 32 | 33 | 34 | 35 | 36 | 37 | 38 | 39 | 40 |  |
| T_mean_ | 5 | 7 | 3 | 4 | 2 | -1 | 1 | 3 | 5 | 4 |  |
| T_i_ | 3.75 | 5.25 | 2.25 | 3 | 1.5 | 0 | 0.75 | 2.25 | 4 | 3 |  |
| T_sum_ | 33.75 | 39 | 41.25 | 44.25 | 45.75 | 45.75 | 46.5 | 48.75 | 52.75 | 55.75 |  |
|  |  |  |  |  |  |  |  |  |  |  |  |
| Date | 1.3. | 2.3. | 3.3. | 4.3. | 5.3. | 6.3. | 7.3. | 8.3. |  |  |  |
| Yearday | 61 | 62 | 63 | 64 | 65 | 66 | 67 | **68** |  |  |  |
| T_mean_ | 10 | 9 | 10 | 13 | 8 | 11 | 12 | 10 |  |  |  |
| T_i_ | 10 | 9 | 10 | 13 | 8 | 11 | 12 | 10 |  |  |  |
| T_sum_ | 127.5 | 136.5 | 146.5 | 160 | 168 | 179 | 190 | **200** |  |  |  |

Literature cited

1. Fischer M, Bossdorf O, Gockel S, Hansel F, Hemp A, et al. (2010) Implementing large-scale and long-term functional biodiversity research: The Biodiversity Exploratories. Basic and Applied Ecology 11: 473-485.

2. Blüthgen N, Dormann CF, Prati D, Klaus VH, Kleinebecker T, et al. (2012) A quantitative index of land-use intensity in grasslands: Integrating mowing, grazing and fertilization. Basic and Applied Ecology 13: 207-220.

3. Waßhausen W (1987) Frühjahrspflege auf dem Grünland: Zehn Punkte beachten. Landwirtschaftsblatt Weser-Ems.
