# Supplementary material for: Resource-Mediated Indirect Effects of Grassland Management on Arthropod Diversity
Source: PLoS One. 2014 Sep 4;9(9):e107033. doi: 10.1371/journal.pone.0107033 (PMC4154770; doi:10.1371/journal.pone.0107033)
Supplement: Appendix S2 — Classification of trophic guilds. (DOC) [file pone.0107033.s006.doc]

Appendix S2: Classification of trophic guilds

Information on food resources of adult arthropods was derived from a literature search (Coleoptera) or provided through different databases which had been build up by taxonomic experts (Hemiptera, Araneae, Coleoptera and Orthoptera). As main reference, books on the ecology of species were used: Freude *et al.* [1]; Koch [2]; Müller-Motzfeld [3]; and Böhme [4] for Coleoptera. [5,6,and 7] for Auchenorrhyncha (Hemiptera), and [8] for Heteroptera (Hemiptera). The database for Araneae includes information from [9-12,and 13]. A database for Orthoptera, including information from [14,15,and 16] was provided by Frank Dziock (Orthoptera trait database “TRAITS for HOPPERS”; technical details and scope of this database will be published). For some Coleoptera species, data on feeding preferences were only available at the genus level. We then assumed that all species in the genera have the same feeding preference. For a species list and assigned feeding guilds as well as mean body size see Appendix F.

Literature cited

1. Freude H, Harde K, Lohse G, Lucht W (1976-2010) Die Käfer Mitteleuropas. Heidelberg: Spektrum Akademischer Verlag.

2. Koch K (1989-1996) Ökologie. - Die Käfer Mitteleuropas. Krefeld.

3. Müller-Motzfeld G (2004) Die Käfer Mitteleuropas. Bd. 2 Adephaga 1, Carabidae (Laufkäfer); Müller-Motzfeld G, editor. Heidelberg/Berlin.

4. Böhme J (2005) Die Käfer Mitteleuropas, Band K-Katalog: Faunistische Übersicht. Heroldsberg: Elsevier GmbH, Spektrum Akademischer Verlag.

5. Nickel H, Remane R (2002) Artenliste der Zikaden Deutschlands, mit Angaben zu Nährpflanzen, Lebenszyklen und Verbreitung (Hemiptera, Fulgoromorpha et Cicadomorpha). Beiträge zur Zikadenkunde 5: 27-64.

6. Nickel H (2003) The leafhoppers and planthoppers of Germany (Hemiptera, Auchenorrhyncha): Patterns and strategies in a highly diverse group of phytophagous insects. Sofia, Moskau: Pensoft Publishers.

7. Biedermann R, Niedringhaus R (2004) Die Zikaden Deutschlands. Scheßel: WABV Fründ.

8. Wachmann E, Melber A, Deckert J (2004-2012) Wanzen Band 1-5. Keltern: Goecke & Evers.

9. Maurer R, Hänggi A (1990) Katalog der schweizerischen Spinnen. Neuchâtel: Doc. Faun. Helv. 412 p.

10. Platen R, Moritz M, von Broen B (1991) Liste der Webspinnen- und Weberknechtarten (Arach.: Araneida, Opilionida) des Berliner Raumes und ihre Auswertung für Naturschutzzwecke (Rote Liste). In: Auhagen A, Platen R, Sukopp H, editors. Rote Listen der gefährdeten Pflanzen und Tiere in Berlin: Landschaftsentw. u. Umweltfr. pp. 169-205.

11. Platen R, von Broen B, Herrmann A, Ratschker UM, Sacher P (1999) Gesamtartenliste und Rote Liste der Webspinnen, Weberknechte und Pseudoskorpione (Araneae, Opiliones, Pseudoscorpiones) mit Angaben zur Häufigkeit und Ökologie. Natursch Landschaftspfl Brandenburg 8: Suppl: 1-79.

12. Hänggi A, Stöckli E, Nentwig W (1995) Lebensräume mitteleuropäischer Spinnen. Charakterisierung der Lebensräume der häufigsten Spinnenarten Mitteleuropas und der mit diesen vergesellschafteten Arten. Miscellanea Faunistica Helvetiae 4: 1-459.

13. Buchar J, Ruzicka V (2002) Catalogue of spiders of the Czech Republic. Praha: Peres Publishers.

14. Köhler G (2001) Fauna der Heuschrecken (Ensifera et Caelifera) des Freistaates Thüringen; Fritzlar F, Samietz J, editors. Thüringer Landesanstalt für Umwelt und Geologie: Jena.

15. Maas S, Detzel P, Staudt A (2002) Gefährdungsanalyse der Heuschrecken Deutschlands: Verbreitungsatlas, Gefährdungseinstufung und Schutzkonzepte. Bonn, Germany: Bundesamt für Naturschutz.

16. Baur B, Baur H, Roesti C, Roesti D (2006) Die Heuschrecken der Schweiz. Bern: Haupt Verlag.
