# Supplementary material for: Resource-Mediated Indirect Effects of Grassland Management on Arthropod Diversity
Source: PLoS One. 2014 Sep 4;9(9):e107033. doi: 10.1371/journal.pone.0107033 (PMC4154770; doi:10.1371/journal.pone.0107033)
Supplement: Appendix S3 — Structural equation model setup and path-selection procedure. (DOC) [file pone.0107033.s007.doc]

Appendix S3: Structural equation modeling

Data transformation

A log-transformation (after adding a constant factor of 0.1) was applied to all plant and arthropod data and the land-use variables to restore normality, with few exceptions. One exception was ‘time after mowing’ that followed a bimodal distribution. Conversion to a categorical variable (more or less than 100 days) resulted in significantly worse model fit; we therefore kept the original data. Plant biomass data was already normally distributed and was therefore also not transformed. Log-transformation did not result in strictly normally distributed land-use data as the frequency of zeros in the data sets is high. Nevertheless the log-transformed data were used, because non-zero values were normally distributed after transformation and zeros represented real data points and not a measurement error. Normality was tested using the function shapiro.test in R, to assess whether the data are significantly (with an alpha-level of 0.05) different from a normal distribution based on the data’s mean and standard deviation.

Model selection procedure

As a measure of model fit, the overall model p-value was used following [1, p.128f]), estimated from a chi-square comparison between the covariance matrices of the data and the model. If this p-value was smaller than 0.05, the two covariance matrices were considered to be significantly different from one another and the model was therefore not describing the data accurately. In this case, a step-wise selection procedure was applied by hand during which the most non-significant interaction path (greatest p-value) was omitted from the model and the new model was compared to the previous model, using the corrected Akaike’s Information Criterion (AICc). This procedure was applied until the AICc did not decrease further. We additionally considered the Goodness-of-fit index on rejected models (p<0.05).

The sample sizes within the three regions were too small to achieve an appropriate n/p ratio of 5 or higher (n=sample size, p=number parameters in the model) as recommended by [1]. Therefore, we tested whether a valid general model structure exists which would allow us to include all regions together in one model. We fitted only the ‘land use and plant data’ part of the full model based on data from 2008. After fitting the ‘land use and plant’ model and deleting the same non-significant paths (fertilization and cutting on plant biomass) for all three regions, we found a model structure which fit the data from all regions (Schwäbische Alb: Chi²_min_=1.47 p=0.477; Hainich-Dün: Chi²_min_=1.845 p=0.397; Schorfheide-Chorin: Chi²_min_=2.298 p=0.317). Adding the ‘arthropod part’ to the model structure resulted in significant differences between the model and the data for all three regions. Further deleting non-significant paths resulted in an acceptable model (p>0.05) only for the region Hainich-Dün (Df=30, Chi²_min_=37.35, p=0.167; Schwäbische Alb: Df=22, Chi²_min_=43.98, p=0.004; Schorfheide-Chorin: Df=32, Chi²_min_=53.80, p=0.009). Although the Goodness-of-fit index was still high (0.82-0.84), we rejected this approach because the models had only been improved by changes in the land use and plant part of the model. The same was true for the second model (resource abundance model). In the following, our two initial models were fit and the step-wise selection procedure was applied for each region separately.

Path definition

Most land-use components are not expected to have direct effects on the insect measures, e.g. fertilization effects will act through the food chain, disturbance of insects by grazing is only moderate and lethal effects of mowing are accounted for by the additional variable ‘time after cutting’, that is positively correlated with the mowing frequency as each additional cutting event decreases the time span between cutting and sampling. For the full initial model structure see Figure S1.

Models with arthropod abundance

To test if the missing effect between plant biomass and arthropod biomass was driven by a shift in the abundances of differently sized species (more small species instead of some large species) we tested the ‘resource abundance model’ including arthropod abundances instead of biomass (Figure S2) but results were similar.

Literature cited

1. Grace JB (2006) Structural Equation Modeling and Natural Systems. Cambridge, UK: Cambridge University Press.
