# Supplementary material for: Resource-Mediated Indirect Effects of Grassland Management on Arthropod Diversity
Source: PLoS One. 2014 Sep 4;9(9):e107033. doi: 10.1371/journal.pone.0107033 (PMC4154770; doi:10.1371/journal.pone.0107033)
Supplement: Appendix S4 — Bivariate correlations between model variables. (DOC) [file pone.0107033.s008.doc]

Appendix S4: Bivariate correlations

Figure S1: Bivariate correlations including plant diversity and data from 2008. Correlations are shown between plant diversity (measured as species richness) and plant biomass, herbivore and predator diversity (both measured as species richness). Graphs are based on untransformed data.

Figure S2: Bivariate correlations including plant diversity and data from 2009. Correlations are shown between plant diversity (measured as species richness) and plant biomass, herbivore and predator diversity (both measured as species richness). Graphs are based on untransformed data.

Figure S3: Bivariate correlations including herbivore and predator diversity from 2008. Correlations are shown between plant biomass, herbivore diversity and predator biomass and herbivore and predator diversity. Diversities measured as species richness. Graphs are based on untransformed data.

Figure S4: Bivariate correlations including herbivore and predator diversity from 2009. Correlations are shown between plant biomass, herbivore diversity and predator biomass and herbivore and predator diversity. Diversities measured as species richness. Graphs are based on untransformed data.

Figure S5: Bivariate correlations including herbivore biomass from 2008. Correlations are shown between herbivore biomass and herbivore and predator diversity (measured as species richness) as well as with predator. Graphs are based on untransformed data.

Figure S6: Bivariate correlations including herbivore biomass from 2009. Correlations are shown between herbivore biomass and herbivore and predator diversity (measured as species richness) as well as with predator. Graphs are based on untransformed data.
