# Supplementary material for: Resource-Mediated Indirect Effects of Grassland Management on Arthropod Diversity
Source: PLoS One. 2014 Sep 4;9(9):e107033. doi: 10.1371/journal.pone.0107033 (PMC4154770; doi:10.1371/journal.pone.0107033)
Supplement: Appendix S5 — List of arthropod species sampled in 2008 (PDF) [file pone.0107033.s009.pdf]

List of species sampled in 2008

								Abundance			Number of plots		
Order	Suborder	Family	Genus/Species	Author/Year	Feeding guild	mean body length [mm]	estimated biomass [g]	Swabian Alb	Hainich-Dün	Schorfheide-Chorin	Swabian Alb	Hainich-Dün	Schorfheide-Chorin
Araneae	Labodignatha	Araneidae	*Aculepeira ceropegia*	Walckenaer, 1802	predator	11.00	163.21	12	1	0	7	1	0
Araneae	Labodignatha	Araneidae	*Agalenatea redii*	(Scopoli, 1763)	predator	6.93	48.64	0	1	1	0	1	1
Araneae	Labodignatha	Araneidae	*Araneus diadematus*	Clerck, 1757	predator	11.88	199.67	7	1	3	6	1	3
Araneae	Labodignatha	Araneidae	*Araneus quadratus*	Clerck, 1757	predator	12.25	216.38	0	6	0	0	2	0
Araneae	Labodignatha	Araneidae	*Araneus triguttatus*	Fabricius, 1775	predator	5.25	23.50	1	0	0	1	0	0
Araneae	Labodignatha	Araneidae	*Araniella cucurbitina*	(Clerck, 1757)	predator	5.50	26.55	4	4	0	4	4	0
Araneae	Labodignatha	Araneidae	*Araniella opisthographa*	(Kulczynski, 1905)	predator	4.38	14.62	0	1	0	0	1	0
Araneae	Labodignatha	Araneidae	*Argiope bruennichi*	(Scopoli, 1772)	predator	11.78	195.30	0	3	3	0	1	2
Araneae	Labodignatha	Araneidae	*Cyclosa oculata*	(Walckenaer, 1802)	predator	5.08	21.56	1	0	0	1	0	0
Araneae	Labodignatha	Araneidae	*Hypsosinga albovittata*	(Westring, 1851)	predator	4.15	12.69	4	0	0	2	0	0
Araneae	Labodignatha	Araneidae	*Hypsosinga sanguinea*	(C.L. Koch, 1844)	predator	3.75	9.73	1	0	0	1	0	0
Araneae	Labodignatha	Araneidae	*Mangora acalypha*	(Walckenaer, 1802)	predator	4.50	15.69	20	10	37	11	10	18
Araneae	Labodignatha	Clubionidae	*Clubiona diversa*	O. P.-Cambridge, 1862	predator	4.00	11.53	2	0	0	2	0	0
Araneae	Labodignatha	Clubionidae	*Clubiona stagnatilis*	Kulczynski, 1897	predator	6.25	37.11	0	0	1	0	0	1
Araneae	Labodignatha	Linyphiidae	*Araeoncus humilis*	(Blackwall, 1841)	predator	1.60	1.04	1	5	3	1	3	3
Araneae	Labodignatha	Linyphiidae	*Bathyphantes gracilis*	(Blackwall, 1841)	predator	1.98	1.83	0	19	13	0	9	6
Araneae	Labodignatha	Linyphiidae	*Bathyphantes parvulus*	(Westring, 1851)	predator	2.15	2.27	0	2	0	0	2	0
Araneae	Labodignatha	Linyphiidae	*Dismodicus bifrons*	(Blackwall, 1841)	predator	2.11	2.16	1	0	0	1	0	0
Araneae	Labodignatha	Linyphiidae	*Dismodicus elevatus*	(C.L. Koch, 1838)	predator	2.48	3.29	1	0	0	1	0	0
Araneae	Labodignatha	Linyphiidae	*Erigone atra*	Blackwall, 1833	predator	2.25	2.55	5	21	13	4	14	9
Araneae	Labodignatha	Linyphiidae	*Erigone dentipalpis*	(Wider, 1834)	predator	2.33	2.80	13	18	5	9	12	5
Araneae	Labodignatha	Linyphiidae	*Hypomma cornutum*	(Blackwall, 1833)	predator	2.40	3.02	0	0	2	0	0	2
Araneae	Labodignatha	Linyphiidae	*Linyphia triangularis*	(Clerck, 1757)	predator	6.00	33.35	2	2	0	2	2	0
Araneae	Labodignatha	Linyphiidae	*Meioneta rurestris*	(C.L. Koch, 1836)	predator	6.23	36.80	8	8	3	6	7	3
Araneae	Labodignatha	Linyphiidae	*Mermessus trilobatus*	(Emerton, 1882)	predator	1.85	1.53	0	9	0	0	7	0
Araneae	Labodignatha	Linyphiidae	*Micrargus subaequalis*	(Westring, 1851)	predator	1.83	1.49	0	1	0	0	1	0
Araneae	Labodignatha	Linyphiidae	*Microlinyphia pusilla*	(Sundevall, 1830)	predator	3.88	10.64	15	2	11	7	2	10
Araneae	Labodignatha	Linyphiidae	*Minyriolus pusillus*	(Wider, 1834)	predator	1.25	0.55	0	0	2	0	0	2
Araneae	Labodignatha	Linyphiidae	*Nusoncus nasutus*	(Wunderlich, 2008)	predator	1.90	1.64	2	0	0	2	0	0
Araneae	Labodignatha	Linyphiidae	*Oedothorax apicatus*	(Blackwall, 1850)	predator	2.55	3.54	3	43	2	3	21	2
Araneae	Labodignatha	Linyphiidae	*Palliduphantes pallidus*	(O. P.-Cambridge, 1871)	predator	1.95	1.75	0	1	0	0	1	0
Araneae	Labodignatha	Linyphiidae	*Pelecopsis parallela*	(Wider, 1834)	predator	1.45	0.81	1	0	0	1	0	0
Araneae	Labodignatha	Linyphiidae	*Porrhomma pygmaeum*	(Blackwall, 1834)	predator	2.28	2.64	0	0	2	0	0	2
Araneae	Labodignatha	Linyphiidae	*Tenuiphantes mengei*	(Kulczynski, 1887)	predator	2.03	1.95	2	1	0	2	1	0
Araneae	Labodignatha	Linyphiidae	*Tenuiphantes tenuis*	(Blackwall, 1852)	predator	2.88	4.87	6	72	4	4	22	4
Araneae	Labodignatha	Linyphiidae	*Tiso vagans*	(Blackwall, 1834)	predator	2.03	1.95	8	3	3	6	3	3
Araneae	Labodignatha	Linyphiidae	*Trematocephalus cristatus*	(Wider, 1834)	predator	2.30	2.70	0	0	1	0	0	1
Araneae	Labodignatha	Lycosidae	*Pardosa amentata*	(Clerck, 1757)	predator	6.25	37.11	1	0	0	1	0	0
Araneae	Labodignatha	Lycosidae	*Pardosa lugubris*	(Walckenaer, 1802)	predator	5.60	27.83	0	1	0	0	1	0
Araneae	Labodignatha	Lycosidae	*Pardosa paludicola*	(Clerck, 1757)	predator	7.63	62.59	2	0	0	1	0	0
Araneae	Labodignatha	Lycosidae	*Pardosa palustris*	(Linnaeus, 1758)	predator	6.00	33.35	4	0	6	3	0	4
Araneae	Labodignatha	Lycosidae	*Pardosa prativaga*	L. Koch, 1870	predator	6.10	34.82	0	0	1	0	0	1
Araneae	Labodignatha	Lycosidae	*Pardosa pullata*	(Clerck, 1757)	predator	4.75	18.08	1	0	1	1	0	1
Araneae	Labodignatha	Philodromidae	*Philodromus albidus*	Kulczynski, 1911	predator	4.12	12.45	2	0	1	2	0	1
Araneae	Labodignatha	Philodromidae	*Philodromus aureolus*	(Clerck, 1757)	predator	5.63	28.22	0	1	0	0	1	0
Araneae	Labodignatha	Philodromidae	*Philodromus cespitum*	(Walckenaer, 1802)	predator	4.78	18.38	1	1	1	1	1	1
Araneae	Labodignatha	Philodromidae	*Philodromus collinus*	C.L. Koch, 1835	predator	4.88	19.41	1	0	0	1	0	0
Araneae	Labodignatha	Philodromidae	*Thanatus formicinus*	(Clerck, 1757)	predator	7.88	68.11	1	0	0	1	0	0
Araneae	Labodignatha	Philodromidae	*Tibellus oblongus*	(Franganillo, 1926)	predator	7.63	62.59	0	4	2	0	4	2
Araneae	Labodignatha	Salticidae	*Evarcha arcuata*	(Clerck, 1757)	predator	6.35	38.69	3	0	0	2	0	0
Araneae	Labodignatha	Salticidae	*Heliophanus flavipes*	(Hahn, 1832)	predator	4.50	15.69	1	1	0	1	1	0

List of species sampled in 2008

								Abundance			Number of plots		
Order	Suborder	Family	Genus/Species	Author/Year	Feeding guild	mean body length [mm]	estimated biomass [g]	Swabian Alb	Hainich-Dün	Schorfheide-Chorin	Swabian Alb	Hainich-Dün	Schorfheide-Chorin
Araneae	Labodignatha	Salticidae	*Synageles venator*	(Lucas, 1836)	predator	3.50	8.12	0	1	0	0	1	0
Araneae	Labodignatha	Tetragnathidae	*Metellina mengei*	(Blackwall, 1870)	predator	4.88	19.41	2	0	0	2	0	0
Araneae	Labodignatha	Tetragnathidae	*Metellina segmentata*	(Clerck, 1757)	predator	7.50	59.84	1	0	0	1	0	0
Araneae	Labodignatha	Tetragnathidae	*Pachygnatha clercki*	Sundevall, 1823	predator	5.25	23.50	0	0	6	0	0	3
Araneae	Labodignatha	Tetragnathidae	*Pachygnatha degeeri*	Sundevall, 1830	predator	3.55	8.43	22	3	20	14	3	6
Araneae	Labodignatha	Tetragnathidae	*Tetragnatha extensa*	(Linnaeus, 1758)	predator	8.05	72.03	2	0	19	2	0	12
Araneae	Labodignatha	Tetragnathidae	*Tetragnatha obtusa*	C.L. Koch, 1837	predator	5.43	25.67	0	1	0	0	1	0
Araneae	Labodignatha	Theridiidae	*Anelosimus vittatus*	(C.L. Koch, 1836)	predator	3.53	8.31	0	0	10	0	0	5
Araneae	Labodignatha	Theridiidae	*Asagena phalerata*	(Panzer, 1801)	predator	4.93	19.93	1	0	0	1	0	0
Araneae	Labodignatha	Theridiidae	*Cryptachaea riparia*	(Blackwall, 1834)	predator	3.50	8.12	1	0	0	1	0	0
Araneae	Labodignatha	Theridiidae	*Dipoena torva*	(Thorell, 1875)	predator	3.25	6.69	0	0	1	0	0	1
Araneae	Labodignatha	Theridiidae	*Enoplognatha latimana*	Hippa & Oksala, 1982	predator	4.50	15.69	0	1	0	0	1	0
Araneae	Labodignatha	Theridiidae	*Enoplognatha ovata*	(Clerck, 1757)	predator	5.30	24.09	2	0	0	2	0	0
Araneae	Labodignatha	Theridiidae	*Neottiura bimaculata*	(Linnaeus, 1767)	predator	2.50	3.36	2	1	10	2	1	7
Araneae	Labodignatha	Theridiidae	*Paidiscura pallens*	(Blackwall, 1834)	predator	1.58	1.01	1	8	0	1	6	0
Araneae	Labodignatha	Theridiidae	*Phylloneta impressa*	(L. Koch, 1881)	predator	4.25	13.51	113	77	10	31	34	8
Araneae	Labodignatha	Theridiidae	*Platnickina tincta*	(Walckenaer, 1802)	predator	3.30	6.96	1	1	0	1	1	0
Araneae	Labodignatha	Theridiidae	*Robertus neglectus*	(O. P.-Cambridge, 1871)	predator	2.13	2.21	0	3	0	0	2	0
Araneae	Labodignatha	Theridiidae	*Theridion pinastri*	L. Koch, 1872	predator	3.13	6.06	2	1	0	2	1	0
Araneae	Labodignatha	Theridiidae	*Theridion varians*	Hahn, 1833	predator	3.13	6.06	1	0	0	1	0	0
Araneae	Labodignatha	Thomisidae	*Ebrechtella tricuspidata*	(Fabricius, 1775)	predator	4.50	15.69	0	0	1	0	0	1
Araneae	Labodignatha	Thomisidae	*Misumena vatia*	(Clerck, 1757)	predator	6.63	43.32	1	0	0	1	0	0
Araneae	Labodignatha	Thomisidae	*Ozyptila praticola*	(C.L. Koch, 1837)	predator	3.45	7.82	0	0	1	0	0	1
Araneae	Labodignatha	Thomisidae	*Xysticus bifasciatus*	C.L. Koch, 1837	predator	7.03	50.50	1	0	1	1	0	1
Araneae	Labodignatha	Thomisidae	*Xysticus cristatus*	(Clerck, 1757)	predator	5.58	27.57	11	2	3	9	2	3
Araneae	Labodignatha	Thomisidae	*Xysticus kochi*	Thorell, 1872	predator	6.55	41.96	2	0	3	2	0	3
Araneae	Labodignatha	Thomisidae	*Xysticus lanio*	C.L. Koch, 1835	predator	6.13	35.27	0	1	0	0	1	0
Araneae	Labodignatha	Thomisidae	*Xysticus ulmi*	(Hahn, 1832)	predator	5.08	21.56	0	0	2	0	0	2
Coleoptera	Adephaga	Carabidae	*Amara aulica*	(Panzer, 1797)	herbivore	12.75	240.29	0	2	0	0	1	0
Coleoptera	Adephaga	Carabidae	*Amara bifrons*	(Gyllenhal, 1810)	herbivore	6.50	41.13	0	0	1	0	0	1
Coleoptera	Adephaga	Carabidae	*Amara communis*	(Panzer, 1797)	herbivore	7.00	49.94	1	0	0	1	0	0
Coleoptera	Adephaga	Carabidae	*Amara familiaris*	(Duftschmid, 1812)	herbivore	6.50	41.13	2	0	0	2	0	0
Coleoptera	Adephaga	Carabidae	*Amara montivaga*	Sturm, 1825	herbivore	8.50	83.06	0	3	0	0	1	0
Coleoptera	Adephaga	Carabidae	*Amara plebeja*	(Gyllenhal, 1810)	herbivore	7.00	49.94	0	0	5	0	0	4
Coleoptera	Adephaga	Carabidae	*Ophonus puncticeps*	(Stephens, 1828)	herbivore	8.25	76.81	1	4	0	1	3	0
Coleoptera	Adephaga	Carabidae	*Poecilus cupreus*	(Linnaeus, 1758)	predator	11.00	163.21	1	0	5	1	0	3
Coleoptera	Adephaga	Carabidae	*Poecilus lepidus*	(Leske, 1785)	predator	12.00	205.00	1	0	0	1	0	0
Coleoptera	Adephaga	Carabidae	*Poecilus versicolor*	(Sturm, 1824)	predator	9.75	118.98	2	0	6	2	0	5
Coleoptera	Adephaga	Carabidae	*Trechus obtusus*	Erichson, 1837	predator	4.00	11.53	0	1	12	0	1	5
Coleoptera	Adephaga	Carabidae	*Trechus quadristriatus*	(Schrank, 1781)	predator	4.00	11.53	1	1	26	1	1	7
Coleoptera	Polyphaga	Anthicidae	*Omonadus floralis*	(Linnaeus, 1758)	predator	3.25	6.69	0	0	3	0	0	2
Coleoptera	Polyphaga	Anthicidae	*Omonadus formicarius*	(Goeze, 1777)	predator	3.15	6.16	0	1	0	0	1	0
Coleoptera	Polyphaga	Apionidae	*Acanephodus onopordi*	(Kirby, 1808)	herbivore	2.65	3.92	12	6	14	4	4	7
Coleoptera	Polyphaga	Apionidae	*Apion cruentatum*	Walton, 1844	herbivore	3.00	5.42	1	1	0	1	1	0
Coleoptera	Polyphaga	Apionidae	*Apion frumentarium*	Linnaeus, 1758	herbivore	3.90	10.79	11	3	4	7	3	2
Coleoptera	Polyphaga	Apionidae	*Apion haematodes*	Kirby, 1808	herbivore	2.65	3.92	2	0	0	2	0	0
Coleoptera	Polyphaga	Apionidae	*Catapion pubescens*	(Kirby, 1811)	herbivore	1.95	1.75	36	38	2	15	18	2
Coleoptera	Polyphaga	Apionidae	*Catapion seniculus*	(Kirby, 1808)	herbivore	1.80	1.42	0	6	0	0	1	0
Coleoptera	Polyphaga	Apionidae	*Ceratapion gibbirostre*	(Gyllenhal, 1813)	herbivore	2.55	3.54	0	1	0	0	1	0
Coleoptera	Polyphaga	Apionidae	*Cyanapion gyllenhalii*	Kirby, 1808	herbivore	2.60	3.73	0	10	0	0	4	0
Coleoptera	Polyphaga	Apionidae	*Cyanapion platalea*	(Germar, 1817)	herbivore	2.10	2.13	0	2	0	0	1	0

List of species sampled in 2008

Order	Suborder	Family	Genus/Species	Author/Year	Feeding guild	mean body length [mm]	estimated biomass [g]	Abundance			Number of plots		
								Swabian Alb	Hainich-Dün	Schorfheide-Chorin	Swabian Alb	Hainich-Dün	Schorfheide-Chorin
Coleoptera	Polyphaga	Apionidae	*Cyanapion spencii*	(Kirby, 1808)	herbivore	2.20	2.41	1	20	14	1	14	11
Coleoptera	Polyphaga	Apionidae	*Diplapion confluens*	(Kirby, 1808)	herbivore	2.05	2.00	0	1	0	0	1	0
Coleoptera	Polyphaga	Apionidae	*Eutrichapion ervi*	(Kirby, 1808)	herbivore	2.20	2.41	0	1	0	0	1	0
Coleoptera	Polyphaga	Apionidae	*Eutrichapion punctigerum*	(Paykull, 1792)	herbivore	2.70	4.12	0	4	0	0	3	0
Coleoptera	Polyphaga	Apionidae	*Eutrichapion viciae*	(Paykull, 1800)	herbivore	2.15	2.27	0	3	0	0	3	0
Coleoptera	Polyphaga	Apionidae	*Holotrichapion ononis*	(Kirby, 1808)	herbivore	2.05	2.00	0	5	0	0	1	0
Coleoptera	Polyphaga	Apionidae	*Ischnopterapion loti*	(Kirby, 1808)	herbivore	2.25	2.55	2	2	0	1	1	0
Coleoptera	Polyphaga	Apionidae	*Ischnopterapion virens*	(Herbst, 1797)	herbivore	2.20	2.41	57	121	2	19	14	1
Coleoptera	Polyphaga	Apionidae	*Oxystoma cerdo*	(Gerstaecker, 1854)	herbivore	2.70	4.12	0	0	1	0	0	1
Coleoptera	Polyphaga	Apionidae	*Perapion curtirostre*	(Germar, 1817)	herbivore	2.15	2.27	3	0	9	2	0	2
Coleoptera	Polyphaga	Apionidae	*Perapion marchicum*	(Herbst, 1797)	herbivore	1.95	1.75	2	0	2	1	0	2
Coleoptera	Polyphaga	Apionidae	*Perapion violaceum*	(Kirby, 1808)	herbivore	3.05	5.66	1	0	3	1	0	2
Coleoptera	Polyphaga	Apionidae	*Protapion apricans*	(Herbst, 1797)	herbivore	2.45	3.19	198	34	2	24	15	2
Coleoptera	Polyphaga	Apionidae	*Protapion assimile*	Kirby, 1808	herbivore	2.05	2.00	26	58	1	11	13	1
Coleoptera	Polyphaga	Apionidae	*Protapion fulvipes*	(Geoffroy, 1785)	herbivore	2.00	1.87	47	22	8	16	12	4
Coleoptera	Polyphaga	Apionidae	*Protapion nigritarse*	(Kirby, 1808)	herbivore	1.75	1.32	0	43	0	0	5	0
Coleoptera	Polyphaga	Apionidae	*Protapion trifolii*	(Linnaeus, 1768)	herbivore	1.90	1.64	1	8	2	1	3	1
Coleoptera	Polyphaga	Apionidae	*Pseudoperapion brevirostre*	(Herbst, 1797)	herbivore	2.05	2.00	0	1	0	0	1	0
Coleoptera	Polyphaga	Apionidae	*Squamapion atomarium*	(Kirby, 1808)	herbivore	1.40	0.74	1	0	1	1	0	1
Coleoptera	Polyphaga	Apionidae	*Stenopterapion meliloti*	(Kirby, 1808)	herbivore	2.75	4.32	0	2	0	0	1	0
Coleoptera	Polyphaga	Apionidae	*Synapion ebeninum*	(Kirby, 1808)	herbivore	2.40	3.02	1	2	0	1	2	0
Coleoptera	Polyphaga	Apionidae	*Taeniapion urticarium*	(Herbst, 1784)	herbivore	2.10	2.13	0	0	1	0	0	1
Coleoptera	Polyphaga	Bruchidae	*Bruchidius marginalis*	(Fabricius, 1777)	herbivore	3.00	5.42	0	3	0	0	3	0
Coleoptera	Polyphaga	Bruchidae	*Bruchus atomarius*	(Linnaeus, 1761)	herbivore	2.75	4.32	5	0	0	4	0	0
Coleoptera	Polyphaga	Bruchidae	*Bruchus luteicornis*	Illiger, 1794	herbivore	2.15	2.27	0	1	0	0	1	0
Coleoptera	Polyphaga	Byrrhidae	*Byrrhus pilula*	(Linnaeus, 1758)	herbivore	8.00	70.86	1	0	0	1	0	0
Coleoptera	Polyphaga	Byturidae	*Byturus ochraceus*	(Scriba, 1790)	herbivore	4.30	13.93	1	0	0	1	0	0
Coleoptera	Polyphaga	Cantharidae	*Cantharis fusca*	Linnaeus, 1758	predator	13.00	252.83	1	0	5	1	0	5
Coleoptera	Polyphaga	Cantharidae	*Cantharis lateralis*	Linnaeus, 1758	predator	6.00	33.35	0	6	1	0	3	1
Coleoptera	Polyphaga	Cantharidae	*Cantharis livida*	Linnaeus, 1758	predator	11.25	173.11	2	2	0	2	1	0
Coleoptera	Polyphaga	Cantharidae	*Cantharis pallida*	Goeze, 1777	predator	7.25	54.75	0	1	2	0	1	2
Coleoptera	Polyphaga	Cantharidae	*Cratosilis denticollis*	(Schummel, 1844)	predator	6.50	41.13	9	110	1	4	21	1
Coleoptera	Polyphaga	Cantharidae	*Malthodes lobatus*	Kiesenwetter, 1852	predator	1.00	0.31	0	3	0	0	2	0
Coleoptera	Polyphaga	Cantharidae	*Metacantharis clypeata*	(Illiger, 1798)	predator	8.25	76.81	3	0	1	3	0	1
Coleoptera	Polyphaga	Cantharidae	*Rhagonycha fulva*	(Scopoli, 1763)	predator	8.50	83.06	0	1	0	0	1	0
Coleoptera	Polyphaga	Cerambycidae	*Phytoecia cylindrica*	(Linnaeus, 1758)	herbivore	9.00	96.48	1	0	0	1	0	0
Coleoptera	Polyphaga	Cholevidae	*Nargus velox*	(Spence, 1815)	predator	2.95	5.19	0	0	1	0	0	1
Coleoptera	Polyphaga	Cholevidae	*Ptomaphagus sericatus*	(Chaudoir, 1845)	predator	2.40	3.02	1	0	2	1	0	2
Coleoptera	Polyphaga	Cholevidae	*Sciodrepoides watsoni*	(Spence, 1815)	predator	3.00	5.42	1	0	2	1	0	2
Coleoptera	Polyphaga	Chrysomelidae	*Altica helianthemi*	(Allard, 1859)	herbivore	4.00	11.53	5	0	1	4	0	1
Coleoptera	Polyphaga	Chrysomelidae	*Altica oleracea*	(Linnaeus, 1758)	herbivore	3.00	5.42	20	1	9	10	1	5
Coleoptera	Polyphaga	Chrysomelidae	*Aphthona atrovirens*	(Forster, 1849)	herbivore	1.55	0.96	1	0	0	1	0	0
Coleoptera	Polyphaga	Chrysomelidae	*Aphthona cyparissiae*	(Koch, 1803)	herbivore	4.00	11.53	4	0	0	2	0	0
Coleoptera	Polyphaga	Chrysomelidae	*Aphthona herbigrada*	(Curtis, 1837)	herbivore	1.90	1.64	2	0	0	2	0	0
Coleoptera	Polyphaga	Chrysomelidae	*Aphthona pallida*	(Bach, 1856)	herbivore	1.65	1.13	2	1	0	2	1	0
Coleoptera	Polyphaga	Chrysomelidae	*Aphthona venustula*	(Kutschera, 1861)	herbivore	2.10	2.13	11	0	0	3	0	0
Coleoptera	Polyphaga	Chrysomelidae	*Asiorestia ferruginea*	(Scopoli, 1763)	herbivore	3.25	6.69	139	15	38	21	7	18
Coleoptera	Polyphaga	Chrysomelidae	*Asiorestia transversa*	(Marsham, 1802)	herbivore	4.25	13.51	8	0	5	5	0	4
Coleoptera	Polyphaga	Chrysomelidae	*Cassida hemisphaerica*	Herbst, 1799	herbivore	4.50	15.69	1	0	0	1	0	0
Coleoptera	Polyphaga	Chrysomelidae	*Cassida rubiginosa*	Muller, 1776	herbivore	6.75	45.40	1	0	0	1	0	0
Coleoptera	Polyphaga	Chrysomelidae	*Cassida vibex*	Linnaeus, 1767	herbivore	6.25	37.11	0	0	2	0	0	2

List of species sampled in 2008

Order	Suborder	Family	Genus/Species	Author/Year	Feeding guild	mean body length [mm]	estimated biomass [g]	Abundance			Number of plots		
								Swabian Alb	Hainich-Dün	Schorfheide-Chorin	Swabian Alb	Hainich-Dün	Schorfheide-Chorin
Coleoptera	Polyphaga	Chrysomelidae	*Chaetocnema concinna*	(Marsham, 1802)	herbivore	2.10	2.13	6	6	32	5	6	6
Coleoptera	Polyphaga	Chrysomelidae	*Chaetocnema hortensis*	(Geoffroy, 1785)	herbivore	1.90	1.64	9	11	55	5	9	14
Coleoptera	Polyphaga	Chrysomelidae	*Chaetocnema laevicollis*	(Thomson, 1866)	herbivore	2.00	1.87	2	0	0	2	0	0
Coleoptera	Polyphaga	Chrysomelidae	*Chrysolina fastuosa*	(Scopoli, 1763)	herbivore	5.50	26.55	1	0	6	1	0	1
Coleoptera	Polyphaga	Chrysomelidae	*Chrysolina oricalcia*	(Muller O.F., 1776)	herbivore	7.50	59.84	1	0	0	1	0	0
Coleoptera	Polyphaga	Chrysomelidae	*Clytra laeviuscula*	Ratzeburg, 1837	herbivore	9.00	96.48	0	15	0	0	5	0
Coleoptera	Polyphaga	Chrysomelidae	*Coptocephala rubicunda*	(Laicharting, 1781)	herbivore	5.25	23.50	1	0	0	1	0	0
Coleoptera	Polyphaga	Chrysomelidae	*Cryptocephalus aureolus*	Suffrian, 1847	herbivore	6.00	33.35	2	13	0	2	5	0
Coleoptera	Polyphaga	Chrysomelidae	*Cryptocephalus fulvus*	Goeze, 1777	herbivore	2.50	3.36	1	67	12	1	1	3
Coleoptera	Polyphaga	Chrysomelidae	*Cryptocephalus moraei*	(Linnaeus, 1758)	herbivore	4.00	11.53	0	3	0	0	2	0
Coleoptera	Polyphaga	Chrysomelidae	*Cryptocephalus sericeus*	(Linnaeus, 1758)	herbivore	6.10	34.82	3	1	0	1	1	0
Coleoptera	Polyphaga	Chrysomelidae	*Cryptocephalus vittatus*	Fabricius, 1775	herbivore	3.75	9.73	4	0	0	3	0	0
Coleoptera	Polyphaga	Chrysomelidae	*Derocrepis rufipes*	(Linnaeus, 1758)	herbivore	3.30	6.96	5	1	0	2	1	0
Coleoptera	Polyphaga	Chrysomelidae	*Dibolia cryptocephala*	(Koch, 1803)	herbivore	1.90	1.64	11	0	0	5	0	0
Coleoptera	Polyphaga	Chrysomelidae	*Dibolia occultans*	(Koch, 1803)	herbivore	3.00	5.42	1	0	0	1	0	0
Coleoptera	Polyphaga	Chrysomelidae	*Galeruca tanaceti*	(Linnaeus, 1758)	herbivore	8.00	70.86	6	2	0	3	2	0
Coleoptera	Polyphaga	Chrysomelidae	*Gastrophysa polygoni*	(Linnaeus, 1758)	herbivore	4.50	15.69	0	0	2	0	0	2
Coleoptera	Polyphaga	Chrysomelidae	*Gastrophysa viridula*	(De Geer, 1775)	herbivore	5.00	20.68	6	0	0	2	0	0
Coleoptera	Polyphaga	Chrysomelidae	*Labidostomis longimana*	(Linnaeus, 1761)	herbivore	5.25	23.50	2	1	1	2	1	1
Coleoptera	Polyphaga	Chrysomelidae	*Longitarsus atricillus*	(Linnaeus, 1761)	herbivore	2.50	3.36	0	1	0	0	1	0
Coleoptera	Polyphaga	Chrysomelidae	*Longitarsus ganglbaueri*	Heikertinger, 1912	herbivore	2.00	1.87	0	1	0	0	1	0
Coleoptera	Polyphaga	Chrysomelidae	*Longitarsus luridus*	(Scopoli, 1763)	herbivore	1.85	1.53	163	234	20	28	27	9
Coleoptera	Polyphaga	Chrysomelidae	*Longitarsus lycopi*	(Foudras, 1860)	herbivore	2.00	1.87	1	0	0	1	0	0
Coleoptera	Polyphaga	Chrysomelidae	*Longitarsus melanocephalus*	(De Geer, 1775)	herbivore	2.50	3.36	3	1	9	2	1	7
Coleoptera	Polyphaga	Chrysomelidae	*Longitarsus pratensis*	(Panzer, 1794)	herbivore	1.60	1.04	108	811	73	13	37	12
Coleoptera	Polyphaga	Chrysomelidae	*Longitarsus rubiginosus*	(Foudras, 1860)	herbivore	1.25	0.55	0	0	1	0	0	1
Coleoptera	Polyphaga	Chrysomelidae	*Longitarsus succineus*	(Foudras, 1860)	herbivore	1.95	1.75	22	44	32	8	3	17
Coleoptera	Polyphaga	Chrysomelidae	*Longitarsus tabidus*	(Fabricius, 1775)	herbivore	3.50	8.12	30	66	14	13	19	6
Coleoptera	Polyphaga	Chrysomelidae	*Oulema duftschmidi*	(Redtenbacher, 1874)	herbivore	4.25	13.51	3	1	131	2	1	18
Coleoptera	Polyphaga	Chrysomelidae	*Oulema gallaeciana*	(Heyden, 1870)	herbivore	4.00	11.53	3	2	5	3	2	4
Coleoptera	Polyphaga	Chrysomelidae	*Oulema melanopus*	(Linnaeus, 1758)	herbivore	4.00	11.53	11	0	26	9	0	5
Coleoptera	Polyphaga	Chrysomelidae	*Phyllotreta nemorum*	(Linnaeus, 1758)	herbivore	2.75	4.32	0	0	1	0	0	1
Coleoptera	Polyphaga	Chrysomelidae	*Phyllotreta striolata*	(Fabricius, 1803)	herbivore	1.90	1.64	1	0	0	1	0	0
Coleoptera	Polyphaga	Chrysomelidae	*Phyllotreta undulata*	Kutschera, 1860	herbivore	2.15	2.27	1	2	0	1	1	0
Coleoptera	Polyphaga	Chrysomelidae	*Phyllotreta vittula*	(Redtenbacher, 1849)	herbivore	1.65	1.13	1	7	20	1	6	9
Coleoptera	Polyphaga	Chrysomelidae	*Prasocuris glabra*	(Herbst, 1783)	herbivore	4.00	11.53	1	0	0	1	0	0
Coleoptera	Polyphaga	Chrysomelidae	*Psylliodes attenuatus*	(Koch, 1803)	herbivore	2.50	3.36	0	0	1	0	0	1
Coleoptera	Polyphaga	Chrysomelidae	*Psylliodes chrysocephalus*	(Linnaeus, 1758)	herbivore	3.00	5.42	1	0	5	1	0	4
Coleoptera	Polyphaga	Chrysomelidae	*Psylliodes picinus*	(Marsham, 1802)	herbivore	2.50	3.36	0	0	1	0	0	1
Coleoptera	Polyphaga	Chrysomelidae	*Sermylassa halensis*	(Linnaeus, 1767)	herbivore	6.00	33.35	9	1	0	5	1	0
Coleoptera	Polyphaga	Chrysomelidae	*Sphaeroderma rubidum*	(Graells, 1858)	herbivore	3.40	7.53	0	3	0	0	3	0
Coleoptera	Polyphaga	Chrysomelidae	*Sphaeroderma testaceum*	(Fabricius, 1775)	herbivore	3.00	5.42	1	1	0	1	1	0
Coleoptera	Polyphaga	Coccinellidae	*Coccidula rufa*	(Herbst, 1783)	predator	2.75	4.32	0	0	2	0	0	2
Coleoptera	Polyphaga	Coccinellidae	*Coccinella septempunctata*	Linnaeus, 1758	predator	6.60	42.81	20	34	41	12	13	16
Coleoptera	Polyphaga	Coccinellidae	*Coccinula quatuordecimpustulata*	(Linnaeus, 1758)	predator	3.50	8.12	0	0	3	0	0	3
Coleoptera	Polyphaga	Coccinellidae	*Cynegetis impunctata*	(Linnaeus, 1767)	herbivore	3.75	9.73	0	0	1	0	0	1
Coleoptera	Polyphaga	Coccinellidae	*Harmonia axyridis*	(Pallas, 1773)	predator	7.00	49.94	0	1	0	0	1	0
Coleoptera	Polyphaga	Coccinellidae	*Hippodamia notata*	(Laicharting, 1781)	predator	5.00	20.68	1	0	0	1	0	0
Coleoptera	Polyphaga	Coccinellidae	*Hippodamia variegata*	(Goeze, 1777)	predator	4.25	13.51	0	14	14	0	3	5
Coleoptera	Polyphaga	Coccinellidae	*Propylea quatuordecimpunctata*	(Linnaeus, 1758)	predator	4.00	11.53	11	1	9	7	1	7
Coleoptera	Polyphaga	Coccinellidae	*Scymnus femoralis*	Gyllenhal, 1827	predator	2.50	3.36	0	1	0	0	1	0

List of species sampled in 2008

								Abundance			Number of plots		
Order	Suborder	Family	Genus/Species	Author/Year	Feeding guild	mean body length [mm]	estimated biomass [g]	Swabian Alb	Hainich-Dün	Schorfheide-Chorin	Swabian Alb	Hainich-Dün	Schorfheide-Chorin
Coleoptera	Polyphaga	Coccinellidae	*Scymnus ferrugatus*	(Moll, 1785)	predator	2.75	4.32	0	0	1	0	0	1
Coleoptera	Polyphaga	Coccinellidae	*Scymnus frontalis*	(Fabricius, 1787)	predator	2.50	3.36	2	3	0	2	2	0
Coleoptera	Polyphaga	Coccinellidae	*Tytthaspis sedecimpunctata*	(Linnaeus, 1761)	predator	2.75	4.32	0	110	189	0	24	32
Coleoptera	Polyphaga	Curculionidae	*Amalus scortillum*	(Herbst, 1795)	herbivore	1.90	1.64	0	0	1	0	0	1
Coleoptera	Polyphaga	Curculionidae	*Ceutorhynchus erysimi*	(Fabricius, 1787)	herbivore	2.30	2.70	1	1	10	1	1	3
Coleoptera	Polyphaga	Curculionidae	*Ceutorhynchus floralis*	(Paykull, 1792)	herbivore	1.85	1.53	0	0	4	0	0	3
Coleoptera	Polyphaga	Curculionidae	*Chlorophanus viridis*	(Linnaeus, 1758)	herbivore	9.50	111.16	0	0	1	0	0	1
Coleoptera	Polyphaga	Curculionidae	*Eusomus ovulum*	Germar, 1824	herbivore	6.25	37.11	0	3	14	0	2	3
Coleoptera	Polyphaga	Curculionidae	*Furcipus rectirostris*	(Linnaeus, 1758)	herbivore	4.10	12.30	0	0	1	0	0	1
Coleoptera	Polyphaga	Curculionidae	*Glocianus punctiger*	(Gyllenhal, 1837)	herbivore	2.80	4.53	4	6	0	4	1	0
Coleoptera	Polyphaga	Curculionidae	*Gymnetron ictericum*	Gyllenhal, 1838	herbivore	1.90	1.64	0	1	3	0	1	2
Coleoptera	Polyphaga	Curculionidae	*Gymnetron labile*	(Herbst, 1795)	herbivore	1.95	1.75	2	7	9	2	2	4
Coleoptera	Polyphaga	Curculionidae	*Gymnetron pascuorum*	(Gyllenhal, 1813)	herbivore	1.80	1.42	1	23	5	1	8	5
Coleoptera	Polyphaga	Curculionidae	*Hypera arator*	(Linnaeus, 1758)	herbivore	5.25	23.50	2	0	0	2	0	0
Coleoptera	Polyphaga	Curculionidae	*Hypera meles*	(Fabricius, 1792)	herbivore	4.10	12.30	0	0	3	0	0	3
Coleoptera	Polyphaga	Curculionidae	*Hypera nigrirostris*	(Fabricius, 1775)	herbivore	3.40	7.53	11	23	0	8	8	0
Coleoptera	Polyphaga	Curculionidae	*Hypera postica*	(Gyllenhal, 1813)	herbivore	4.65	17.10	1	1	3	1	1	1
Coleoptera	Polyphaga	Curculionidae	*Hypera zoilus*	(Scopoli, 1763)	herbivore	7.50	59.84	2	0	1	1	0	1
Coleoptera	Polyphaga	Curculionidae	*Liophloeus tessulatus*	(Muller O.F., 1776)	herbivore	9.00	96.48	0	1	0	0	1	0
Coleoptera	Polyphaga	Curculionidae	*Marmaropus besseri*	Gyllenhal, 1837	herbivore	3.60	8.75	0	0	3	0	0	1
Coleoptera	Polyphaga	Curculionidae	*Mecinus pyraster*	(Herbst, 1795)	herbivore	3.40	7.53	2	3	0	1	3	0
Coleoptera	Polyphaga	Curculionidae	*Nedyus quadrimaculatus*	(Linnaeus, 1758)	herbivore	2.90	4.96	1	0	2	1	0	1
Coleoptera	Polyphaga	Curculionidae	*Phyllobius betulinus*	(Bechstein & Scharfenberg, 1805)	herbivore	5.00	20.68	3	0	0	2	0	0
Coleoptera	Polyphaga	Curculionidae	*Phyllobius sinuatus*	(Fabricius, 1801)	herbivore	3.50	8.12	0	1	0	0	1	0
Coleoptera	Polyphaga	Curculionidae	*Polydrusus impar*	Gozis, 1882	herbivore	6.60	42.81	1	1	0	1	1	0
Coleoptera	Polyphaga	Curculionidae	*Polydrusus sericeus*	(Mayer, 1779)	herbivore	6.25	37.11	0	1	0	0	1	0
Coleoptera	Polyphaga	Curculionidae	*Rhinocyllus conicus*	(Froehlich, 1792)	herbivore	5.50	26.55	0	0	1	0	0	1
Coleoptera	Polyphaga	Curculionidae	*Rhinoncus inconspectus*	(Herbst, 1795)	herbivore	3.35	7.24	0	0	12	0	0	5
Coleoptera	Polyphaga	Curculionidae	*Rhinoncus pericarpius*	(Linnaeus, 1758)	herbivore	2.95	5.19	4	1	4	4	1	3
Coleoptera	Polyphaga	Curculionidae	*Rhynchaenus fagi*	(Linnaeus, 1758)	herbivore	2.60	3.73	52	0	0	16	0	0
Coleoptera	Polyphaga	Curculionidae	*Sciaphilus asperatus*	(Bonsdorff, 1785)	herbivore	5.25	23.50	0	1	0	0	1	0
Coleoptera	Polyphaga	Curculionidae	*Sibinia pyrrhodactyla*	Germar, 1824	herbivore	2.45	3.19	0	0	1	0	0	1
Coleoptera	Polyphaga	Curculionidae	*Sitona gressorius*	(Fabricius, 1792)	herbivore	8.50	83.06	0	0	3	0	0	3
Coleoptera	Polyphaga	Curculionidae	*Sitona hispidulus*	(Fabricius, 1777)	herbivore	4.00	11.53	20	8	4	14	7	2
Coleoptera	Polyphaga	Curculionidae	*Sitona humeralis*	Stephens, 1831	herbivore	4.25	13.51	1	5	0	1	5	0
Coleoptera	Polyphaga	Curculionidae	*Sitona lepidus*	Gyllenhal, 1834	herbivore	5.25	23.50	52	18	15	20	9	7
Coleoptera	Polyphaga	Curculionidae	*Sitona lineatus*	(Linnaeus, 1758)	herbivore	4.00	11.53	38	41	5	9	13	4
Coleoptera	Polyphaga	Curculionidae	*Sitona sulcifrons*	(Thunberg, 1798)	herbivore	3.50	8.12	143	20	0	31	8	0
Coleoptera	Polyphaga	Curculionidae	*Sitona suturalis*	Stephens, 1831	herbivore	4.25	13.51	6	9	0	1	4	0
Coleoptera	Polyphaga	Curculionidae	*Tanymecus palliatus*	(Fabricius, 1787)	herbivore	10.00	127.15	0	0	5	0	0	3
Coleoptera	Polyphaga	Curculionidae	*Tanysphyrus lemnae*	(Paykull, 1792)	herbivore	1.60	1.04	0	0	1	0	0	1
Coleoptera	Polyphaga	Curculionidae	*Trachyphloeus alternans*	Gyllenhal, 1834	herbivore	2.50	3.36	3	0	0	2	0	0
Coleoptera	Polyphaga	Curculionidae	*Trichosirocalus horridus*	(Panzer, 1801)	herbivore	3.70	9.40	0	1	0	0	1	0
Coleoptera	Polyphaga	Curculionidae	*Trichosirocalus troglodytes*	(Fabricius, 1787)	herbivore	2.60	3.73	56	35	22	16	14	8
Coleoptera	Polyphaga	Curculionidae	*Tychius meliloti*	Stephens, 1831	herbivore	2.25	2.55	1	0	0	1	0	0
Coleoptera	Polyphaga	Curculionidae	*Tychius picirostris*	(Fabricius, 1787)	herbivore	2.15	2.27	47	51	33	11	14	13
Coleoptera	Polyphaga	Curculionidae	*Tychius stephensi*	Schonherr, 1836	herbivore	2.25	2.55	1	7	0	1	6	0
Coleoptera	Polyphaga	Elateridae	*Adrastus pallens*	(Fabricius, 1792)	herbivore	4.70	17.59	0	1	0	0	1	0
Coleoptera	Polyphaga	Elateridae	*Agriotes gallicus*	Boisduval & Lacordaire, 1835	herbivore	7.00	49.94	0	5	0	0	5	0
Coleoptera	Polyphaga	Elateridae	*Agriotes lineatus*	(Linnaeus, 1767)	herbivore	10.00	127.15	2	0	0	2	0	0
Coleoptera	Polyphaga	Elateridae	*Agriotes obscurus*	(Linnaeus, 1758)	herbivore	8.75	89.61	13	0	0	5	0	0

List of species sampled in 2008

								Abundance			Number of plots		
Order	Suborder	Family	Genus/Species	Author/Year	Feeding guild	mean body length [mm]	estimated biomass [g]	Swabian Alb	Hainich-Dün	Schorfheide-Chorin	Swabian Alb	Hainich-Dün	Schorfheide-Chorin
Coleoptera	Polyphaga	Elateridae	*Agriotes sputator*	(Linnaeus, 1758)	herbivore	7.25	54.75	5	2	2	3	2	2
Coleoptera	Polyphaga	Elateridae	*Agrypnus murina*	(Linnaeus, 1758)	predator	14.50	336.58	1	8	0	1	4	0
Coleoptera	Polyphaga	Elateridae	*Athous bicolor*	(Goeze, 1777)	herbivore	9.50	111.16	4	101	0	3	23	0
Coleoptera	Polyphaga	Elateridae	*Athous haemorrhoidalis*	(Fabricius, 1801)	herbivore	12.25	216.38	2	0	0	2	0	0
Coleoptera	Polyphaga	Elateridae	*Athous vittatus*	(Fabricius, 1792)	herbivore	10.25	135.64	0	1	0	0	1	0
Coleoptera	Polyphaga	Elateridae	*Cidnopus aeruginosus*	(Olivier, 1790)	herbivore	10.00	127.15	8	0	14	7	0	8
Coleoptera	Polyphaga	Elateridae	*Hemicrepidius hirtus*	(Herbst, 1784)	herbivore	14.50	336.58	13	2	2	5	2	1
Coleoptera	Polyphaga	Elateridae	*Hemicrepidius niger*	(Linnaeus, 1758)	herbivore	12.25	216.38	33	4	14	9	2	5
Coleoptera	Polyphaga	Elateridae	*Kibunea minutus*	(Linnaeus, 1758)	herbivore	6.75	45.40	3	1	0	2	1	0
Coleoptera	Polyphaga	Elateridae	*Mosotalesus impressus*	(Fabricius, 1792)	herbivore	14.00	307.01	0	0	1	0	0	1
Coleoptera	Polyphaga	Elateridae	*Prosternon tessellatum*	(Linnaeus, 1758)	herbivore	11.00	163.21	1	0	0	1	0	0
Coleoptera	Polyphaga	Hydrophilidae	*Cryptopleurum minutum*	(Fabricius, 1775)	herbivore	1.80	1.42	0	1	0	0	1	0
Coleoptera	Polyphaga	Hydrophilidae	*Helophorus minutus*	Fabricius, 1775	herbivore	3.15	6.16	0	7	0	0	1	0
Coleoptera	Polyphaga	Hydrophilidae	*Helophorus nubilus*	Fabricius, 1777	herbivore	3.50	8.12	1	0	0	1	0	0
Coleoptera	Polyphaga	Hydrophilidae	*Megasternum obscurum*	(Marsham, 1802)	herbivore	1.95	1.75	16	8	1	8	4	1
Coleoptera	Polyphaga	Kateritidae	*Brachypterus urticae*	(Fabricius, 1792)	herbivore	1.90	1.64	0	0	1	0	0	1
Coleoptera	Polyphaga	Lagriidae	*Lagria hirta*	(Linnaeus, 1758)	herbivore	8.50	83.06	0	1	0	0	1	0
Coleoptera	Polyphaga	Melyridae	*Dolichosoma lineare*	(Rossi, 1794)	predator	6.00	33.35	0	0	3	0	0	3
Coleoptera	Polyphaga	Mordellidae	*Mordellistena pumila*	(Gyllenhal, 1810)	herbivore	4.33	14.19	1	10	0	1	6	0
Coleoptera	Polyphaga	Nitidulidae	*Meligethes aeneus*	(Fabricius, 1775)	herbivore	2.10	2.13	1028	307	14	27	20	7
Coleoptera	Polyphaga	Nitidulidae	*Meligethes coracinus*	Sturm, 1845	herbivore	2.10	2.13	0	1	0	0	1	0
Coleoptera	Polyphaga	Nitidulidae	*Meligethes difficilis*	(Heer, 1841)	herbivore	2.25	2.55	1	0	0	1	0	0
Coleoptera	Polyphaga	Nitidulidae	*Meligethes flavimanus*	Stephens, 1830	herbivore	2.25	2.55	1	0	0	1	0	0
Coleoptera	Polyphaga	Nitidulidae	*Meligethes morosus*	Erichson, 1845	herbivore	2.05	2.00	2	0	0	2	0	0
Coleoptera	Polyphaga	Nitidulidae	*Meligethes nigrescens*	Stephens, 1830	herbivore	2.10	2.13	0	0	1	0	0	1
Coleoptera	Polyphaga	Nitidulidae	*Meligethes rotundicollis*	Brisout de Barneville, 1863	herbivore	2.00	1.87	0	0	1	0	0	1
Coleoptera	Polyphaga	Nitidulidae	*Meligethes symphyti*	(Heer, 1841)	herbivore	2.60	3.73	0	0	3	0	0	3
Coleoptera	Polyphaga	Nitidulidae	*Meligethes umbrosus*	Sturm, 1845	herbivore	2.45	3.19	1	0	0	1	0	0
Coleoptera	Polyphaga	Omalisidae	*Omalisus fontisbellaquaei*	Geoffroy, 1785	predator	7.50	59.84	5	0	0	5	0	0
Coleoptera	Polyphaga	Phalacridae	*Olibrus affinis*	(Sturm, 1807)	herbivore	2.20	2.41	0	0	2	0	0	1
Coleoptera	Polyphaga	Phalacridae	*Olibrus bimaculatus*	Kuster, 1848	herbivore	2.55	3.54	2	65	4	1	17	4
Coleoptera	Polyphaga	Phalacridae	*Olibrus flavicornis*	(Sturm, 1807)	herbivore	2.65	3.92	0	6	30	0	3	4
Coleoptera	Polyphaga	Phalacridae	*Stilbus atomarius*	(Linnaeus, 1767)	herbivore	2.00	1.87	0	0	9	0	0	6
Coleoptera	Polyphaga	Phalacridae	*Stilbus testaceus*	(Panzer, 1797)	herbivore	3.05	5.66	0	0	4	0	0	2
Coleoptera	Polyphaga	Pselaphidae	*Rybaxis longicornis*	(Leach, 1817)	predator	2.00	1.87	0	0	2	0	0	2
Coleoptera	Polyphaga	Pselaphidae	*Tychus niger*	(Paykull, 1800)	predator	1.55	0.96	0	1	0	0	1	0
Coleoptera	Polyphaga	Scarabaeidae	*Hoplia argentea*	(Poda, 1761)	herbivore	10.00	127.15	2	0	0	2	0	0
Coleoptera	Polyphaga	Scarabaeidae	*Omaloplia nigromarginata*	(Fabricius, 1775)	herbivore	6.25	37.11	2	0	0	2	0	0
Coleoptera	Polyphaga	Scarabaeidae	*Phyllopertha horticola*	(Linnaeus, 1758)	herbivore	9.75	118.98	1	0	14	1	0	5
Coleoptera	Polyphaga	Staphylinidae	*Acrotona parvula*	(Mannerheim, 1831)	predator	2.15	2.27	0	0	1	0	0	1
Coleoptera	Polyphaga	Staphylinidae	*Aleochara bipustulata*	(Linnaeus, 1761)	predator	3.25	6.69	0	1	1	0	1	1
Coleoptera	Polyphaga	Staphylinidae	*Aleochara tristis*	Gravenhorst, 1806	predator	5.25	23.50	1	0	0	1	0	0
Coleoptera	Polyphaga	Staphylinidae	*Amischa analis*	(Gravenhorst, 1802)	predator	2.10	2.13	0	1	3	0	1	2
Coleoptera	Polyphaga	Staphylinidae	*Amischa bifoveolata*	(Mannerheim, 1830)	predator	2.00	1.87	0	0	2	0	0	1
Coleoptera	Polyphaga	Staphylinidae	*Amischa decipiens*	(Sharp, 1869)	predator	2.10	2.13	0	1	0	0	1	0
Coleoptera	Polyphaga	Staphylinidae	*Amischa nigrofusca*	(Stephens, 1832)	predator	2.00	1.87	0	3	2	0	3	1
Coleoptera	Polyphaga	Staphylinidae	*Anotylus rugosus*	(Fabricius, 1775)	predator	5.00	20.68	1	0	0	1	0	0
Coleoptera	Polyphaga	Staphylinidae	*Anotylus sculpturatus*	(Gravenhorst, 1806)	predator	2.75	4.32	1	0	0	1	0	0
Coleoptera	Polyphaga	Staphylinidae	*Atheta fungi*	(Gravenhorst, 1806)	predator	2.60	3.73	0	0	13	0	0	4
Coleoptera	Polyphaga	Staphylinidae	*Atheta graminicola*	(Gravenhorst, 1806)	predator	4.25	13.51	0	0	1	0	0	1
Coleoptera	Polyphaga	Staphylinidae	*Atheta inquinula*	(Gravenhorst, 1802)	predator	1.15	0.44	0	0	1	0	0	1

List of species sampled in 2008

								Abundance			Number of plots		
Order	Suborder	Family	Genus/Species	Author/Year	Feeding guild	mean body length [mm]	estimated biomass [g]	Swabian Alb	Hainich-Dün	Schorfheide-Chorin	Swabian Alb	Hainich-Dün	Schorfheide-Chorin
Coleoptera	Polyphaga	Staphylinidae	*Atheta longicornis*	(Gravenhorst, 1802)	predator	3.25	6.69	0	1	0	0	1	0
Coleoptera	Polyphaga	Staphylinidae	*Atheta nigra*	(Kraatz, 1856)	predator	2.00	1.87	0	0	1	0	0	1
Coleoptera	Polyphaga	Staphylinidae	*Atheta putrida*	(Kraatz, 1856)	predator	3.20	6.42	1	0	0	1	0	0
Coleoptera	Polyphaga	Staphylinidae	*Cypha longicornis*	(Paykull, 1800)	predator	1.20	0.49	0	1	0	0	1	0
Coleoptera	Polyphaga	Staphylinidae	*Cypha tarsalis*	(Luze, 1902)	predator	1.20	0.49	0	0	1	0	0	1
Coleoptera	Polyphaga	Staphylinidae	*Eusphalerum luteum*	(Marsham, 1802)	herbivore	2.75	4.32	1	0	0	1	0	0
Coleoptera	Polyphaga	Staphylinidae	*Eusphalerum minutum*	(Fabricius, 1792)	herbivore	2.50	3.36	0	0	1	0	0	1
Coleoptera	Polyphaga	Staphylinidae	*Habrocerus capillaricornis*	(Gravenhorst, 1806)	predator	3.25	6.69	0	8	7	0	3	3
Coleoptera	Polyphaga	Staphylinidae	*Mycetoporus nigricollis*	(Stephens, 1835)	predator	5.25	23.50	0	1	0	0	1	0
Coleoptera	Polyphaga	Staphylinidae	*Oligota pusillima*	(Gravenhorst, 1806)	predator	1.15	0.44	0	0	2	0	0	1
Coleoptera	Polyphaga	Staphylinidae	*Oxypoda haemorrhoa*	(Mannerheim, 1830)	predator	2.35	2.86	0	0	1	0	0	1
Coleoptera	Polyphaga	Staphylinidae	*Oxytelus piceus*	(Linnaeus, 1767)	predator	4.50	15.69	0	0	1	0	0	1
Coleoptera	Polyphaga	Staphylinidae	*Paederus fuscipes*	Curtis, 1826	predator	6.75	45.40	0	0	1	0	0	1
Coleoptera	Polyphaga	Staphylinidae	*Paederus littoralis*	Gravenhorst, 1802	predator	8.00	70.86	0	8	0	0	3	0
Coleoptera	Polyphaga	Staphylinidae	*Philonthus pseudovarians*	Strand, 1941	predator	7.00	49.94	0	0	1	0	0	1
Coleoptera	Polyphaga	Staphylinidae	*Philonthus sanguinolentus*	(Gravenhorst, 1802)	predator	7.50	59.84	0	0	1	0	0	1
Coleoptera	Polyphaga	Staphylinidae	*Pycnota paradoxa*	(Mulant et Rey, 1861)	predator	2.25	2.55	0	1	0	0	1	0
Coleoptera	Polyphaga	Staphylinidae	*Quedius cinctus*	(Paykull, 1790)	predator	8.00	70.86	1	0	0	1	0	0
Coleoptera	Polyphaga	Staphylinidae	*Stenus clavicornis*	(Scopoli, 1763)	predator	5.25	23.50	1	2	1	1	2	1
Coleoptera	Polyphaga	Staphylinidae	*Stenus similis*	(Herbst, 1784)	predator	5.25	23.50	1	0	0	1	0	0
Coleoptera	Polyphaga	Staphylinidae	*Tachyporus atriceps*	Stephens, 1832	predator	2.65	3.92	0	1	0	0	1	0
Coleoptera	Polyphaga	Staphylinidae	*Tachyporus hypnorum*	(Fabricius, 1775)	predator	3.50	8.12	0	1	1	0	1	1
Coleoptera	Polyphaga	Staphylinidae	*Tachyporus nitidulus*	(Fabricius, 1781)	predator	2.50	3.36	0	4	1	0	2	1
Coleoptera	Polyphaga	Staphylinidae	*Tachyporus obtusus*	(Linnaeus, 1767)	predator	3.75	9.73	0	0	1	0	0	1
Coleoptera	Polyphaga	Staphylinidae	*Tachyporus pusillus*	Gravenhorst, 1806	predator	2.50	3.36	0	0	1	0	0	1
Coleoptera	Polyphaga	Staphylinidae	*Xantholinus linearis*	(Olivier, 1795)	predator	7.50	59.84	1	4	0	1	1	0
Hemiptera	Cicadomorpha	Aphrophoridae	*Aphrophora alni*	(Fallén, 1805)	herbivore	7.50	59.84	24	9	0	7	5	0
Hemiptera	Cicadomorpha	Aphrophoridae	*Lepyronia coleoptrata*	(Linné, 1758)	herbivore	6.75	45.40	103	2	0	14	2	0
Hemiptera	Cicadomorpha	Aphrophoridae	*Neophilaenus albipennis*	(Fabricius, 1798)	herbivore	5.50	26.55	0	1	0	0	1	0
Hemiptera	Cicadomorpha	Aphrophoridae	*Neophilaenus lineatus*	(Linné, 1758)	herbivore	5.70	29.15	1	0	0	1	0	0
Hemiptera	Cicadomorpha	Aphrophoridae	*Neophilaenus minor*	(Kirschbaum, 1868)	herbivore	4.55	16.15	8	0	0	1	0	0
Hemiptera	Cicadomorpha	Aphrophoridae	*Philaenus spumarius*	(Linné, 1758)	herbivore	6.10	34.82	404	204	8	38	23	5
Hemiptera	Cicadomorpha	Cercopidae	*Cercopis vulnerata*	Rossi, 1807	herbivore	9.70	117.39	1	0	0	1	0	0
Hemiptera	Cicadomorpha	Cicadellidae	*Adarrus multinotatus*	(Boheman, 1847)	herbivore	3.13	6.06	59	50	0	4	3	0
Hemiptera	Cicadomorpha	Cicadellidae	*Agallia brachyptera*	(Boheman, 1847)	herbivore	2.85	4.74	1	0	0	1	0	0
Hemiptera	Cicadomorpha	Cicadellidae	*Anaceratagallia ribauti*	(Ossiannilsson, 1938)	herbivore	3.05	5.66	0	10	1	0	6	1
Hemiptera	Cicadomorpha	Cicadellidae	*Anoscopus serratulae*	(Fabricius, 1775)	herbivore	3.85	10.43	1	0	0	1	0	0
Hemiptera	Cicadomorpha	Cicadellidae	*Aphrodes bicincta*	(Schrank, 1776)	herbivore	6.05	34.08	0	1	0	0	1	0
Hemiptera	Cicadomorpha	Cicadellidae	*Aphrodes diminuta*	Ribaut, 1952	herbivore	5.80	30.51	6	5	0	4	2	0
Hemiptera	Cicadomorpha	Cicadellidae	*Aphrodes makarovi*	Zachvatkin, 1948	herbivore	6.55	41.96	0	2	0	0	2	0
Hemiptera	Cicadomorpha	Cicadellidae	*Arocephalus languidus*	(Flor, 1861)	herbivore	2.55	3.54	0	1	24	0	1	5
Hemiptera	Cicadomorpha	Cicadellidae	*Arocephalus longiceps*	(Kirschbaum, 1868)	herbivore	3.80	10.08	1	0	0	1	0	0
Hemiptera	Cicadomorpha	Cicadellidae	*Arthaldeus arenarius*	Remane, 1960	herbivore	4.05	11.91	0	16	0	0	1	0
Hemiptera	Cicadomorpha	Cicadellidae	*Arthaldeus pascuellus*	(Fallén, 1826)	herbivore	3.50	8.12	1351	2601	967	39	46	46
Hemiptera	Cicadomorpha	Cicadellidae	*Arthaldeus striifrons*	(Kirschbaum, 1868)	herbivore	3.70	9.40	0	0	1	0	0	1
Hemiptera	Cicadomorpha	Cicadellidae	*Athysanus argentarius*	Metcalf, 1855	herbivore	7.25	54.75	13	5	2	7	3	2
Hemiptera	Cicadomorpha	Cicadellidae	*Balclutha punctata*	(Fabricius, 1775)	herbivore	3.85	10.43	1	0	4	1	0	4
Hemiptera	Cicadomorpha	Cicadellidae	*Chlorita paolii*	(Ossiannilsson, 1939)	herbivore	2.85	4.74	0	3	5	0	3	3
Hemiptera	Cicadomorpha	Cicadellidae	*Chlorita pusilla*	(Matsumura, 1906)	herbivore	2.15	2.27	0	0	1	0	0	1
Hemiptera	Cicadomorpha	Cicadellidae	*Cicadella viridis*	(Linné, 1758)	herbivore	7.35	56.75	0	0	6	0	0	4
Hemiptera	Cicadomorpha	Cicadellidae	*Cicadula persimilis*	(Edwards, 1920)	herbivore	4.60	16.62	220	133	111	13	19	21

List of species sampled in 2008

								Abundance			Number of plots		
Order	Suborder	Family	Genus/Species	Author/Year	Feeding guild	mean body length [mm]	estimated biomass [g]	Swabian Alb	Hainich-Dün	Schorfheide-Chorin	Swabian Alb	Hainich-Dün	Schorfheide-Chorin
Hemiptera	Cicadomorpha	Cicadellidae	*Cicadula quadrinotata*	(Fabricius, 1794)	herbivore	4.55	16.15	0	0	675	0	0	22
Hemiptera	Cicadomorpha	Cicadellidae	*Conosanus obsoletus*	(Kirschbaum, 1858)	herbivore	5.20	22.92	0	8	0	0	4	0
Hemiptera	Cicadomorpha	Cicadellidae	*Deltocephalus pulicaris*	(Fallén, 1806)	herbivore	2.75	4.32	485	825	468	32	26	33
Hemiptera	Cicadomorpha	Cicadellidae	*Diplocolenus bohemani*	(Zetterstedt, 1838)	herbivore	4.40	14.80	5	0	0	1	0	0
Hemiptera	Cicadomorpha	Cicadellidae	*Doliotettix lunulatus*	(Zetterstedt, 1838)	herbivore	4.35	14.36	4	0	0	1	0	0
Hemiptera	Cicadomorpha	Cicadellidae	*Doratura exilis*	Horváth, 1903	herbivore	3.25	6.69	76	53	0	11	10	0
Hemiptera	Cicadomorpha	Cicadellidae	*Doratura homophyla*	(Flor, 1861)	herbivore	4.00	11.53	0	0	64	0	0	6
Hemiptera	Cicadomorpha	Cicadellidae	*Elymana sulphurella*	(Zetterstedt, 1828)	herbivore	4.70	17.59	1	19	0	1	10	0
Hemiptera	Cicadomorpha	Cicadellidae	*Emelyanoviana mollicula*	(Boheman, 1845)	herbivore	3.40	7.53	21	5	0	6	3	0
Hemiptera	Cicadomorpha	Cicadellidae	*Empoasca decipiens*	Paoli, 1930	herbivore	3.60	8.75	1	0	0	1	0	0
Hemiptera	Cicadomorpha	Cicadellidae	*Empoasca pteridis*	(Dahlbom, 1850)	herbivore	3.30	6.96	0	0	3	0	0	2
Hemiptera	Cicadomorpha	Cicadellidae	*Errastunus ocellaris*	(Fallén, 1806)	herbivore	3.35	7.24	1213	646	330	29	34	35
Hemiptera	Cicadomorpha	Cicadellidae	*Erythria aureola*	(Fallén, 1806)	herbivore	2.60	3.73	10	0	0	3	0	0
Hemiptera	Cicadomorpha	Cicadellidae	*Eupteryx atropunctata*	(Goeze, 1778)	herbivore	3.60	8.75	16	3	11	8	2	5
Hemiptera	Cicadomorpha	Cicadellidae	*Eupteryx aurata*	(Linné, 1758)	herbivore	3.90	10.79	0	1	0	0	1	0
Hemiptera	Cicadomorpha	Cicadellidae	*Eupteryx cyclops*	Matsumura, 1906	herbivore	3.35	7.24	0	0	1	0	0	1
Hemiptera	Cicadomorpha	Cicadellidae	*Eupteryx notata*	Curtis, 1937	herbivore	2.40	3.02	3	28	0	3	13	0
Hemiptera	Cicadomorpha	Cicadellidae	*Eupteryx urticae*	(Fabricius, 1803)	herbivore	3.25	6.69	0	1	2	0	1	2
Hemiptera	Cicadomorpha	Cicadellidae	*Eupteryx vittata*	(Linné, 1758)	herbivore	3.40	7.53	0	1	0	0	1	0
Hemiptera	Cicadomorpha	Cicadellidae	*Euscelis incisus*	(Kirschbaum, 1858)	herbivore	3.70	9.40	314	379	84	26	47	27
Hemiptera	Cicadomorpha	Cicadellidae	*Evacanthus interruptus*	(Linné, 1758)	herbivore	6.00	33.35	2	0	0	2	0	0
Hemiptera	Cicadomorpha	Cicadellidae	*Goniagnathus brevis*	(Herrich-Schaeffer, 1835)	herbivore	5.00	20.68	3	0	0	1	0	0
Hemiptera	Cicadomorpha	Cicadellidae	*Graphocraerus ventralis*	(Fallén, 1806)	herbivore	5.50	26.55	6	2	1	3	1	1
Hemiptera	Cicadomorpha	Cicadellidae	*Grypotes puncticollis*	(Herrich-Schaeffer, 1834)	herbivore	4.50	15.69	0	0	2	0	0	2
Hemiptera	Cicadomorpha	Cicadellidae	*Hardya tenuis*	(Germar, 1821)	herbivore	3.35	7.24	395	0	0	8	0	0
Hemiptera	Cicadomorpha	Cicadellidae	*Hesium domino*	(Reuter, 1880)	herbivore	6.30	37.89	1	0	0	1	0	0
Hemiptera	Cicadomorpha	Cicadellidae	*Jassargus pseudocellaris*	(Flor, 1861)	herbivore	3.05	5.66	0	3	0	0	1	0
Hemiptera	Cicadomorpha	Cicadellidae	*Kybos smaragdula*	(Fallén, 1806)	herbivore	4.20	13.10	0	0	1	0	0	1
Hemiptera	Cicadomorpha	Cicadellidae	*Macrosteles cristatus*	(Ribaut, 1927)	herbivore	3.75	9.73	2967	4	29	22	2	3
Hemiptera	Cicadomorpha	Cicadellidae	*Macrosteles laevis*	(Ribaut, 1927)	herbivore	3.60	8.75	9574	58	4423	45	21	49
Hemiptera	Cicadomorpha	Cicadellidae	*Macrosteles sexnotatus*	(Fallén, 1806)	herbivore	3.50	8.12	17	1	131	1	1	4
Hemiptera	Cicadomorpha	Cicadellidae	*Macrosteles viridigriseus*	(Edwards, 1922)	herbivore	3.35	7.24	0	0	67	0	0	3
Hemiptera	Cicadomorpha	Cicadellidae	*Macustus grisescens*	(Zetterstedt, 1828)	herbivore	4.70	17.59	0	1	0	0	1	0
Hemiptera	Cicadomorpha	Cicadellidae	*Megophthalmus scanicus*	(Fallén, 1806)	herbivore	2.95	5.19	3	56	0	3	15	0
Hemiptera	Cicadomorpha	Cicadellidae	*Mocuellus collinus*	(Boheman, 1850)	herbivore	3.85	10.43	0	2	8	0	1	1
Hemiptera	Cicadomorpha	Cicadellidae	*Mocydia crocea*	(Herrich-Schaeffer, 1837)	herbivore	4.85	19.10	1	1	0	1	1	0
Hemiptera	Cicadomorpha	Cicadellidae	*Neoaliturus fenestratus*	(Herrich-Schaeffer, 1834)	herbivore	3.10	5.91	0	0	3	0	0	1
Hemiptera	Cicadomorpha	Cicadellidae	*Notus flavipennis*	(Zetterstedt, 1828)	herbivore	3.95	11.15	0	0	26	0	0	5
Hemiptera	Cicadomorpha	Cicadellidae	*Psammotettix alienus*	(Dahlbom, 1850)	herbivore	4.15	12.69	13	354	242	8	39	32
Hemiptera	Cicadomorpha	Cicadellidae	*Psammotettix cephalotes*	(Herrich-Schaeffer, 1834)	herbivore	3.20	6.42	376	13	0	9	1	0
Hemiptera	Cicadomorpha	Cicadellidae	*Psammotettix confinis*	(Dahlbom, 1850)	herbivore	3.60	8.75	225	216	841	13	22	33
Hemiptera	Cicadomorpha	Cicadellidae	*Psammotettix helvolus*	(Kirschbaum, 1868)	herbivore	3.20	6.42	342	242	66	15	19	4
Hemiptera	Cicadomorpha	Cicadellidae	*Psammotettix kolosvarensis*	(Matsumura, 1908)	herbivore	3.75	9.73	0	0	234	0	0	19
Hemiptera	Cicadomorpha	Cicadellidae	*Rhopalopyx preyssleri*	(Herrich-Schaeffer, 1838)	herbivore	3.75	9.73	0	1	0	0	1	0
Hemiptera	Cicadomorpha	Cicadellidae	*Rhopalopyx vitripennis*	(Flor, 1861)	herbivore	3.80	10.08	5	43	5	2	2	1
Hemiptera	Cicadomorpha	Cicadellidae	*Speudotettix subfusculus*	(Fallén, 1806)	herbivore	5.50	26.55	1	0	0	1	0	0
Hemiptera	Cicadomorpha	Cicadellidae	*Streptanus aemulans*	(Kirschbaum, 1868)	herbivore	5.05	21.23	0	6	0	0	3	0
Hemiptera	Cicadomorpha	Cicadellidae	*Streptanus sordidus*	(Zetterstedt, 1828)	herbivore	4.35	14.36	0	0	1	0	0	1
Hemiptera	Cicadomorpha	Cicadellidae	*Turrutus socialis*	(Flor, 1861)	herbivore	3.35	7.24	520	22	7	9	1	1
Hemiptera	Cicadomorpha	Cicadellidae	*Utecha trivia*	(Germar, 1821)	herbivore	3.05	5.66	1	0	0	1	0	0
Hemiptera	Cicadomorpha	Cicadellidae	*Verdanus abdominalis*	(Fabricius, 1803)	herbivore	4.15	12.69	26	50	0	5	4	0

List of species sampled in 2008

								Abundance			Number of plots		
Order	Suborder	Family	Genus/Species	Author/Year	Feeding guild	mean body length [mm]	estimated biomass [g]	Swabian Alb	Hainich-Dün	Schorfheide-Chorin	Swabian Alb	Hainich-Dün	Schorfheide-Chorin
Hemiptera	Cicadomorpha	Cicadellidae	*Zyginidia scutellaris*	(Herrich-Schaeffer, 1838)	herbivore	2.85	4.74	14	28	15	6	10	12
Hemiptera	Fulgoromorpha	Cixiidae	*Cixius nervosus*	(Linné, 1758)	herbivore	7.50	59.84	1	1	0	1	1	0
Hemiptera	Fulgoromorpha	Delphacidae	*Acanthodelphax denticauda*	(Boheman, 1847)	herbivore	3.00	5.42	0	2	0	0	1	0
Hemiptera	Fulgoromorpha	Delphacidae	*Acanthodelphax spinosa*	(Fieber, 1866)	herbivore	2.00	1.87	14	15	0	3	1	0
Hemiptera	Fulgoromorpha	Delphacidae	*Chloriona smaragdula*	(Stål, 1853)	herbivore	2.60	3.73	0	0	1	0	0	1
Hemiptera	Fulgoromorpha	Delphacidae	*Criomorphus albomarginatus*	Curtis, 1833	herbivore	3.00	5.42	0	1	0	0	1	0
Hemiptera	Fulgoromorpha	Delphacidae	*Delphacinus mesomelas*	(Boheman, 1850)	herbivore	3.00	5.42	7	7	0	4	2	0
Hemiptera	Fulgoromorpha	Delphacidae	*Dicranotropis divergens*	Kirschbaum, 1868	herbivore	2.85	4.74	1	2	0	1	1	0
Hemiptera	Fulgoromorpha	Delphacidae	*Dicranotropis hamata*	(Boheman, 1847)	herbivore	3.80	10.08	7	5	1	3	5	1
Hemiptera	Fulgoromorpha	Delphacidae	*Ditropis pteridis*	(Spinola, 1839)	herbivore	3.50	8.12	2	0	0	1	0	0
Hemiptera	Fulgoromorpha	Delphacidae	*Ditropsis flavipes*	(Signoret, 1865)	herbivore	2.35	2.86	6	0	0	2	0	0
Hemiptera	Fulgoromorpha	Delphacidae	*Eurybregma nigrolineata*	Scott, 1875	herbivore	4.20	13.10	0	0	1	0	0	1
Hemiptera	Fulgoromorpha	Delphacidae	*Hyledelphax elegantula*	(Boheman, 1847)	herbivore	3.00	5.42	2	1	0	2	1	0
Hemiptera	Fulgoromorpha	Delphacidae	*Javesella dubia*	(Kirschbaum, 1868)	herbivore	3.25	6.69	4	0	16	2	0	4
Hemiptera	Fulgoromorpha	Delphacidae	*Javesella forcipata*	(Boheman, 1847)	herbivore	3.00	5.42	4	0	0	1	0	0
Hemiptera	Fulgoromorpha	Delphacidae	*Javesella obscurella*	(Boheman, 1847)	herbivore	3.10	5.91	30	0	6	3	0	3
Hemiptera	Fulgoromorpha	Delphacidae	*Javesella pellucida*	(Fabricius, 1794)	herbivore	3.55	8.43	179	36	160	32	15	45
Hemiptera	Fulgoromorpha	Delphacidae	*Kosswigianella exigua*	(Boheman, 1847)	herbivore	2.50	3.36	21	17	0	3	1	0
Hemiptera	Fulgoromorpha	Delphacidae	*Laodelphax striatella*	(Fallén, 1826)	herbivore	3.30	6.96	21	0	50	9	0	20
Hemiptera	Fulgoromorpha	Delphacidae	*Megadelphax sordidula*	(Stål, 1853)	herbivore	4.15	12.69	70	115	20	17	13	5
Hemiptera	Fulgoromorpha	Delphacidae	*Muellerianella fairmairei*	(Perris, 1857)	herbivore	3.45	7.82	49	1	17	3	1	9
Hemiptera	Fulgoromorpha	Delphacidae	*Ribautodelphax albostriata*	(Fieber, 1866)	herbivore	3.35	7.24	29	41	4	4	10	1
Hemiptera	Fulgoromorpha	Delphacidae	*Ribautodelphax collina*	(Boheman, 1847)	herbivore	3.10	5.91	0	0	1	0	0	1
Hemiptera	Fulgoromorpha	Delphacidae	*Ribautodelphax pungens*	(Ribaut, 1953)	herbivore	3.25	6.69	9	7	0	3	1	0
Hemiptera	Fulgoromorpha	Delphacidae	*Stenocranus major*	(Kirschbaum, 1868)	herbivore	6.05	34.08	0	1	7	0	1	6
Hemiptera	Fulgoromorpha	Delphacidae	*Stenocranus minutus*	(Fabricius, 1787)	herbivore	5.15	22.35	1	8	6	1	8	3
Hemiptera	Fulgoromorpha	Delphacidae	*Stiroma affinis*	Fieber, 1866	herbivore	3.75	9.73	11	0	0	2	0	0
Hemiptera	Fulgoromorpha	Delphacidae	*Xanthodelphax flaveola*	(Flor, 1861)	herbivore	2.75	4.32	1	0	0	1	0	0
Hemiptera	Fulgoromorpha	Delphacidae	*Xanthodelphax straminea*	(Stål, 1858)	herbivore	3.00	5.42	0	13	0	0	2	0
Hemiptera	Fulgoromorpha	Tettigometridae	*Tettigometra impressopunctata*	Dufour, 1846	herbivore	4.55	16.15	7	0	0	1	0	0
Hemiptera	Heteroptera	Alydidae	*Alydus calcaratus*	(Linnaeus, 1758)	herbivore	11.00	163.21	1	1	0	1	1	0
Hemiptera	Heteroptera	Anthocoridae	*Anthocoris nemoralis*	(Fabricius, 1794)	predator	3.70	9.40	0	1	0	0	1	0
Hemiptera	Heteroptera	Anthocoridae	*Orius minutus*	(Linnaeus, 1758)	predator	2.30	2.70	0	1	0	0	1	0
Hemiptera	Heteroptera	Anthocoridae	*Orius niger*	(Wolff, 1811)	predator	2.00	1.87	0	0	3	0	0	3
Hemiptera	Heteroptera	Berytidae	*Berytinus clavipes*	(Fabricius, 1775)	herbivore	7.40	57.77	1	7	0	1	1	0
Hemiptera	Heteroptera	Berytidae	*Berytinus minor*	(Herrich-Schaeffer, 1835)	herbivore	6.00	33.35	23	1	0	3	1	0
Hemiptera	Heteroptera	Berytidae	*Berytinus signoreti*	(Fieber, 1859)	herbivore	4.90	19.62	0	1	0	0	1	0
Hemiptera	Heteroptera	Berytidae	*Gampsocoris punctipes*	(Germar, 1822)	herbivore	3.80	10.08	1	0	0	1	0	0
Hemiptera	Heteroptera	Coreidae	*Coreus marginatus*	(Linnaeus, 1758)	herbivore	13.00	252.83	1	0	3	1	0	1
Hemiptera	Heteroptera	Coreidae	*Coriomeris denticulatus*	(Scopoli, 1763)	herbivore	8.70	88.28	0	1	0	0	1	0
Hemiptera	Heteroptera	Lygaeidae	*Cymus melanocephalus*	Fieber, 1861	herbivore	3.50	8.12	0	2	0	0	1	0
Hemiptera	Heteroptera	Lygaeidae	*Drymus sylvaticus*	(Fabricius, 1775)	herbivore	4.10	12.30	0	2	0	0	1	0
Hemiptera	Heteroptera	Lygaeidae	*Ischnodemus sabuleti*	(Fallén, 1826)	herbivore	5.00	20.68	0	1	0	0	1	0
Hemiptera	Heteroptera	Lygaeidae	*Megalonotus chiragra*	(Fabricius, 1794)	herbivore	5.90	31.91	0	0	3	0	0	1
Hemiptera	Heteroptera	Lygaeidae	*Nysius ericae*	(Schilling, 1829)	herbivore	4.00	11.53	1	0	7	1	0	3
Hemiptera	Heteroptera	Lygaeidae	*Nysius senecionis*	(Schilling, 1829)	herbivore	4.30	13.93	0	0	1	0	0	1
Hemiptera	Heteroptera	Lygaeidae	*Nysius thymi*	(Wolff, 1804)	herbivore	4.00	11.53	0	0	13	0	0	2
Hemiptera	Heteroptera	Lygaeidae	*Rhyparochromus pini*	(Linnaeus, 1758)	herbivore	7.40	57.77	0	1	0	0	1	0
Hemiptera	Heteroptera	Lygaeidae	*Stygnocoris rusticus*	(Fallén, 1807)	herbivore	3.70	9.40	0	4	0	0	2	0
Hemiptera	Heteroptera	Lygaeidae	*Trapezonotus arenarius*	(Linnaeus, 1758)	herbivore	4.50	15.69	0	0	1	0	0	1
Hemiptera	Heteroptera	Miridae	*Acetropis carinata*	(Herrich-Schaeffer, 1841)	herbivore	6.40	39.49	0	52	7	0	1	5

List of species sampled in 2008

Order	Suborder	Family	Genus/Species	Author/Year	Feeding guild	mean body length [mm]	estimated biomass [g]	Abundance Swabian Alb	Abundance Hainich-Dün	Abundance Schorfheide-Chorin	Number of plots Swabian Alb	Number of plots Hainich-Dün	Number of plots Schorfheide-Chorin
Hemiptera	Heteroptera	Miridae	*Adelphocoris lineolatus*	(Goeze, 1778)	herbivore	8.40	80.52	22	36	25	6	16	8
Hemiptera	Heteroptera	Miridae	*Adelphocoris quadripunctatus*	(Fabricius, 1794)	herbivore	8.90	93.69	1	1	1	1	1	1
Hemiptera	Heteroptera	Miridae	*Adelphocoris seticornis*	(Fabricius, 1775)	herbivore	7.50	59.84	90	2	0	15	2	0
Hemiptera	Heteroptera	Miridae	*Amblytylus nasutus*	(Kirschbaum, 1856)	herbivore	4.30	13.93	199	280	185	9	14	19
Hemiptera	Heteroptera	Miridae	*Calocoris affinis*	(Herrich-Schaeffer, 1835)	herbivore	7.50	59.84	1	1	0	1	1	0
Hemiptera	Heteroptera	Miridae	*Calocoris roseomaculatus*	(De Geer, 1773)	herbivore	7.20	53.77	58	13	0	10	6	0
Hemiptera	Heteroptera	Miridae	*Capsus ater*	(Linnaeus, 1758)	herbivore	5.70	29.15	32	21	5	12	9	3
Hemiptera	Heteroptera	Miridae	*Charagochilus gyllenhalii*	(Fallén, 1807)	herbivore	3.80	10.08	0	7	0	0	5	0
Hemiptera	Heteroptera	Miridae	*Chlamydatus pulicarius*	(Fallén, 1807)	herbivore	2.50	3.36	2	5	0	2	3	0
Hemiptera	Heteroptera	Miridae	*Chlamydatus pullus*	(Reuter, 1870)	herbivore	2.30	2.70	7	1	11	2	1	4
Hemiptera	Heteroptera	Miridae	*Closterotomus norwegicus*	(Gmelin, 1790)	herbivore	7.00	49.94	0	2	0	0	2	0
Hemiptera	Heteroptera	Miridae	*Criocoris crassicornis*	(Hahn, 1834)	herbivore	3.30	6.96	0	8	0	0	3	0
Hemiptera	Heteroptera	Miridae	*Europiella artemisiae*	(Becker, 1864)	herbivore	3.00	5.42	0	0	3	0	0	2
Hemiptera	Heteroptera	Miridae	*Euryopicoris nitidus*	(Meyer-Dür, 1843)	herbivore	3.80	10.08	1	0	0	1	0	0
Hemiptera	Heteroptera	Miridae	*Hadrodemus mflavum*	(Goeze, 1778)	herbivore	7.30	55.74	1	0	0	1	0	0
Hemiptera	Heteroptera	Miridae	*Halticus apterus*	(Linnaeus, 1758)	herbivore	2.60	3.73	5	37	1	2	12	1
Hemiptera	Heteroptera	Miridae	*Hoplomachus thunbergii*	(Fallén, 1807)	herbivore	4.10	12.30	12	0	0	6	0	0
Hemiptera	Heteroptera	Miridae	*Horistus orientalis*	(Gmelin, 1790)	herbivore	6.00	33.35	38	0	0	5	0	0
Hemiptera	Heteroptera	Miridae	*Leptopterna dolabrata*	(Linnaeus, 1758)	herbivore	8.30	78.03	2307	265	619	34	22	27
Hemiptera	Heteroptera	Miridae	*Leptopterna ferrugata*	(Fallén, 1807)	herbivore	7.80	66.31	458	439	2	12	15	2
Hemiptera	Heteroptera	Miridae	*Lygus pratensis*	(Linnaeus, 1758)	herbivore	6.50	41.13	7	36	83	6	16	23
Hemiptera	Heteroptera	Miridae	*Lygus wagneri*	Remane, 1955	herbivore	6.20	36.34	2	0	0	2	0	0
Hemiptera	Heteroptera	Miridae	*Macrotylus paykullii*	(Fallén, 1807)	herbivore	3.20	6.42	0	31	0	0	4	0
Hemiptera	Heteroptera	Miridae	*Megaloceroea recticornis*	(Geoffroy, 1785)	herbivore	9.00	96.48	186	900	1	19	27	1
Hemiptera	Heteroptera	Miridae	*Megalocoleus molliculus*	(Fallén, 1807)	herbivore	4.50	15.69	4	2	0	1	1	0
Hemiptera	Heteroptera	Miridae	*Myrmecoris gracilis*	(R.F. Sahlberg, 1848)	predator	5.00	20.68	0	1	0	0	1	0
Hemiptera	Heteroptera	Miridae	*Notostira elongata*	(Geoffroy, 1785)	herbivore	7.30	55.74	164	591	298	24	44	40
Hemiptera	Heteroptera	Miridae	*Notostira erratica*	(Linnaeus, 1758)	herbivore	7.60	61.95	103	146	928	21	33	42
Hemiptera	Heteroptera	Miridae	*Orthocephalus coriaceus*	(Fabricius, 1777)	herbivore	4.80	18.58	3	4	0	2	2	0
Hemiptera	Heteroptera	Miridae	*Orthocephalus saltator*	(Hahn, 1835)	herbivore	4.50	15.69	0	2	0	0	1	0
Hemiptera	Heteroptera	Miridae	*Orthops basalis*	(A. Costa, 1853)	herbivore	4.60	16.62	10	62	19	2	16	2
Hemiptera	Heteroptera	Miridae	*Orthops campestris*	(Linnaeus, 1758)	herbivore	4.00	11.53	0	0	6	0	0	3
Hemiptera	Heteroptera	Miridae	*Orthops kalmii*	(Linnaeus, 1758)	herbivore	4.40	14.80	3	3	0	3	3	0
Hemiptera	Heteroptera	Miridae	*Pithanus maerkelii*	(Herrich-Schaeffer, 1838)	herbivore	4.50	15.69	1	3	0	1	3	0
Hemiptera	Heteroptera	Miridae	*Plagiognathus chrysanthemi*	(Wolff, 1804)	herbivore	3.60	8.75	233	930	3	23	30	1
Hemiptera	Heteroptera	Miridae	*Polymerus nigrita*	(Fallén, 1807)	herbivore	4.40	14.80	3	1	0	3	1	0
Hemiptera	Heteroptera	Miridae	*Polymerus unifasciatus*	(Fabricius, 1794)	herbivore	5.90	31.91	621	30	6	37	11	4
Hemiptera	Heteroptera	Miridae	*Stenodema calcarata*	(Fallén, 1807)	herbivore	7.30	55.74	9	107	219	2	22	31
Hemiptera	Heteroptera	Miridae	*Stenodema laevigata*	(Linnaeus, 1758)	herbivore	8.40	80.52	11	5	10	9	3	7
Hemiptera	Heteroptera	Miridae	*Stenotus binotatus*	(Fabricius, 1794)	herbivore	6.60	42.81	268	271	3	18	19	1
Hemiptera	Heteroptera	Miridae	*Strongylocoris steganoides*	(J. Sahlberg, 1875)	herbivore	3.60	8.75	4	0	0	4	0	0
Hemiptera	Heteroptera	Miridae	*Trigonotylus caelestialium*	(Kirkaldy, 1902)	herbivore	5.90	31.91	429	53	911	32	22	49
Hemiptera	Heteroptera	Miridae	*Trigonotylus ruficornis*	(Geoffroy, 1785)	herbivore	5.20	22.92	0	2	0	0	1	0
Hemiptera	Heteroptera	Miridae	*Tytthus pygmaeus*	(Zetterstedt, 1838)	predator	2.70	4.12	0	3	1	0	3	1
Hemiptera	Heteroptera	Nabidae	*Himacerus mirmicoides*	(O. Costa, 1834)	predator	7.80	66.31	2	0	0	1	0	0
Hemiptera	Heteroptera	Nabidae	*Nabis brevis*	Scholtz, 1847	predator	6.20	36.34	12	1	0	2	1	0
Hemiptera	Heteroptera	Nabidae	*Nabis ferus*	(Linnaeus, 1758)	predator	8.00	70.86	0	2	7	0	2	6
Hemiptera	Heteroptera	Nabidae	*Nabis flavomarginatus*	Scholtz, 1847	predator	8.60	85.64	8	32	0	4	7	0
Hemiptera	Heteroptera	Nabidae	*Nabis limbatus*	Dahlbom, 1851	predator	8.20	75.59	1	0	0	1	0	0
Hemiptera	Heteroptera	Nabidae	*Nabis pseudoferus*	Remane, 1949	predator	7.50	59.84	32	9	18	10	5	12
Hemiptera	Heteroptera	Nabidae	*Nabis rugosus*	(Linnaeus, 1758)	predator	6.90	48.09	44	3	1	11	3	1

List of species sampled in 2008

Order	Suborder	Family	Genus/Species	Author/Year	Feeding guild	mean body length [mm]	estimated biomass [g]	Abundance Swabian Alb	Abundance Hainich-Dün	Abundance Schorfheide-Chorin	Number of plots Swabian Alb	Number of plots Hainich-Dün	Number of plots Schorfheide-Chorin
Hemiptera	Heteroptera	Pentatomidae	*Aelia acuminata*	(Linnaeus, 1758)	herbivore	8.50	83.06	0	15	31	0	7	13
Hemiptera	Heteroptera	Pentatomidae	*Carpocoris fuscispinus*	(Boheman, 1850)	herbivore	12.20	214.07	0	1	1	0	1	1
Hemiptera	Heteroptera	Pentatomidae	*Carpocoris purpureipennis*	(De Geer, 1773)	herbivore	12.00	205.00	9	2	2	8	2	1
Hemiptera	Heteroptera	Pentatomidae	*Dolycoris baccarum*	(Linnaeus, 1758)	herbivore	11.00	163.21	8	4	9	6	4	8
Hemiptera	Heteroptera	Pentatomidae	*Eurydema oleracea*	(Linnaeus, 1758)	herbivore	6.50	41.13	0	2	1	0	2	1
Hemiptera	Heteroptera	Pentatomidae	*Peribalus strictus*	(Fabricius, 1803)	herbivore	9.50	111.16	1	0	0	1	0	0
Hemiptera	Heteroptera	Pentatomidae	*Piezodorus lituratus*	(Fabricius, 1794)	herbivore	11.00	163.21	0	0	1	0	0	1
Hemiptera	Heteroptera	Pentatomidae	*Podops inunctus*	(Fabricius, 1775)	herbivore	5.70	29.15	0	1	0	0	1	0
Hemiptera	Heteroptera	Pentatomidae	*Sciocoris cursitans*	(Fabricius, 1794)	herbivore	5.20	22.92	1	0	0	1	0	0
Hemiptera	Heteroptera	Plataspididae	*Coptosoma scutellatum*	(Geoffroy, 1785)	herbivore	4.10	12.30	0	5	0	0	4	0
Hemiptera	Heteroptera	Rhopalidae	*Corizus hyoscyami*	(Linnaeus, 1758)	herbivore	9.40	108.12	0	1	0	0	1	0
Hemiptera	Heteroptera	Rhopalidae	*Myrmus miriformis*	(Fallén, 1807)	herbivore	7.70	64.11	2	1	2	1	1	2
Hemiptera	Heteroptera	Rhopalidae	*Rhopalus conspersus*	(Fieber, 1837)	herbivore	6.50	41.13	5	0	0	2	0	0
Hemiptera	Heteroptera	Rhopalidae	*Rhopalus parumpunctatus*	Schilling, 1829	herbivore	6.80	46.29	1	0	9	1	0	2
Hemiptera	Heteroptera	Rhopalidae	*Rhopalus subrufus*	(Gmelin, 1790)	herbivore	7.10	51.83	0	1	0	0	1	0
Hemiptera	Heteroptera	Rhopalidae	*Stictopleurus abutilon*	(Rossi, 1790)	herbivore	7.80	66.31	0	0	13	0	0	3
Hemiptera	Heteroptera	Rhopalidae	*Stictopleurus crassicornis*	(Linnaeus, 1758)	herbivore	7.40	57.77	2	0	0	1	0	0
Hemiptera	Heteroptera	Rhopalidae	*Stictopleurus punctatonervosus*	(Goeze, 1778)	herbivore	7.50	59.84	0	0	3	0	0	3
Hemiptera	Heteroptera	Scutelleridae	*Eurygaster maura*	(Linnaeus, 1758)	herbivore	9.70	117.39	3	5	3	2	4	2
Hemiptera	Heteroptera	Scutelleridae	*Eurygaster testudinaria*	(Geoffroy, 1785)	herbivore	9.20	102.19	6	0	1	6	0	1
Hemiptera	Heteroptera	Tingidae	*Acalypta nigrina*	(Fallén, 1807)	herbivore	2.60	3.73	1	0	0	1	0	0
Hemiptera	Heteroptera	Tingidae	*Acalypta parvula*	(Fallén, 1807)	herbivore	2.00	1.87	1	0	1	1	0	1
Hemiptera	Heteroptera	Tingidae	*Acalypta platycheila*	(Fieber, 1844)	herbivore	2.60	3.73	0	1	0	0	1	0
Hemiptera	Heteroptera	Tingidae	*Dictyla humuli*	(Fabricius, 1794)	herbivore	3.40	7.53	0	0	1	0	0	1
Hemiptera	Heteroptera	Tingidae	*Kalama tricornis*	(Schrank, 1801)	herbivore	3.10	5.91	1	6	7	1	5	2
Hemiptera	Heteroptera	Tingidae	*Lasiacantha capucina*	(Germar, 1837)	herbivore	2.70	4.12	5	0	0	2	0	0
Hemiptera	Heteroptera	Tingidae	*Oncochila simplex*	(Herrich-Schaeffer, 1830)	herbivore	3.20	6.42	3	0	0	2	0	0
Hemiptera	Heteroptera	Tingidae	*Tingis cardui*	(Linnaeus, 1758)	herbivore	3.40	7.53	1	0	0	1	0	0
Orthoptera	Caelifera	Acrididae	*Chorthippus albomarginatus*	(De Geer, 1773)	herbivore	17.00	510.59	0	29	35	0	15	19
Orthoptera	Caelifera	Acrididae	*Chorthippus apricarius*	(Linnaeus, 1758)	herbivore	18.00	593.08	0	0	6	0	0	3
Orthoptera	Caelifera	Acrididae	*Chorthippus biguttulus*	(Linnaeus, 1758)	herbivore	18.00	593.08	36	41	29	15	17	9
Orthoptera	Caelifera	Acrididae	*Chorthippus dorsatus*	(Zetterstedt, 1821)	herbivore	20.00	781.62	0	11	68	0	7	23
Orthoptera	Caelifera	Acrididae	*Chorthippus montanus*	(Charpentier, 1825)	herbivore	18.00	593.08	0	0	18	0	0	8
Orthoptera	Caelifera	Acrididae	*Chorthippus parallelus*	(Zetterstedt, 1821)	herbivore	18.00	593.08	92	95	21	25	28	11
Orthoptera	Caelifera	Acrididae	*Euthystira brachyptera*	(Ocskay, 1826)	herbivore	20.00	781.62	16	0	0	4	0	0
Orthoptera	Caelifera	Acrididae	*Omocestus viridulus*	(Linnaeus, 1758)	herbivore	18.00	593.08	11	3	0	6	2	0
Orthoptera	Caelifera	Acrididae	*Stenobothrus lineatus*	(Panzer, 1796)	herbivore	20.00	781.62	9	1	0	4	1	0
Orthoptera	Caelifera	Acrididae	*Stethophyma grossum*	(Linnaeus, 1758)	herbivore	26.00	1554.28	0	0	4	0	0	4
Orthoptera	Caelifera	Tetrigidae	*Tetrix bipunctata*	(Linnaeus, 1758)	herbivore	10.00	127.15	8	0	0	3	0	0
Orthoptera	Caelifera	Tetrigidae	*Tetrix subulata*	(Linnaeus, 1758)	herbivore	10.00	127.15	0	0	14	0	0	11
Orthoptera	Caelifera	Tetrigidae	*Tetrix tenuicornis*	(Sahlberg, 1893)	herbivore	9.00	96.48	4	1	0	1	1	0
Orthoptera	Ensifera	Conocephalidae	*Conocephalus dorsalis*	(Latreille, 1804)	predator	14.00	307.01	0	0	5	0	0	5
Orthoptera	Ensifera	Phaneropteridae	*Isophya kraussii*	Brunner von Wattenwyl, 1878	herbivore	21.00	888.21	1	0	0	1	0	0
Orthoptera	Ensifera	Tettigoniidae	*Metrioptera roeselii*	(Hagenbach, 1822)	herbivore	16.00	435.61	3	1	1	2	1	1
