# Supplementary material for: Resource-Mediated Indirect Effects of Grassland Management on Arthropod Diversity
Source: PLoS One. 2014 Sep 4;9(9):e107033. doi: 10.1371/journal.pone.0107033 (PMC4154770; doi:10.1371/journal.pone.0107033)
Supplement: Appendix S6 — List of arthropod species sampled in 2009. (PDF) [file pone.0107033.s010.pdf]

List of species sampled in 2009

								Abundance			Number of plots		
Order	Suborder	Family	Genus/Species	Author/Year	Feeding guild	mean body length [mm]	estimated biomass [g]	Swabian Alb	Hainich-Dün	Schorfheide-Chorin	Swabian Alb	Hainich-Dün	Schorfheide-Chorin
Araneae	Labodignatha	Araneidae	*Aculepeira ceropegia*	Walckenaer, 1802	predator	11.00	163.21	10	3	0	8	3	0
Araneae	Labodignatha	Araneidae	*Araneus diadematus*	Clerck, 1757	predator	11.88	199.67	1	0	1	1	0	1
Araneae	Labodignatha	Araneidae	*Araneus quadratus*	Clerck, 1757	predator	12.25	216.38	2	2	18	2	1	3
Araneae	Labodignatha	Araneidae	*Araniella cucurbitina*	(Clerck, 1757)	predator	5.50	26.55	2	1	0	2	1	0
Araneae	Labodignatha	Araneidae	*Argiope bruennichi*	(Scopoli, 1772)	predator	11.78	195.30	0	1	0	0	1	0
Araneae	Labodignatha	Araneidae	*Hypsosinga albovittata*	(Westring, 1851)	predator	4.15	12.69	1	0	0	1	0	0
Araneae	Labodignatha	Araneidae	*Mangora acalypha*	(Walckenaer, 1802)	predator	4.50	15.69	10	14	6	10	11	4
Araneae	Labodignatha	Linyphiidae	*Araeoncus humilis*	(Blackwall, 1841)	predator	1.60	1.04	1	1	2	1	1	2
Araneae	Labodignatha	Linyphiidae	*Bathyphantes gracilis*	(Blackwall, 1841)	predator	1.98	1.83	0	1	7	0	1	7
Araneae	Labodignatha	Linyphiidae	*Bathyphantes parvulus*	(Westring, 1851)	predator	2.15	2.27	0	2	0	0	2	0
Araneae	Labodignatha	Linyphiidae	*Ceratinella scabrosa*	(O. P.-Cambridge, 1871)	predator	1.88	1.59	0	1	0	0	1	0
Araneae	Labodignatha	Linyphiidae	*Collinsia inerrans*	(O. P.-Cambridge, 1885)	predator	2.25	2.55	0	1	0	0	1	0
Araneae	Labodignatha	Linyphiidae	*Dicymbium nigrum brevisetosum*	(Blackwall, 1834)	predator	2.25	2.55	15	0	0	5	0	0
Araneae	Labodignatha	Linyphiidae	*Dismodicus elevatus*	(C.L. Koch, 1838)	predator	2.48	3.29	0	1	0	0	1	0
Araneae	Labodignatha	Linyphiidae	*Entelecara acuminata*	(Wider, 1834)	predator	2.05	2.00	1	2	0	1	2	0
Araneae	Labodignatha	Linyphiidae	*Entelecara congenera*	(O. P.-Cambridge, 1879)	predator	1.94	1.73	5	1	0	5	1	0
Araneae	Labodignatha	Linyphiidae	*Entelecara flavipes*	(Blackwall, 1834)	predator	1.58	1.01	1	1	0	1	1	0
Araneae	Labodignatha	Linyphiidae	*Erigone atra*	Blackwall, 1833	predator	2.25	2.55	4	3	10	4	3	9
Araneae	Labodignatha	Linyphiidae	*Erigone dentipalpis*	(Wider, 1834)	predator	2.33	2.80	2	4	3	2	4	3
Araneae	Labodignatha	Linyphiidae	*Linyphia triangularis*	(Clerck, 1757)	predator	6.00	33.35	2	0	0	2	0	0
Araneae	Labodignatha	Linyphiidae	*Maso sundevalli*	(Westring, 1851)	predator	1.58	1.01	1	0	0	1	0	0
Araneae	Labodignatha	Linyphiidae	*Meioneta rurestris*	(C.L. Koch, 1836)	predator	6.23	36.80	3	2	7	3	2	7
Araneae	Labodignatha	Linyphiidae	*Mermessus trilobatus*	(Emerton, 1882)	predator	1.85	1.53	4	3	0	2	3	0
Araneae	Labodignatha	Linyphiidae	*Microlinyphia pusilla*	(Sundevall, 1830)	predator	3.88	10.64	2	0	4	2	0	4
Araneae	Labodignatha	Linyphiidae	*Oedothorax apicatus*	(Blackwall, 1850)	predator	2.55	3.54	1	8	3	1	6	3
Araneae	Labodignatha	Linyphiidae	*Oedothorax fuscus*	(Blackwall, 1834)	predator	2.35	2.86	0	0	3	0	0	2
Araneae	Labodignatha	Linyphiidae	*Oedothorax retusus*	(Westring, 1851)	predator	2.48	3.29	0	0	1	0	0	1
Araneae	Labodignatha	Linyphiidae	*Pelecopsis parallela*	(Wider, 1834)	predator	1.45	0.81	0	0	1	0	0	1
Araneae	Labodignatha	Linyphiidae	*Porrhomma microphthalmum*	(O. P.-Cambridge, 1871)	predator	1.85	1.53	0	5	0	0	4	0
Araneae	Labodignatha	Linyphiidae	*Tenuiphantes flavipes*	(Blackwall, 1854)	predator	2.15	2.27	0	1	0	0	1	0
Araneae	Labodignatha	Linyphiidae	*Tenuiphantes tenuis*	(Blackwall, 1852)	predator	2.88	4.87	6	13	6	5	11	4
Araneae	Labodignatha	Linyphiidae	*Tiso vagans*	(Blackwall, 1834)	predator	2.03	1.95	3	1	8	2	1	5
Araneae	Labodignatha	Linyphiidae	*Trematocephalus cristatus*	(Wider, 1834)	predator	2.30	2.70	1	0	0	1	0	0
Araneae	Labodignatha	Lycosidae	*Alopecosa cuneata*	(Clerck, 1757)	predator	7.25	54.75	1	0	0	1	0	0
Araneae	Labodignatha	Lycosidae	*Pardosa amentata*	(Clerck, 1757)	predator	6.25	37.11	1	0	1	1	0	1
Araneae	Labodignatha	Lycosidae	*Pardosa palustris*	(Linnaeus, 1758)	predator	6.00	33.35	1	0	0	1	0	0
Araneae	Labodignatha	Lycosidae	*Pardosa prativaga*	L. Koch, 1870	predator	6.10	34.82	0	0	2	0	0	1
Araneae	Labodignatha	Lycosidae	*Pardosa pullata*	(Clerck, 1757)	predator	4.75	18.08	0	1	0	0	1	0
Araneae	Labodignatha	Lycosidae	*Trochosa ruricola*	(De Geer, 1778)	predator	9.63	115.19	0	1	0	0	1	0
Araneae	Labodignatha	Philodromidae	*Philodromus albidus*	Kulczynski, 1911	predator	4.12	12.45	0	1	2	0	1	2
Araneae	Labodignatha	Philodromidae	*Philodromus aureolus*	(Clerck, 1757)	predator	5.63	28.22	3	1	0	3	1	0
Araneae	Labodignatha	Philodromidae	*Philodromus collinus*	C.L. Koch, 1835	predator	4.88	19.41	2	0	0	2	0	0
Araneae	Labodignatha	Philodromidae	*Tibellus oblongus*	(Franganillo, 1926)	predator	7.63	62.59	0	3	0	0	3	0
Araneae	Labodignatha	Tetragnathidae	*Metellina segmentata*	(Clerck, 1757)	predator	7.50	59.84	0	2	0	0	2	0
Araneae	Labodignatha	Tetragnathidae	*Pachygnatha degeeri*	Sundevall, 1830	predator	3.55	8.43	6	0	0	5	0	0
Araneae	Labodignatha	Tetragnathidae	*Tetragnatha extensa*	(Linnaeus, 1758)	predator	8.05	72.03	2	0	5	2	0	2
Araneae	Labodignatha	Tetragnathidae	*Tetragnatha pinicola*	L. Koch, 1870	predator	5.13	22.12	3	0	0	3	0	0
Araneae	Labodignatha	Theridiidae	*Cryptachaea riparia*	(Blackwall, 1834)	predator	3.50	8.12	5	0	0	5	0	0
Araneae	Labodignatha	Theridiidae	*Lasaeola tristis*	(Hahn, 1833)	predator	3.30	6.96	2	0	0	2	0	0
Araneae	Labodignatha	Theridiidae	*Neottiura bimaculata*	(Linnaeus, 1767)	predator	2.50	3.36	3	5	1	3	5	1
Araneae	Labodignatha	Theridiidae	*Paidiscura pallens*	(Blackwall, 1834)	predator	1.58	1.01	2	4	0	2	4	0
Araneae	Labodignatha	Theridiidae	*Parasteatoda simulans*	(Thorell, 1875)	predator	3.53	8.31	1	0	0	1	0	0
Araneae	Labodignatha	Theridiidae	*Phylloneta impressa*	(L. Koch, 1881)	predator	4.25	13.51	32	22	18	24	17	13
Araneae	Labodignatha	Theridiidae	*Phylloneta sisyphia*	(Clerck, 1757)	predator	3.68	9.26	1	0	0	1	0	0
Araneae	Labodignatha	Theridiidae	*Platnickina tincta*	(Walckenaer, 1802)	predator	3.30	6.96	2	4	0	2	4	0
Araneae	Labodignatha	Theridiidae	*Robertus arundineti*	(O. P.-Cambridge, 1871)	predator	2.31	2.74	0	1	0	0	1	0
Araneae	Labodignatha	Theridiidae	*Robertus neglectus*	(O. P.-Cambridge, 1871)	predator	2.13	2.21	0	1	0	0	1	0
Araneae	Labodignatha	Theridiidae	*Simitidion simile*	(C.L. Koch, 1836)	predator	2.60	3.73	1	0	0	1	0	0
Araneae	Labodignatha	Theridiidae	*Theridion boesenbergi*	Strand, 1904	predator	2.13	2.21	1	0	0	1	0	0
Araneae	Labodignatha	Theridiidae	*Theridion pinastri*	L. Koch, 1872	predator	3.13	6.06	1	0	4	1	0	4

List of species sampled in 2009

								Abundance			Number of plots		
**Order**	**Suborder**	**Family**	**Genus/Species**	**Author/Year**	**Feeding guild**	**mean body length [mm]**	**estimated biomass [g]**	**Swabian Alb**	**Hainich-Dün**	**Schorfheide-Chorin**	**Swabian Alb**	**Hainich-Dün**	**Schorfheide-Chorin**
Araneae	Labodignatha	Thomisidae	*Diaea dorsata*	(Fabricius, 1777)	predator	4.83	18.89	1	0	0	1	0	0
Araneae	Labodignatha	Thomisidae	*Ebrechtella tricuspidata*	(Fabricius, 1775)	predator	4.50	15.69	0	0	2	0	0	2
Araneae	Labodignatha	Thomisidae	*Misumena vatia*	(Clerck, 1757)	predator	6.63	43.32	2	1	0	2	1	0
Araneae	Labodignatha	Thomisidae	*Xysticus bifasciatus*	C.L. Koch, 1837	predator	7.03	50.50	1	0	0	1	0	0
Araneae	Labodignatha	Thomisidae	*Xysticus cristatus*	(Clerck, 1757)	predator	5.58	27.57	6	4	4	6	4	1
Araneae	Labodignatha	Thomisidae	*Xysticus lanio*	C.L. Koch, 1835	predator	6.13	35.27	1	0	0	1	0	0
Araneae	Labodignatha	Thomisidae	*Xysticus striatipes*	L. Koch, 1870	predator	6.30	37.89	0	0	2	0	0	1
Araneae	Labodignatha	Thomisidae	*Xysticus ulmi*	(Hahn, 1832)	predator	5.08	21.56	2	1	0	2	1	0
Coleoptera	Adephaga	Carabidae	*Amara aulica*	(Panzer, 1797)	herbivore	12.75	240.29	1	1	0	1	1	0
Coleoptera	Adephaga	Carabidae	*Amara familiaris*	(Duftschmid, 1812)	herbivore	6.50	41.13	3	2	0	1	2	0
Coleoptera	Adephaga	Carabidae	*Amara plebeja*	(Gyllenhal, 1810)	herbivore	7.00	49.94	1	0	0	1	0	0
Coleoptera	Adephaga	Carabidae	*Ophonus diffinis*	Dejean, 1829	herbivore	11.25	173.11	0	1	0	0	1	0
Coleoptera	Adephaga	Carabidae	*Poecilus cupreus*	(Linnaeus, 1758)	predator	11.00	163.21	0	0	1	0	0	1
Coleoptera	Adephaga	Carabidae	*Poecilus versicolor*	(Sturm, 1824)	predator	9.75	118.98	0	0	2	0	0	2
Coleoptera	Polyphaga	Alleculidae	*Cteniopus flavus*	(Scopoli, 1763)	herbivore	8.00	70.86	0	0	1	0	0	1
Coleoptera	Polyphaga	Apionidae	*Acanephodus onopordi*	(Kirby, 1808)	herbivore	2.65	3.92	0	1	2	0	1	2
Coleoptera	Polyphaga	Apionidae	*Apion frumentarium*	Linnaeus, 1758	herbivore	3.90	10.79	0	0	1	0	0	1
Coleoptera	Polyphaga	Apionidae	*Apion haematodes*	Kirby, 1808	herbivore	2.65	3.92	2	0	0	2	0	0
Coleoptera	Polyphaga	Apionidae	*Catapion pubescens*	(Kirby, 1811)	herbivore	1.95	1.75	0	1	1	0	1	1
Coleoptera	Polyphaga	Apionidae	*Catapion seniculus*	(Kirby, 1808)	herbivore	1.80	1.42	1	2	0	1	2	0
Coleoptera	Polyphaga	Apionidae	*Cyanapion spencii*	(Kirby, 1808)	herbivore	2.20	2.41	0	1	0	0	1	0
Coleoptera	Polyphaga	Apionidae	*Diplapion confluens*	(Kirby, 1808)	herbivore	2.05	2.00	0	1	0	0	1	0
Coleoptera	Polyphaga	Apionidae	*Eutrichapion ervi*	(Kirby, 1808)	herbivore	2.20	2.41	1	0	0	1	0	0
Coleoptera	Polyphaga	Apionidae	*Eutrichapion punctigerum*	(Paykull, 1792)	herbivore	2.70	4.12	0	1	0	0	1	0
Coleoptera	Polyphaga	Apionidae	*Holotrichapion ononis*	(Kirby, 1808)	herbivore	2.05	2.00	0	2	0	0	1	0
Coleoptera	Polyphaga	Apionidae	*Holotrichapion pisi*	(Fabricius, 1801)	herbivore	2.55	3.54	0	2	3	0	2	2
Coleoptera	Polyphaga	Apionidae	*Ischnopterapion loti*	(Kirby, 1808)	herbivore	2.25	2.55	5	3	0	4	3	0
Coleoptera	Polyphaga	Apionidae	*Ischnopterapion virens*	(Herbst, 1797)	herbivore	2.20	2.41	11	3	24	11	3	13
Coleoptera	Polyphaga	Apionidae	*Nanophyes marmoratus*	(Goeze, 1777)	herbivore	1.75	1.32	0	0	1	0	0	1
Coleoptera	Polyphaga	Apionidae	*Perapion curtirostre*	(Germar, 1817)	herbivore	2.15	2.27	1	0	3	1	0	3
Coleoptera	Polyphaga	Apionidae	*Perapion violaceum*	(Kirby, 1808)	herbivore	3.05	5.66	3	0	2	3	0	2
Coleoptera	Polyphaga	Apionidae	*Protapion apricans*	(Herbst, 1797)	herbivore	2.45	3.19	25	21	2	15	12	2
Coleoptera	Polyphaga	Apionidae	*Protapion assimile*	Kirby, 1808	herbivore	2.05	2.00	22	4	0	8	3	0
Coleoptera	Polyphaga	Apionidae	*Protapion filirostre*	(Kirby, 1808)	herbivore	1.75	1.32	0	0	1	0	0	1
Coleoptera	Polyphaga	Apionidae	*Protapion fulvipes*	(Geoffroy, 1785)	herbivore	2.00	1.87	28	25	10	14	9	9
Coleoptera	Polyphaga	Apionidae	*Protapion nigritarse*	(Kirby, 1808)	herbivore	1.75	1.32	0	7	2	0	4	1
Coleoptera	Polyphaga	Apionidae	*Protapion trifolii*	(Linnaeus, 1768)	herbivore	1.90	1.64	0	20	1	0	10	1
Coleoptera	Polyphaga	Apionidae	*Squamapion atomarium*	(Kirby, 1808)	herbivore	1.40	0.74	1	0	0	1	0	0
Coleoptera	Polyphaga	Apionidae	*Taeniapion urticarium*	(Herbst, 1784)	herbivore	2.10	2.13	0	0	2	0	0	1
Coleoptera	Polyphaga	Bruchidae	*Bruchus luteicornis*	Illiger, 1794	herbivore	2.15	2.27	7	1	0	5	1	0
Coleoptera	Polyphaga	Cantharidae	*Cantharis fulvicollis*	Fabricius, 1792	predator	6.25	37.11	0	145	47	0	24	10
Coleoptera	Polyphaga	Cantharidae	*Cantharis fusca*	Linnaeus, 1758	predator	13.00	252.83	13	0	1	9	0	1
Coleoptera	Polyphaga	Cantharidae	*Cantharis livida*	Linnaeus, 1758	predator	11.25	173.11	1	0	0	1	0	0
Coleoptera	Polyphaga	Cantharidae	*Cantharis paludosa*	Fallén, 1807	predator	5.50	26.55	0	1	0	0	1	0
Coleoptera	Polyphaga	Cantharidae	*Cantharis rufa*	Linnaeus, 1758	predator	9.75	118.98	1	4	1	1	3	1
Coleoptera	Polyphaga	Cantharidae	*Malthinus punctatus*	(Fourcroy, 1785)	predator	5.50	26.55	1	0	0	1	0	0
Coleoptera	Polyphaga	Cantharidae	*Malthodes lobatus*	Kiesenwetter, 1852	predator	1.00	0.31	0	52	0	0	9	0
Coleoptera	Polyphaga	Cantharidae	*Malthodes pumilus*	(Brebisson , 1835)	predator	1.40	0.74	0	13	1	0	6	1
Coleoptera	Polyphaga	Cantharidae	*Rhagonycha fulva*	(Scopoli, 1763)	predator	8.50	83.06	0	0	5	0	0	3
Coleoptera	Polyphaga	Cantharidae	*Rhagonycha limbata*	Thomson, 1864	predator	5.50	26.55	11	5	0	6	5	0
Coleoptera	Polyphaga	Cantharidae	*Rhagonycha lutea*	(Muller O.F., 1764)	predator	7.50	59.84	1	0	0	1	0	0
Coleoptera	Polyphaga	Cholevidae	*Nargus velox*	(Spence, 1815)	predator	2.95	5.19	0	0	1	0	0	1
Coleoptera	Polyphaga	Cholevidae	*Ptomaphagus sericatus*	(Chaudoir, 1845)	predator	2.40	3.02	1	0	0	1	0	0
Coleoptera	Polyphaga	Cholevidae	*Sciodrepoides watsoni*	(Spence, 1815)	predator	3.00	5.42	0	0	1	0	0	1
Coleoptera	Polyphaga	Chrysomelidae	*Altica oleracea*	(Linnaeus, 1758)	herbivore	3.00	5.42	6	0	4	5	0	3
Coleoptera	Polyphaga	Chrysomelidae	*Aphthona cyparissiae*	(Koch, 1803)	herbivore	4.00	11.53	4	0	0	3	0	0
Coleoptera	Polyphaga	Chrysomelidae	*Aphthona herbigrada*	(Curtis, 1837)	herbivore	1.90	1.64	1	0	0	1	0	0
Coleoptera	Polyphaga	Chrysomelidae	*Aphthona pallida*	(Bach, 1856)	herbivore	1.65	1.13	3	2	4	3	2	3
Coleoptera	Polyphaga	Chrysomelidae	*Aphthona pygmaea*	(Kutschera, 1861)	herbivore	3.00	5.42	1	0	0	1	0	0
Coleoptera	Polyphaga	Chrysomelidae	*Asiorestia ferruginea*	(Scopoli, 1763)	herbivore	3.25	6.69	51	3	16	11	3	9

List of species sampled in 2009

								Abundance			Number of plots		
Order	Suborder	Family	Genus/Species	Author/Year	Feeding guild	mean body length [mm]	estimated biomass [g]	Swabian Alb	Hainich-Dün	Schorfheide-Chorin	Swabian Alb	Hainich-Dün	Schorfheide-Chorin
Coleoptera	Polyphaga	Chrysomelidae	*Asiorestia transversa*	(Marsham, 1802)	herbivore	4.25	13.51	0	0	12	0	0	6
Coleoptera	Polyphaga	Chrysomelidae	*Cassida stigmatica*	Suffrian, 1844	herbivore	5.75	29.83	0	0	2	0	0	2
Coleoptera	Polyphaga	Chrysomelidae	*Cassida vibex*	Linnaeus, 1767	herbivore	6.25	37.11	0	0	3	0	0	2
Coleoptera	Polyphaga	Chrysomelidae	*Chaetocnema concinna*	(Marsham, 1802)	herbivore	2.10	2.13	1	2	13	1	2	4
Coleoptera	Polyphaga	Chrysomelidae	*Chaetocnema hortensis*	(Geoffroy, 1785)	herbivore	1.90	1.64	0	1	6	0	1	6
Coleoptera	Polyphaga	Chrysomelidae	*Chaetocnema laevicollis*	(Thomson, 1866)	herbivore	2.00	1.87	1	0	1	1	0	1
Coleoptera	Polyphaga	Chrysomelidae	*Chrysolina oricalcia*	(Muller O.F., 1776)	herbivore	7.50	59.84	0	1	0	0	1	0
Coleoptera	Polyphaga	Chrysomelidae	*Clytra laeviuscula*	Ratzeburg, 1837	herbivore	9.00	96.48	0	1	0	0	1	0
Coleoptera	Polyphaga	Chrysomelidae	*Cryptocephalus aureolus*	Suffrian, 1847	herbivore	6.00	33.35	0	1	0	0	1	0
Coleoptera	Polyphaga	Chrysomelidae	*Cryptocephalus fulvus*	Goeze, 1777	herbivore	2.50	3.36	0	2	7	0	1	2
Coleoptera	Polyphaga	Chrysomelidae	*Cryptocephalus hypochaeridis*	(Linnaeus, 1758)	herbivore	5.00	20.68	7	7	0	5	4	0
Coleoptera	Polyphaga	Chrysomelidae	*Cryptocephalus labiatus*	(Linnaeus, 1761)	herbivore	2.40	3.02	0	1	0	0	1	0
Coleoptera	Polyphaga	Chrysomelidae	*Cryptocephalus moraei*	(Linnaeus, 1758)	herbivore	4.00	11.53	1	1	0	1	1	0
Coleoptera	Polyphaga	Chrysomelidae	*Cryptocephalus sericeus*	(Linnaeus, 1758)	herbivore	6.10	34.82	1	3	0	1	2	0
Coleoptera	Polyphaga	Chrysomelidae	*Cryptocephalus vittatus*	Fabricius, 1775	herbivore	3.75	9.73	6	0	0	4	0	0
Coleoptera	Polyphaga	Chrysomelidae	*Derocrepis rufipes*	(Linnaeus, 1758)	herbivore	3.30	6.96	7	0	0	1	0	0
Coleoptera	Polyphaga	Chrysomelidae	*Dibolia cryptocephala*	(Koch, 1803)	herbivore	1.90	1.64	4	0	0	3	0	0
Coleoptera	Polyphaga	Chrysomelidae	*Galeruca tanaceti*	(Linnaeus, 1758)	herbivore	8.00	70.86	1	0	0	1	0	0
Coleoptera	Polyphaga	Chrysomelidae	*Gastrophysa polygoni*	(Linnaeus, 1758)	herbivore	4.50	15.69	0	0	1	0	0	1
Coleoptera	Polyphaga	Chrysomelidae	*Gastrophysa viridula*	(De Geer, 1775)	herbivore	5.00	20.68	2	0	8	1	0	2
Coleoptera	Polyphaga	Chrysomelidae	*Hispa atra*	Linnaeus, 1767	herbivore	3.50	8.12	0	0	4	0	0	1
Coleoptera	Polyphaga	Chrysomelidae	*Labidostomis longimana*	(Linnaeus, 1761)	herbivore	5.25	23.50	1	5	3	1	1	2
Coleoptera	Polyphaga	Chrysomelidae	*Longitarsus atricillus*	(Linnaeus, 1761)	herbivore	2.50	3.36	0	3	7	0	2	3
Coleoptera	Polyphaga	Chrysomelidae	*Longitarsus ganglbaueri*	Heikertinger, 1912	herbivore	2.00	1.87	0	1	0	0	1	0
Coleoptera	Polyphaga	Chrysomelidae	*Longitarsus jacobaeae*	(Waterhouse, 1858)	herbivore	3.00	5.42	1	1	1	1	1	1
Coleoptera	Polyphaga	Chrysomelidae	*Longitarsus kutscherae*	Rye, 1872	herbivore	2.00	1.87	1	0	0	1	0	0
Coleoptera	Polyphaga	Chrysomelidae	*Longitarsus luridus*	(Scopoli, 1763)	herbivore	1.85	1.53	19	16	11	9	11	8
Coleoptera	Polyphaga	Chrysomelidae	*Longitarsus melanocephalus*	(De Geer, 1775)	herbivore	2.50	3.36	7	11	13	6	7	7
Coleoptera	Polyphaga	Chrysomelidae	*Longitarsus nasturtii*	(Fabricius, 1792)	herbivore	1.75	1.32	0	0	11	0	0	3
Coleoptera	Polyphaga	Chrysomelidae	*Longitarsus noricus*	Leonardi, 1976	herbivore	2.10	2.13	0	1	1	0	1	1
Coleoptera	Polyphaga	Chrysomelidae	*Longitarsus pellucidus*	(Foudras, 1860)	herbivore	2.00	1.87	0	0	12	0	0	1
Coleoptera	Polyphaga	Chrysomelidae	*Longitarsus pratensis*	(Panzer, 1794)	herbivore	1.60	1.04	3	333	63	2	27	15
Coleoptera	Polyphaga	Chrysomelidae	*Longitarsus succineus*	(Foudras, 1860)	herbivore	1.95	1.75	13	5	19	8	3	5
Coleoptera	Polyphaga	Chrysomelidae	*Luperus flavipes*	(Linnaeus, 1761)	herbivore	4.40	14.80	0	1	0	0	1	0
Coleoptera	Polyphaga	Chrysomelidae	*Neogalerucella tenella*	(Linnaeus, 1761)	herbivore	4.00	11.53	7	0	0	1	0	0
Coleoptera	Polyphaga	Chrysomelidae	*Oulema duftschmidi*	(Redtenbacher, 1874)	herbivore	4.25	13.51	2	0	12	2	0	5
Coleoptera	Polyphaga	Chrysomelidae	*Oulema gallaeciana*	(Heyden, 1870)	herbivore	4.00	11.53	2	3	0	2	2	0
Coleoptera	Polyphaga	Chrysomelidae	*Oulema melanopus*	(Linnaeus, 1758)	herbivore	4.00	11.53	2	0	0	2	0	0
Coleoptera	Polyphaga	Chrysomelidae	*Phyllotreta atra*	(Fabricius, 1775)	herbivore	2.15	2.27	0	0	1	0	0	1
Coleoptera	Polyphaga	Chrysomelidae	*Phyllotreta cruciferae*	(Goeze, 1777)	herbivore	2.15	2.27	0	3	0	0	3	0
Coleoptera	Polyphaga	Chrysomelidae	*Phyllotreta nigripes*	(Fabricius, 1775)	herbivore	2.30	2.70	0	4	0	0	3	0
Coleoptera	Polyphaga	Chrysomelidae	*Phyllotreta undulata*	Kutschera, 1860	herbivore	2.15	2.27	1	0	0	1	0	0
Coleoptera	Polyphaga	Chrysomelidae	*Phyllotreta vittula*	(Redtenbacher, 1849)	herbivore	1.65	1.13	0	0	4	0	0	4
Coleoptera	Polyphaga	Chrysomelidae	*Psylliodes chrysocephalus*	(Linnaeus, 1758)	herbivore	3.00	5.42	0	0	23	0	0	10
Coleoptera	Polyphaga	Chrysomelidae	*Psylliodes picinus*	(Marsham, 1802)	herbivore	2.50	3.36	0	0	3	0	0	2
Coleoptera	Polyphaga	Chrysomelidae	*Sermylassa halensis*	(Linnaeus, 1767)	herbivore	6.00	33.35	1	8	0	1	2	0
Coleoptera	Polyphaga	Chrysomelidae	*Smaragdina salicina*	(Scopoli, 1763)	herbivore	5.50	26.55	0	1	0	0	1	0
Coleoptera	Polyphaga	Chrysomelidae	*Sphaeroderma rubidum*	(Graells, 1858)	herbivore	3.40	7.53	0	1	0	0	1	0
Coleoptera	Polyphaga	Chrysomelidae	*Sphaeroderma testaceum*	(Fabricius, 1775)	herbivore	3.00	5.42	0	0	1	0	0	1
Coleoptera	Polyphaga	Coccinellidae	*Coccinella quinquepunctata*	Linnaeus, 1758	predator	4.00	11.53	0	0	1	0	0	1
Coleoptera	Polyphaga	Coccinellidae	*Coccinella septempunctata*	Linnaeus, 1758	predator	6.60	42.81	1	13	62	1	10	23
Coleoptera	Polyphaga	Coccinellidae	*Coccinula quatuordecimpustulata*	(Linnaeus, 1758)	predator	3.50	8.12	0	0	3	0	0	3
Coleoptera	Polyphaga	Coccinellidae	*Harmonia axyridis*	(Pallas, 1773)	predator	7.00	49.94	1	0	1	1	0	1
Coleoptera	Polyphaga	Coccinellidae	*Hippodamia notata*	(Laicharting, 1781)	predator	5.00	20.68	1	0	0	1	0	0
Coleoptera	Polyphaga	Coccinellidae	*Hippodamia tredecimpunctata*	(Linnaeus, 1758)	predator	5.75	29.83	0	0	1	0	0	1
Coleoptera	Polyphaga	Coccinellidae	*Hippodamia variegata*	(Goeze, 1777)	predator	4.25	13.51	0	1	7	0	1	5
Coleoptera	Polyphaga	Coccinellidae	*Platynaspis luteorubra*	(Goeze, 1777)	predator	3.00	5.42	0	2	0	0	2	0
Coleoptera	Polyphaga	Coccinellidae	*Propylea quatuordecimpunctata*	(Linnaeus, 1758)	predator	4.00	11.53	2	1	11	1	1	8
Coleoptera	Polyphaga	Coccinellidae	*Rhyzobius litura*	(Fabricius, 1787)	predator	2.75	4.32	0	0	1	0	0	1
Coleoptera	Polyphaga	Coccinellidae	*Scymnus femoralis*	Gyllenhal, 1827	predator	2.50	3.36	0	1	0	0	1	0

List of species sampled in 2009

								Abundance			Number of plots		
Order	Suborder	Family	Genus/Species	Author/Year	Feeding guild	mean body length [mm]	estimated biomass [g]	Swabian Alb	Hainich-Dün	Schorfheide-Chorin	Swabian Alb	Hainich-Dün	Schorfheide-Chorin
Coleoptera	Polyphaga	Coccinellidae	*Scymnus frontalis*	(Fabricius, 1787)	predator	2.50	3.36	0	1	0	0	1	0
Coleoptera	Polyphaga	Coccinellidae	*Scymnus mimulus*	CapraFürsch, 1967	predator	2.65	3.92	1	0	0	1	0	0
Coleoptera	Polyphaga	Coccinellidae	*Tytthaspis sedecimpunctata*	(Linnaeus, 1761)	predator	2.75	4.32	0	19	60	0	6	20
Coleoptera	Polyphaga	Corylophidae	*Sericoderus lateralis*	(Gyllenhal, 1827)	predator	1.20	0.49	0	0	3	0	0	2
Coleoptera	Polyphaga	Curculionidae	*Anthonomus rubi*	(Herbst, 1795)	herbivore	2.75	4.32	1	2	1	1	2	1
Coleoptera	Polyphaga	Curculionidae	*Brachonyx pineti*	(Paykull, 1792)	herbivore	2.30	2.70	0	0	1	0	0	1
Coleoptera	Polyphaga	Curculionidae	*Ceutorhynchus floralis*	(Paykull, 1792)	herbivore	1.85	1.53	0	0	7	0	0	6
Coleoptera	Polyphaga	Curculionidae	*Ceutorhynchus obstrictus*	(Marsham, 1802)	herbivore	2.75	4.32	1	0	0	1	0	0
Coleoptera	Polyphaga	Curculionidae	*Datonychus angulosus*	(Boheman, 1845)	herbivore	3.00	5.42	0	0	6	0	0	2
Coleoptera	Polyphaga	Curculionidae	*Donus ovalis*	(Boheman, 1842)	herbivore	9.50	111.16	1	0	0	1	0	0
Coleoptera	Polyphaga	Curculionidae	*Donus tesselatus*	(Herbst, 1795)	herbivore	5.50	26.55	0	1	0	0	1	0
Coleoptera	Polyphaga	Curculionidae	*Eusomus ovulum*	Germar, 1824	herbivore	6.25	37.11	0	2	0	0	1	0
Coleoptera	Polyphaga	Curculionidae	*Glocianus punctiger*	(Gyllenhal, 1837)	herbivore	2.80	4.53	1	4	1	1	3	1
Coleoptera	Polyphaga	Curculionidae	*Grypus equiseti*	(Fabricius, 1775)	herbivore	5.40	25.30	0	0	1	0	0	1
Coleoptera	Polyphaga	Curculionidae	*Gymnetron labile*	(Herbst, 1795)	herbivore	1.95	1.75	1	3	0	1	1	0
Coleoptera	Polyphaga	Curculionidae	*Gymnetron pascuorum*	(Gyllenhal, 1813)	herbivore	1.80	1.42	0	17	10	0	6	3
Coleoptera	Polyphaga	Curculionidae	*Hadroplontus litura*	(Fabricius, 1775)	herbivore	3.35	7.24	0	0	1	0	0	1
Coleoptera	Polyphaga	Curculionidae	*Hypera nigrirostris*	(Fabricius, 1775)	herbivore	3.40	7.53	4	1	0	3	1	0
Coleoptera	Polyphaga	Curculionidae	*Hypera plantaginis*	(DeGeer, 1775)	herbivore	4.40	14.80	0	0	1	0	0	1
Coleoptera	Polyphaga	Curculionidae	*Hypera postica*	(Gyllenhal, 1813)	herbivore	4.65	17.10	0	1	0	0	1	0
Coleoptera	Polyphaga	Curculionidae	*Hypera suspiciosa*	(Herbst, 1795)	herbivore	5.20	22.92	2	2	0	2	2	0
Coleoptera	Polyphaga	Curculionidae	*Larinus turbinatus*	Gyllenhal, 1836	herbivore	6.50	41.13	0	0	3	0	0	3
Coleoptera	Polyphaga	Curculionidae	*Limnobaris talbum*	(Linnaeus 1758)	herbivore	3.65	9.07	0	0	5	0	0	2
Coleoptera	Polyphaga	Curculionidae	*Limobius borealis*	(Paykull, 1792)	herbivore	3.10	5.91	0	0	1	0	0	1
Coleoptera	Polyphaga	Curculionidae	*Liophloeus tessulatus*	(Muller O.F., 1776)	herbivore	9.00	96.48	0	0	1	0	0	1
Coleoptera	Polyphaga	Curculionidae	*Mecinus pyraster*	(Herbst, 1795)	herbivore	3.40	7.53	1	1	1	1	1	1
Coleoptera	Polyphaga	Curculionidae	*Micrelus ericae*	(Gyllenhal, 1813)	herbivore	1.95	1.75	0	0	1	0	0	1
Coleoptera	Polyphaga	Curculionidae	*Nedyus quadrimaculatus*	(Linnaeus, 1758)	herbivore	2.90	4.96	1	0	2	1	0	2
Coleoptera	Polyphaga	Curculionidae	*Otiorhynchus ovatus*	(Linnaeus, 1758)	herbivore	5.00	20.68	0	0	1	0	0	1
Coleoptera	Polyphaga	Curculionidae	*Phyllobius betulinus*	(Bechstein & Scharfenberg, 1805)	herbivore	5.00	20.68	5	4	0	4	3	0
Coleoptera	Polyphaga	Curculionidae	*Phyllobius oblongus*	(Linnaeus, 1758)	herbivore	4.50	15.69	1	0	0	1	0	0
Coleoptera	Polyphaga	Curculionidae	*Phyllobius pomaceus*	Gyllenhal, 1834	herbivore	8.50	83.06	0	0	1	0	0	1
Coleoptera	Polyphaga	Curculionidae	*Polydrusus impar*	Gozis, 1882	herbivore	6.60	42.81	0	1	0	0	1	0
Coleoptera	Polyphaga	Curculionidae	*Rhamphus subaeneus*	Illiger, 1807	herbivore	1.40	0.74	0	1	0	0	1	0
Coleoptera	Polyphaga	Curculionidae	*Rhinoncus pericarpius*	(Linnaeus, 1758)	herbivore	2.95	5.19	4	1	3	3	1	2
Coleoptera	Polyphaga	Curculionidae	*Rhynchaenus fagi*	(Linnaeus, 1758)	herbivore	2.60	3.73	87	3	0	24	3	0
Coleoptera	Polyphaga	Curculionidae	*Sibinia pyrrhodactyla*	Germar, 1824	herbivore	2.45	3.19	0	0	1	0	0	1
Coleoptera	Polyphaga	Curculionidae	*Sitona hispidulus*	(Fabricius, 1777)	herbivore	4.00	11.53	2	2	3	2	2	3
Coleoptera	Polyphaga	Curculionidae	*Sitona humeralis*	Stephens, 1831	herbivore	4.25	13.51	0	3	2	0	2	2
Coleoptera	Polyphaga	Curculionidae	*Sitona languidus*	Gyllenhal, 1834	herbivore	3.75	9.73	0	0	1	0	0	1
Coleoptera	Polyphaga	Curculionidae	*Sitona lepidus*	Gyllenhal, 1834	herbivore	5.25	23.50	11	4	8	9	4	8
Coleoptera	Polyphaga	Curculionidae	*Sitona lineatus*	(Linnaeus, 1758)	herbivore	4.00	11.53	3	0	0	2	0	0
Coleoptera	Polyphaga	Curculionidae	*Sitona sulcifrons*	(Thunberg, 1798)	herbivore	3.50	8.12	31	7	2	19	6	1
Coleoptera	Polyphaga	Curculionidae	*Sitona suturalis*	Stephens, 1831	herbivore	4.25	13.51	1	8	0	1	5	0
Coleoptera	Polyphaga	Curculionidae	*Tanysphyrus lemnae*	(Paykull, 1792)	herbivore	1.60	1.04	0	0	1	0	0	1
Coleoptera	Polyphaga	Curculionidae	*Trachyphloeus alternans*	Gyllenhal, 1834	herbivore	2.50	3.36	1	0	0	1	0	0
Coleoptera	Polyphaga	Curculionidae	*Trichosirocalus troglodytes*	(Fabricius, 1787)	herbivore	2.60	3.73	19	61	71	10	19	13
Coleoptera	Polyphaga	Curculionidae	*Tychius breviusculus*	Desbrochers, 1873	herbivore	2.35	2.86	0	0	1	0	0	1
Coleoptera	Polyphaga	Curculionidae	*Tychius picirostris*	(Fabricius, 1787)	herbivore	2.15	2.27	13	69	18	10	23	8
Coleoptera	Polyphaga	Curculionidae	*Tychius schneideri*	(Herbst, 1795)	herbivore	2.60	3.73	2	0	0	1	0	0
Coleoptera	Polyphaga	Curculionidae	*Tychius stephensi*	Schonherr, 1836	herbivore	2.25	2.55	5	6	0	5	2	0
Coleoptera	Polyphaga	Drilidae	*Drilus concolor*	Ahrens, 1812	predator	4.50	15.69	1	2	0	1	2	0
Coleoptera	Polyphaga	Elateridae	*Agriotes acuminatus*	(Stephens, 1830)	herbivore	7.00	49.94	0	1	0	0	1	0
Coleoptera	Polyphaga	Elateridae	*Agriotes lineatus*	(Linnaeus, 1767)	herbivore	10.00	127.15	0	2	3	0	2	2
Coleoptera	Polyphaga	Elateridae	*Agriotes obscurus*	(Linnaeus, 1758)	herbivore	8.75	89.61	5	1	0	5	1	0
Coleoptera	Polyphaga	Elateridae	*Agriotes sputator*	(Linnaeus, 1758)	herbivore	7.25	54.75	3	14	0	2	6	0
Coleoptera	Polyphaga	Elateridae	*Agrypnus murina*	(Linnaeus, 1758)	predator	14.50	336.58	13	4	1	8	4	1
Coleoptera	Polyphaga	Elateridae	*Athous bicolor*	(Goeze, 1777)	herbivore	9.50	111.16	0	12	0	0	7	0
Coleoptera	Polyphaga	Elateridae	*Athous haemorrhoidalis*	(Fabricius, 1801)	herbivore	12.25	216.38	1	0	7	1	0	1
Coleoptera	Polyphaga	Elateridae	*Athous subfuscus*	(Muller O.F., 1767)	predator	9.15	100.74	2	0	0	2	0	0

List of species sampled in 2009

								Abundance			Number of plots		
**Order**	**Suborder**	**Family**	**Genus/Species**	**Author/Year**	**Feeding guild**	**mean body length [mm]**	**estimated biomass [g]**	**Swabian Alb**	**Hainich-Dün**	**Schorfheide-Chorin**	**Swabian Alb**	**Hainich-Dün**	**Schorfheide-Chorin**
Coleoptera	Polyphaga	Elateridae	*Cidnopus pilosus*	(Leske, 1785)	herbivore	10.25	135.64	0	2	0	0	2	0
Coleoptera	Polyphaga	Elateridae	*Cidnopus quercus*	(Olivier, 1790)	herbivore	5.00	20.68	1	0	0	1	0	0
Coleoptera	Polyphaga	Elateridae	*Hemicrepidius hirtus*	(Herbst, 1784)	herbivore	14.50	336.58	1	2	0	1	1	0
Coleoptera	Polyphaga	Elateridae	*Hemicrepidius niger*	(Linnaeus, 1758)	herbivore	12.25	216.38	83	1	4	27	1	2
Coleoptera	Polyphaga	Elateridae	*Kibunea minutus*	(Linnaeus, 1758)	herbivore	6.75	45.40	2	2	0	2	1	0
Coleoptera	Polyphaga	Elateridae	*Oedostethus quadripustulatus*	(Fabricius, 1792)	herbivore	3.40	7.53	0	0	1	0	0	1
Coleoptera	Polyphaga	Hydrophilidae	*Helophorus brevipalpis*	Bedel, 1881	herbivore	2.70	4.12	0	1	0	0	1	0
Coleoptera	Polyphaga	Hydrophilidae	*Helophorus nubilus*	Fabricius, 1777	herbivore	3.50	8.12	0	0	1	0	0	1
Coleoptera	Polyphaga	Hydrophilidae	*Megasternum obscurum*	(Marsham, 1802)	herbivore	1.95	1.75	0	0	1	0	0	1
Coleoptera	Polyphaga	Kateritidae	*Brachypterus glaber*	(Stephens, 1832)	herbivore	2.10	2.13	0	0	3	0	0	3
Coleoptera	Polyphaga	Kateritidae	*Brachypterus urticae*	(Fabricius, 1792)	herbivore	1.90	1.64	0	1	0	0	1	0
Coleoptera	Polyphaga	Melyridae	*Dasytes aeratus*	Stephens, 1830	predator	4.00	11.53	0	1	0	0	1	0
Coleoptera	Polyphaga	Melyridae	*Dolichosoma lineare*	(Rossi, 1794)	predator	6.00	33.35	0	5	0	0	3	0
Coleoptera	Polyphaga	Mordellidae	*Mordellistena parvula*	(Gyllenhal, 1827)	herbivore	2.90	4.96	0	1	0	0	1	0
Coleoptera	Polyphaga	Mordellidae	*Mordellistena pumila*	(Gyllenhal, 1810)	herbivore	4.33	14.19	0	4	0	0	3	0
Coleoptera	Polyphaga	Mordellidae	*Variimorda villosa*	(Schrank, 1781)	herbivore	7.00	49.94	0	0	1	0	0	1
Coleoptera	Polyphaga	Nitidulidae	*Meligethes aeneus*	(Fabricius, 1775)	herbivore	2.10	2.13	20	18	804	14	11	29
Coleoptera	Polyphaga	Nitidulidae	*Meligethes carinulatus*	Förster, 1849	herbivore	1.80	1.42	0	2	0	0	2	0
Coleoptera	Polyphaga	Nitidulidae	*Meligethes difficilis*	(Heer, 1841)	herbivore	2.25	2.55	1	0	0	1	0	0
Coleoptera	Polyphaga	Nitidulidae	*Meligethes nigrescens*	Stephens, 1830	herbivore	2.10	2.13	0	1	0	0	1	0
Coleoptera	Polyphaga	Nitidulidae	*Meligethes symphyti*	(Heer, 1841)	herbivore	2.60	3.73	0	0	6	0	0	1
Coleoptera	Polyphaga	Omalisidae	*Omalisus fontisbellaquaei*	Geoffroy, 1785	predator	7.50	59.84	4	0	0	4	0	0
Coleoptera	Polyphaga	Phalacridae	*Olibrus aeneus*	(Fabricius, 1792)	herbivore	2.25	2.55	0	1	2	0	1	2
Coleoptera	Polyphaga	Phalacridae	*Olibrus bicolor*	(Fabricius, 1792)	herbivore	2.75	4.32	0	10	5	0	6	2
Coleoptera	Polyphaga	Phalacridae	*Olibrus bimaculatus*	Kuster, 1848	herbivore	2.55	3.54	1	17	0	1	8	0
Coleoptera	Polyphaga	Phalacridae	*Olibrus flavicornis*	(Sturm, 1807)	herbivore	2.65	3.92	1	9	13	1	6	5
Coleoptera	Polyphaga	Phalacridae	*Olibrus millefolii*	(Paykull, 1800)	herbivore	1.65	1.13	0	0	1	0	0	1
Coleoptera	Polyphaga	Pselaphidae	*Bryaxis puncticollis*	(Denny, 1825)	predator	1.30	0.61	0	0	1	0	0	1
Coleoptera	Polyphaga	Rhynchitidae	*Caenorhinus germanicus*	(Herbst, 1797)	herbivore	2.45	3.19	0	1	0	0	1	0
Coleoptera	Polyphaga	Scarabaeidae	*Phyllopertha horticola*	(Linnaeus, 1758)	herbivore	9.75	118.98	70	0	0	25	0	0
Coleoptera	Polyphaga	Staphylinidae	*Acrotona benicki*	(Allen, 1940)	predator	2.00	1.87	0	0	1	0	0	1
Coleoptera	Polyphaga	Staphylinidae	*Acrotona parvula*	(Mannerheim, 1831)	predator	2.15	2.27	0	0	2	0	0	2
Coleoptera	Polyphaga	Staphylinidae	*Aleochara bipustulata*	(Linnaeus, 1761)	predator	3.25	6.69	0	1	1	0	1	1
Coleoptera	Polyphaga	Staphylinidae	*Aleuonota gracilenta*	(Erichson , 1839)	predator	2.60	3.73	0	0	1	0	0	1
Coleoptera	Polyphaga	Staphylinidae	*Amischa analis*	(Gravenhorst, 1802)	predator	2.10	2.13	0	0	31	0	0	13
Coleoptera	Polyphaga	Staphylinidae	*Amischa nigrofusca*	(Stephens, 1832)	predator	2.00	1.87	0	1	1	0	1	1
Coleoptera	Polyphaga	Staphylinidae	*Anotylus tetracarinatus*	(Block, 1799)	predator	1.95	1.75	0	0	1	0	0	1
Coleoptera	Polyphaga	Staphylinidae	*Atheta atramentaria*	(Gyllenhal, 1810)	predator	3.05	5.66	0	1	0	0	1	0
Coleoptera	Polyphaga	Staphylinidae	*Atheta cauta*	(Erichson, 1837)	predator	2.10	2.13	0	0	1	0	0	1
Coleoptera	Polyphaga	Staphylinidae	*Atheta fungi*	(Gravenhorst, 1806)	predator	2.60	3.73	0	0	11	0	0	7
Coleoptera	Polyphaga	Staphylinidae	*Atheta inquinula*	(Gravenhorst, 1802)	predator	1.15	0.44	0	0	1	0	0	1
Coleoptera	Polyphaga	Staphylinidae	*Atheta liliputana*	(Brisout, 1860)	predator	1.75	1.32	0	0	2	0	0	1
Coleoptera	Polyphaga	Staphylinidae	*Atheta macrocera*	(Thomson, 1856)	predator	2.20	2.41	0	0	1	0	0	1
Coleoptera	Polyphaga	Staphylinidae	*Atheta nigra*	(Kraatz, 1856)	predator	2.00	1.87	0	0	2	0	0	2
Coleoptera	Polyphaga	Staphylinidae	*Atheta palustris*	(Kiesenwetter, 1844)	predator	2.45	3.19	0	0	7	0	0	2
Coleoptera	Polyphaga	Staphylinidae	*Autalia rivularis*	(Gravenhorst, 1802)	predator	1.90	1.64	0	0	1	0	0	1
Coleoptera	Polyphaga	Staphylinidae	*Carpelimus corticinus*	(Gravenhorst, 1806)	predator	1.50	0.88	0	0	1	0	0	1
Coleoptera	Polyphaga	Staphylinidae	*Cypha longicornis*	(Paykull, 1800)	predator	1.20	0.49	0	1	5	0	1	4
Coleoptera	Polyphaga	Staphylinidae	*Cypha pulicaria*	(Erichson, 1839)	predator	1.00	0.31	0	0	1	0	0	1
Coleoptera	Polyphaga	Staphylinidae	*Euaesthetus laeviusculus*	Mannerheim, 1844	predator	1.80	1.42	0	0	1	0	0	1
Coleoptera	Polyphaga	Staphylinidae	*Eusphalerum minutum*	(Fabricius, 1792)	herbivore	2.50	3.36	0	0	1	0	0	1
Coleoptera	Polyphaga	Staphylinidae	*Eusphalerum sorbi*	(Gyllenhal, 1810)	herbivore	1.85	1.53	1	1	0	1	1	0
Coleoptera	Polyphaga	Staphylinidae	*Oligota pusillima*	(Gravenhorst, 1806)	predator	1.15	0.44	0	0	4	0	0	4
Coleoptera	Polyphaga	Staphylinidae	*Oxypoda haemorrhoa*	(Mannerheim, 1830)	predator	2.35	2.86	0	0	5	0	0	4
Coleoptera	Polyphaga	Staphylinidae	*Oxypoda tarda*	Sharp, 1871	predator	2.50	3.36	0	0	4	0	0	4
Coleoptera	Polyphaga	Staphylinidae	*Philonthus cognatus*	Stephens, 1832	predator	6.50	41.13	0	0	1	0	0	1
Coleoptera	Polyphaga	Staphylinidae	*Philonthus varians*	(Paykull, 1789)	predator	4.75	18.08	1	0	0	1	0	0
Coleoptera	Polyphaga	Staphylinidae	*Platystethus cornutus*	(Gravenhorst, 1802)	predator	3.50	8.12	0	0	1	0	0	1
Coleoptera	Polyphaga	Staphylinidae	*Pycnota paradoxa*	(Mulant et Rey, 1861)	predator	2.25	2.55	0	3	0	0	3	0
Coleoptera	Polyphaga	Staphylinidae	*Quedius vexans*	Eppelsheim, 1881	predator	9.50	111.16	1	0	0	1	0	0

List of species sampled in 2009

								Abundance			Number of plots		
**Order**	**Suborder**	**Family**	**Genus/Species**	**Author/Year**	**Feeding guild**	**mean body length [mm]**	**estimated biomass [g]**	**Swabian Alb**	**Hainich-Dün**	**Schorfheide-Chorin**	**Swabian Alb**	**Hainich-Dün**	**Schorfheide-Chorin**
Coleoptera	Polyphaga	Staphylinidae	*Stenus brunnipes*	Stephens, 1833	predator	3.45	7.82	0	0	1	0	0	1
Coleoptera	Polyphaga	Staphylinidae	*Stenus cicindeloides*	(Schaller, 1783)	predator	5.25	23.50	0	0	1	0	0	1
Coleoptera	Polyphaga	Staphylinidae	*Stenus impressus*	Germar, 1824	predator	4.10	12.30	0	1	0	0	1	0
Coleoptera	Polyphaga	Staphylinidae	*Tachinus laticollis*	Gravenhorst, 1802	predator	3.50	8.12	0	0	1	0	0	1
Coleoptera	Polyphaga	Staphylinidae	*Tachyporus chrysomelinus*	(Linnaeus, 1758)	predator	3.75	9.73	0	2	0	0	1	0
Coleoptera	Polyphaga	Staphylinidae	*Tachyporus dispar*	(Paykull, 1789)	predator	3.00	5.42	0	0	1	0	0	1
Coleoptera	Polyphaga	Staphylinidae	*Tachyporus hypnorum*	(Fabricius, 1775)	predator	3.50	8.12	0	1	1	0	1	1
Coleoptera	Polyphaga	Staphylinidae	*Tachyporus nitidulus*	(Fabricius, 1781)	predator	2.50	3.36	0	1	0	0	1	0
Coleoptera	Polyphaga	Staphylinidae	*Tachyporus pusillus*	Gravenhorst, 1806	predator	2.50	3.36	0	0	4	0	0	3
Coleoptera	Polyphaga	Staphylinidae	*Tinotus morion*	(Gravenhorst, 1802)	predator	2.25	2.55	0	0	4	0	0	3
Hemiptera	Cicadomorpha	Aphrophoridae	*Aphrophora alni*	(Fallén, 1805)	herbivore	7.50	59.84	52	3	0	11	3	0
Hemiptera	Cicadomorpha	Aphrophoridae	*Lepyronia coleoptrata*	(Linné, 1758)	herbivore	6.75	45.40	82	3	0	10	1	0
Hemiptera	Cicadomorpha	Aphrophoridae	*Neophilaenus lineatus*	(Linné, 1758)	herbivore	5.70	29.15	2	0	0	1	0	0
Hemiptera	Cicadomorpha	Aphrophoridae	*Philaenus spumarius*	(Linné, 1758)	herbivore	6.10	34.82	250	54	10	42	20	6
Hemiptera	Cicadomorpha	Cicadellidae	*Adarrus multinotatus*	(Boheman, 1847)	herbivore	3.13	6.06	10	1	0	2	1	0
Hemiptera	Cicadomorpha	Cicadellidae	*Anaceratagallia ribauti*	(Ossiannilsson, 1938)	herbivore	3.05	5.66	0	0	3	0	0	3
Hemiptera	Cicadomorpha	Cicadellidae	*Anoscopus serratulae*	(Fabricius, 1775)	herbivore	3.85	10.43	0	0	4	0	0	3
Hemiptera	Cicadomorpha	Cicadellidae	*Aphrodes makarovi*	Zachvatkin, 1948	herbivore	6.55	41.96	0	0	6	0	0	4
Hemiptera	Cicadomorpha	Cicadellidae	*Arocephalus languidus*	(Flor, 1861)	herbivore	2.55	3.54	0	0	10	0	0	4
Hemiptera	Cicadomorpha	Cicadellidae	*Arthaldeus pascuellus*	(Fallén, 1826)	herbivore	3.50	8.12	315	362	562	32	39	40
Hemiptera	Cicadomorpha	Cicadellidae	*Balclutha punctata*	(Fabricius, 1775)	herbivore	3.85	10.43	0	2	1	0	2	1
Hemiptera	Cicadomorpha	Cicadellidae	*Chlorita paolii*	(Ossiannilsson, 1939)	herbivore	2.85	4.74	0	3	26	0	2	10
Hemiptera	Cicadomorpha	Cicadellidae	*Cicadella viridis*	(Linné, 1758)	herbivore	7.35	56.75	0	0	16	0	0	11
Hemiptera	Cicadomorpha	Cicadellidae	*Cicadula persimilis*	(Edwards, 1920)	herbivore	4.60	16.62	41	26	135	7	10	26
Hemiptera	Cicadomorpha	Cicadellidae	*Cicadula quadrinotata*	(Fabricius, 1794)	herbivore	4.55	16.15	0	0	661	0	0	23
Hemiptera	Cicadomorpha	Cicadellidae	*Deltocephalus pulicaris*	(Fallén, 1806)	herbivore	2.75	4.32	96	46	164	22	10	31
Hemiptera	Cicadomorpha	Cicadellidae	*Diplocolenus bohemani*	(Zetterstedt, 1838)	herbivore	4.40	14.80	1	0	0	1	0	0
Hemiptera	Cicadomorpha	Cicadellidae	*Doratura exilis*	Horváth, 1903	herbivore	3.25	6.69	13	0	0	4	0	0
Hemiptera	Cicadomorpha	Cicadellidae	*Doratura homophyla*	(Flor, 1861)	herbivore	4.00	11.53	0	0	7	0	0	4
Hemiptera	Cicadomorpha	Cicadellidae	*Elymana sulphurella*	(Zetterstedt, 1828)	herbivore	4.70	17.59	3	0	1	3	0	1
Hemiptera	Cicadomorpha	Cicadellidae	*Emelyanoviana mollicula*	(Boheman, 1845)	herbivore	3.40	7.53	2	0	3	2	0	3
Hemiptera	Cicadomorpha	Cicadellidae	*Empoasca pteridis*	(Dahlbom, 1850)	herbivore	3.30	6.96	0	3	8	0	3	7
Hemiptera	Cicadomorpha	Cicadellidae	*Errastunus ocellaris*	(Fallén, 1806)	herbivore	3.35	7.24	159	102	125	19	17	26
Hemiptera	Cicadomorpha	Cicadellidae	*Erzaleus metrius*	(Flor, 1861)	herbivore	4.10	12.30	0	0	2	0	0	2
Hemiptera	Cicadomorpha	Cicadellidae	*Eupelix cuspidata*	(Fabricius, 1775)	herbivore	7.00	49.94	1	0	0	1	0	0
Hemiptera	Cicadomorpha	Cicadellidae	*Eupteryx atropunctata*	(Goeze, 1778)	herbivore	3.60	8.75	3	1	15	2	1	6
Hemiptera	Cicadomorpha	Cicadellidae	*Eupteryx aurata*	(Linné, 1758)	herbivore	3.90	10.79	0	3	0	0	2	0
Hemiptera	Cicadomorpha	Cicadellidae	*Eupteryx notata*	Curtis, 1937	herbivore	2.40	3.02	1	5	2	1	5	2
Hemiptera	Cicadomorpha	Cicadellidae	*Eupteryx urticae*	(Fabricius, 1803)	herbivore	3.25	6.69	0	0	74	0	0	3
Hemiptera	Cicadomorpha	Cicadellidae	*Euscelis incisus*	(Kirschbaum, 1858)	herbivore	3.70	9.40	57	36	89	9	17	23
Hemiptera	Cicadomorpha	Cicadellidae	*Fagocyba cruenta*	(Herrich-Schäffer, 1838)	herbivore	3.65	9.07	0	2	0	0	2	0
Hemiptera	Cicadomorpha	Cicadellidae	*Forcipata citrinella*	(Zetterstedt, 1828)	herbivore	3.45	7.82	0	0	2	0	0	2
Hemiptera	Cicadomorpha	Cicadellidae	*Graphocraerus ventralis*	(Fallén, 1806)	herbivore	5.50	26.55	8	0	0	6	0	0
Hemiptera	Cicadomorpha	Cicadellidae	*Hardya tenuis*	(Germar, 1821)	herbivore	3.35	7.24	24	0	0	4	0	0
Hemiptera	Cicadomorpha	Cicadellidae	*Macrosteles cristatus*	(Ribaut, 1927)	herbivore	3.75	9.73	737	0	0	20	0	0
Hemiptera	Cicadomorpha	Cicadellidae	*Macrosteles laevis*	(Ribaut, 1927)	herbivore	3.60	8.75	2710	67	1850	47	21	47
Hemiptera	Cicadomorpha	Cicadellidae	*Macrosteles viridigriseus*	(Edwards, 1922)	herbivore	3.35	7.24	0	4	0	0	1	0
Hemiptera	Cicadomorpha	Cicadellidae	*Mocydia crocea*	(Herrich-Schaeffer, 1837)	herbivore	4.85	19.10	0	1	0	0	1	0
Hemiptera	Cicadomorpha	Cicadellidae	*Psammotettix alienus*	(Dahlbom, 1850)	herbivore	4.15	12.69	13	41	70	7	13	12
Hemiptera	Cicadomorpha	Cicadellidae	*Psammotettix cephalotes*	(Herrich-Schaeffer, 1834)	herbivore	3.20	6.42	103	0	0	8	0	0
Hemiptera	Cicadomorpha	Cicadellidae	*Psammotettix confinis*	(Dahlbom, 1850)	herbivore	3.60	8.75	19	43	736	8	16	41
Hemiptera	Cicadomorpha	Cicadellidae	*Psammotettix helvolus*	(Kirschbaum, 1868)	herbivore	3.20	6.42	118	94	25	14	18	7
Hemiptera	Cicadomorpha	Cicadellidae	*Psammotettix kolosvarensis*	(Matsumura, 1908)	herbivore	3.75	9.73	0	0	45	0	0	14
Hemiptera	Cicadomorpha	Cicadellidae	*Rhopalopyx vitripennis*	(Flor, 1861)	herbivore	3.80	10.08	0	21	0	0	3	0
Hemiptera	Cicadomorpha	Cicadellidae	*Streptanus aemulans*	(Kirschbaum, 1868)	herbivore	5.05	21.23	0	1	1	0	1	1
Hemiptera	Cicadomorpha	Cicadellidae	*Streptanus marginatus*	(Kirschbaum, 1858)	herbivore	3.80	10.08	1	0	0	1	0	0
Hemiptera	Cicadomorpha	Cicadellidae	*Turrutus socialis*	(Flor, 1861)	herbivore	3.35	7.24	128	5	0	8	1	0
Hemiptera	Cicadomorpha	Cicadellidae	*Verdanus abdominalis*	(Fabricius, 1803)	herbivore	4.15	12.69	14	4	0	4	4	0
Hemiptera	Cicadomorpha	Cicadellidae	*Zyginidia scutellaris*	(Herrich-Schaeffer, 1838)	herbivore	2.85	4.74	3	8	41	3	8	17
Hemiptera	Fulgoromorpha	Cixiidae	*Cixius nervosus*	(Linné, 1758)	herbivore	7.50	59.84	0	1	0	0	1	0

List of species sampled in 2009

Order	Suborder	Family	Genus/Species	Author/Year	Feeding guild	mean body length [mm]	estimated biomass [g]	Abundance			Number of plots		
								Swabian Alb	Hainich-Dün	Schorfheide-Chorin	Swabian Alb	Hainich-Dün	Schorfheide-Chorin
Hemiptera	Fulgoromorpha	Delphacidae	*Acanthodelphax spinosa*	(Fieber, 1866)	herbivore	2.00	1.87	2	0	0	1	0	0
Hemiptera	Fulgoromorpha	Delphacidae	*Anakelisia perspicillata*	(Boheman, 1845)	herbivore	2.25	2.55	2	0	0	1	0	0
Hemiptera	Fulgoromorpha	Delphacidae	*Asiraca clavicornis*	(Fabricius, 1794)	herbivore	4.30	13.93	0	1	0	0	1	0
Hemiptera	Fulgoromorpha	Delphacidae	*Conomelus anceps*	(Germar, 1821)	herbivore	2.80	4.53	0	0	1	0	0	1
Hemiptera	Fulgoromorpha	Delphacidae	*Delphacinus mesomelas*	(Boheman, 1850)	herbivore	3.00	5.42	2	0	0	2	0	0
Hemiptera	Fulgoromorpha	Delphacidae	*Dicranotropis divergens*	Kirschbaum, 1868	herbivore	2.85	4.74	7	1	0	4	1	0
Hemiptera	Fulgoromorpha	Delphacidae	*Dicranotropis hamata*	(Boheman, 1847)	herbivore	3.80	10.08	2	3	2	2	3	2
Hemiptera	Fulgoromorpha	Delphacidae	*Ditropsis flavipes*	(Signoret, 1865)	herbivore	2.35	2.86	9	1	0	6	1	0
Hemiptera	Fulgoromorpha	Delphacidae	*Eurysula lurida*	(Fieber, 1866)	herbivore	3.10	5.91	0	0	1	0	0	1
Hemiptera	Fulgoromorpha	Delphacidae	*Javesella dubia*	(Kirschbaum, 1868)	herbivore	3.25	6.69	0	0	14	0	0	4
Hemiptera	Fulgoromorpha	Delphacidae	*Javesella forcipata*	(Boheman, 1847)	herbivore	3.00	5.42	1	0	0	1	0	0
Hemiptera	Fulgoromorpha	Delphacidae	*Javesella obscurella*	(Boheman, 1847)	herbivore	3.10	5.91	0	0	5	0	0	2
Hemiptera	Fulgoromorpha	Delphacidae	*Javesella pellucida*	(Fabricius, 1794)	herbivore	3.55	8.43	78	14	116	26	11	33
Hemiptera	Fulgoromorpha	Delphacidae	*Kelisia monoceros*	Ribaut, 1934	herbivore	3.10	5.91	1	2	0	1	2	0
Hemiptera	Fulgoromorpha	Delphacidae	*Kosswigianella exigua*	(Boheman, 1847)	herbivore	2.50	3.36	4	0	0	2	0	0
Hemiptera	Fulgoromorpha	Delphacidae	*Laodelphax striatella*	(Fallén, 1826)	herbivore	3.30	6.96	15	1	53	8	1	26
Hemiptera	Fulgoromorpha	Delphacidae	*Megadelphax sordidula*	(Stål, 1853)	herbivore	4.15	12.69	31	15	7	10	6	5
Hemiptera	Fulgoromorpha	Delphacidae	*Muellerianella brevipennis*	(Boheman, 1847)	herbivore	3.40	7.53	0	0	1	0	0	1
Hemiptera	Fulgoromorpha	Delphacidae	*Muellerianella extrusa*	(Scott, 1871)	herbivore	3.15	6.16	0	0	2	0	0	2
Hemiptera	Fulgoromorpha	Delphacidae	*Paraliburnia adela*	(Flor, 1861)	herbivore	3.70	9.40	0	0	6	0	0	1
Hemiptera	Fulgoromorpha	Delphacidae	*Ribautodelphax albostriata*	(Fieber, 1866)	herbivore	3.35	7.24	13	0	16	6	0	1
Hemiptera	Fulgoromorpha	Delphacidae	*Ribautodelphax pungens*	(Ribaut, 1953)	herbivore	3.25	6.69	2	0	0	1	0	0
Hemiptera	Fulgoromorpha	Delphacidae	*Stenocranus fuscovittatus*	(Stål, 1858)	herbivore	5.35	24.69	0	0	1	0	0	1
Hemiptera	Fulgoromorpha	Delphacidae	*Stenocranus major*	(Kirschbaum, 1868)	herbivore	6.05	34.08	0	0	2	0	0	2
Hemiptera	Fulgoromorpha	Delphacidae	*Stenocranus minutus*	(Fabricius, 1787)	herbivore	5.15	22.35	0	1	7	0	1	3
Hemiptera	Fulgoromorpha	Delphacidae	*Stiroma affinis*	Fieber, 1866	herbivore	3.75	9.73	1	0	0	1	0	0
Hemiptera	Fulgoromorpha	Delphacidae	*Stiroma bicarinata*	(Herrich-Schäffer, 1835)	herbivore	4.00	11.53	6	0	0	1	0	0
Hemiptera	Fulgoromorpha	Tettigometridae	*Tettigometra leucophaea*	(Preyssler, 1792)	herbivore	4.55	16.15	2	0	0	1	0	0
Hemiptera	Heteroptera	Anthocoridae	*Anthocoris nemoralis*	(Fabricius, 1794)	predator	3.70	9.40	0	0	1	0	0	1
Hemiptera	Heteroptera	Anthocoridae	*Orius minutus*	(Linnaeus, 1758)	predator	2.30	2.70	0	0	2	0	0	1
Hemiptera	Heteroptera	Anthocoridae	*Orius niger*	(Wolff, 1811)	predator	2.00	1.87	0	2	26	0	1	6
Hemiptera	Heteroptera	Anthocoridae	*Temnostethus pusillus*	(Herrich-Schaeffer, 1835)	predator	2.80	4.53	0	0	1	0	0	1
Hemiptera	Heteroptera	Berytidae	*Berytinus clavipes*	(Fabricius, 1775)	herbivore	7.40	57.77	1	1	0	1	1	0
Hemiptera	Heteroptera	Berytidae	*Berytinus minor*	(Herrich-Schaeffer, 1835)	herbivore	6.00	33.35	0	0	1	0	0	1
Hemiptera	Heteroptera	Coreidae	*Coreus marginatus*	(Linnaeus, 1758)	herbivore	13.00	252.83	1	0	3	1	0	2
Hemiptera	Heteroptera	Coreidae	*Coriomeris denticulatus*	(Scopoli, 1763)	herbivore	8.70	88.28	0	1	0	0	1	0
Hemiptera	Heteroptera	Lygaeidae	*Chilacis typhae*	(Perris, 1857)	herbivore	4.20	13.10	0	0	1	0	0	1
Hemiptera	Heteroptera	Lygaeidae	*Cymus claviculus*	(Fallén, 1807)	herbivore	3.20	6.42	0	0	1	0	0	1
Hemiptera	Heteroptera	Lygaeidae	*Drymus sylvaticus*	(Fabricius, 1775)	herbivore	4.10	12.30	0	0	1	0	0	1
Hemiptera	Heteroptera	Lygaeidae	*Megalonotus chiragra*	(Fabricius, 1794)	herbivore	5.90	31.91	0	1	0	0	1	0
Hemiptera	Heteroptera	Lygaeidae	*Nysius cymoides*	(Spinola, 1837)	herbivore	3.60	8.75	1	0	0	1	0	0
Hemiptera	Heteroptera	Lygaeidae	*Nysius thymi*	(Wolff, 1804)	herbivore	4.00	11.53	0	0	1	0	0	1
Hemiptera	Heteroptera	Lygaeidae	*Peritrechus geniculatus*	(Hahn, 1832)	herbivore	5.40	25.30	2	6	0	2	4	0
Hemiptera	Heteroptera	Lygaeidae	*Scolopostethus thomsoni*	Reuter, 1875	herbivore	3.70	9.40	1	0	0	1	0	0
Hemiptera	Heteroptera	Lygaeidae	*Stygnocoris sabulosus*	(Schilling, 1829)	herbivore	2.80	4.53	1	0	0	1	0	0
Hemiptera	Heteroptera	Miridae	*Acetropis carinata*	(Herrich-Schaeffer, 1841)	herbivore	6.40	39.49	0	51	0	0	2	0
Hemiptera	Heteroptera	Miridae	*Adelphocoris lineolatus*	(Goeze, 1778)	herbivore	8.40	80.52	16	22	13	7	9	9
Hemiptera	Heteroptera	Miridae	*Adelphocoris seticornis*	(Fabricius, 1775)	herbivore	7.50	59.84	36	0	1	14	0	1
Hemiptera	Heteroptera	Miridae	*Amblytylus nasutus*	(Kirschbaum, 1856)	herbivore	4.30	13.93	17	272	50	6	12	10
Hemiptera	Heteroptera	Miridae	*Apolygus spinolae*	(Meyer-Dür, 1841)	herbivore	5.50	26.55	0	1	0	0	1	0
Hemiptera	Heteroptera	Miridae	*Calocoris roseomaculatus*	(De Geer, 1773)	herbivore	7.20	53.77	8	3	0	5	1	0
Hemiptera	Heteroptera	Miridae	*Capsus ater*	(Linnaeus, 1758)	herbivore	5.70	29.15	9	11	6	8	8	4
Hemiptera	Heteroptera	Miridae	*Charagochilus gyllenhalii*	(Fallén, 1807)	herbivore	3.80	10.08	0	1	1	0	1	1
Hemiptera	Heteroptera	Miridae	*Chlamydatus pulicarius*	(Fallén, 1807)	herbivore	2.50	3.36	0	2	3	0	1	1
Hemiptera	Heteroptera	Miridae	*Chlamydatus pullus*	(Reuter, 1870)	herbivore	2.30	2.70	5	0	38	1	0	8
Hemiptera	Heteroptera	Miridae	*Criocoris crassicornis*	(Hahn, 1834)	herbivore	3.30	6.96	1	0	0	1	0	0
Hemiptera	Heteroptera	Miridae	*Hadrodemus mflavum*	(Goeze, 1778)	herbivore	7.30	55.74	1	0	0	1	0	0
Hemiptera	Heteroptera	Miridae	*Halticus apterus*	(Linnaeus, 1758)	herbivore	2.60	3.73	0	1	1	0	1	1
Hemiptera	Heteroptera	Miridae	*Horistus orientalis*	(Gmelin, 1790)	herbivore	6.00	33.35	20	0	0	2	0	0
Hemiptera	Heteroptera	Miridae	*Leptopterna dolabrata*	(Linnaeus, 1758)	herbivore	8.30	78.03	1819	333	24	46	25	12

List of species sampled in 2009

								Abundance			Number of plots		
**Order**	**Suborder**	**Family**	**Genus/Species**	**Author/Year**	**Feeding guild**	**mean body length [mm]**	**estimated biomass [g]**	**Swabian Alb**	**Hainich-Dün**	**Schorfheide-Chorin**	**Swabian Alb**	**Hainich-Dün**	**Schorfheide-Chorin**
Hemiptera	Heteroptera	Miridae	*Leptopterna ferrugata*	(Fallén, 1807)	herbivore	7.80	66.31	265	304	0	12	13	0
Hemiptera	Heteroptera	Miridae	*Liocoris tripustulatus*	(Fabricius, 1781)	herbivore	4.40	14.80	0	0	3	0	0	2
Hemiptera	Heteroptera	Miridae	*Lygocoris pabulinus*	(Linnaeus, 1761)	herbivore	5.80	30.51	1	0	0	1	0	0
Hemiptera	Heteroptera	Miridae	*Lygus pratensis*	(Linnaeus, 1758)	herbivore	6.50	41.13	11	21	124	6	14	18
Hemiptera	Heteroptera	Miridae	*Lygus wagneri*	Remane, 1955	herbivore	6.20	36.34	1	0	0	1	0	0
Hemiptera	Heteroptera	Miridae	*Macrotylus paykullii*	(Fallén, 1807)	herbivore	3.20	6.42	1	0	0	1	0	0
Hemiptera	Heteroptera	Miridae	*Megaloceroea recticornis*	(Geoffroy, 1785)	herbivore	9.00	96.48	62	240	25	12	11	11
Hemiptera	Heteroptera	Miridae	*Megalocoleus molliculus*	(Fallén, 1807)	herbivore	4.50	15.69	0	3	0	0	2	0
Hemiptera	Heteroptera	Miridae	*Monalocoris filicis*	(Linnaeus, 1758)	herbivore	2.50	3.36	0	0	1	0	0	1
Hemiptera	Heteroptera	Miridae	*Notostira elongata*	(Geoffroy, 1785)	herbivore	7.30	55.74	34	95	226	13	30	39
Hemiptera	Heteroptera	Miridae	*Notostira erratica*	(Linnaeus, 1758)	herbivore	7.60	61.95	41	43	314	14	16	37
Hemiptera	Heteroptera	Miridae	*Orthocephalus coriaceus*	(Fabricius, 1777)	herbivore	4.80	18.58	2	0	0	1	0	0
Hemiptera	Heteroptera	Miridae	*Orthops basalis*	(A. Costa, 1853)	herbivore	4.60	16.62	0	36	1	0	7	1
Hemiptera	Heteroptera	Miridae	*Orthops kalmii*	(Linnaeus, 1758)	herbivore	4.40	14.80	0	1	1	0	1	1
Hemiptera	Heteroptera	Miridae	*Pinalitus rubricatus*	(Fallén, 1807)	herbivore	4.50	15.69	1	0	0	1	0	0
Hemiptera	Heteroptera	Miridae	*Pithanus maerkelii*	(Herrich-Schaeffer, 1838)	herbivore	4.50	15.69	0	13	0	0	1	0
Hemiptera	Heteroptera	Miridae	*Plagiognathus chrysanthemi*	(Wolff, 1804)	herbivore	3.60	8.75	53	6	66	15	4	18
Hemiptera	Heteroptera	Miridae	*Polymerus nigrita*	(Fallén, 1807)	herbivore	4.40	14.80	3	0	0	2	0	0
Hemiptera	Heteroptera	Miridae	*Polymerus palustris*	(Reuter, 1907)	herbivore	5.30	24.09	1	0	0	1	0	0
Hemiptera	Heteroptera	Miridae	*Polymerus unifasciatus*	(Fabricius, 1794)	herbivore	5.90	31.91	126	0	0	24	0	0
Hemiptera	Heteroptera	Miridae	*Stenodema calcarata*	(Fallén, 1807)	herbivore	7.30	55.74	3	47	301	2	12	16
Hemiptera	Heteroptera	Miridae	*Stenodema laevigata*	(Linnaeus, 1758)	herbivore	8.40	80.52	5	7	10	5	6	4
Hemiptera	Heteroptera	Miridae	*Stenotus binotatus*	(Fabricius, 1794)	herbivore	6.60	42.81	60	88	73	12	5	11
Hemiptera	Heteroptera	Miridae	*Trigonotylus caelestialium*	(Kirkaldy, 1902)	herbivore	5.90	31.91	534	38	290	42	15	43
Hemiptera	Heteroptera	Nabidae	*Nabis brevis*	Scholtz, 1847	predator	6.20	36.34	2	0	1	2	0	1
Hemiptera	Heteroptera	Nabidae	*Nabis ferus*	(Linnaeus, 1758)	predator	8.00	70.86	0	0	1	0	0	1
Hemiptera	Heteroptera	Nabidae	*Nabis flavomarginatus*	Scholtz, 1847	predator	8.60	85.64	3	0	0	3	0	0
Hemiptera	Heteroptera	Nabidae	*Nabis pseudoferus*	Remane, 1949	predator	7.50	59.84	2	4	21	2	3	13
Hemiptera	Heteroptera	Nabidae	*Nabis rugosus*	(Linnaeus, 1758)	predator	6.90	48.09	2	3	2	2	1	1
Hemiptera	Heteroptera	Pentatomidae	*Aelia acuminata*	(Linnaeus, 1758)	herbivore	8.50	83.06	2	6	2	1	3	2
Hemiptera	Heteroptera	Pentatomidae	*Carpocoris fuscispinus*	(Boheman, 1850)	herbivore	12.20	214.07	2	2	0	2	2	0
Hemiptera	Heteroptera	Pentatomidae	*Carpocoris purpureipennis*	(De Geer, 1773)	herbivore	12.00	205.00	1	2	1	1	2	1
Hemiptera	Heteroptera	Pentatomidae	*Dolycoris baccarum*	(Linnaeus, 1758)	herbivore	11.00	163.21	1	3	1	1	3	1
Hemiptera	Heteroptera	Pentatomidae	*Peribalus strictus*	(Fabricius, 1803)	herbivore	9.50	111.16	0	1	0	0	1	0
Hemiptera	Heteroptera	Pentatomidae	*Sciocoris cursitans*	(Fabricius, 1794)	herbivore	5.20	22.92	1	0	0	1	0	0
Hemiptera	Heteroptera	Piesmatidae	*Piesma maculatum*	(Laporte de Castelnau, 1833)	herbivore	2.70	4.12	0	0	1	0	0	1
Hemiptera	Heteroptera	Rhopalidae	*Brachycarenus tigrinus*	(Schilling, 1829)	herbivore	6.60	42.81	0	0	1	0	0	1
Hemiptera	Heteroptera	Rhopalidae	*Corizus hyoscyami*	(Linnaeus, 1758)	herbivore	9.40	108.12	0	2	0	0	2	0
Hemiptera	Heteroptera	Rhopalidae	*Liorhyssus hyalinus*	(Fabricius, 1794)	herbivore	6.50	41.13	11	0	0	7	0	0
Hemiptera	Heteroptera	Rhopalidae	*Myrmus miriformis*	(Fallén, 1807)	herbivore	7.70	64.11	0	0	3	0	0	2
Hemiptera	Heteroptera	Rhopalidae	*Rhopalus conspersus*	(Fieber, 1837)	herbivore	6.50	41.13	1	0	0	1	0	0
Hemiptera	Heteroptera	Rhopalidae	*Rhopalus parumpunctatus*	Schilling, 1829	herbivore	6.80	46.29	0	1	4	0	1	3
Hemiptera	Heteroptera	Rhopalidae	*Stictopleurus abutilon*	(Rossi, 1790)	herbivore	7.80	66.31	0	0	1	0	0	1
Hemiptera	Heteroptera	Rhopalidae	*Stictopleurus crassicornis*	(Linnaeus, 1758)	herbivore	7.40	57.77	2	0	0	2	0	0
Hemiptera	Heteroptera	Rhopalidae	*Stictopleurus punctatonervosus*	(Goeze, 1778)	herbivore	7.50	59.84	0	0	1	0	0	1
Hemiptera	Heteroptera	Scutelleridae	*Eurygaster maura*	(Linnaeus, 1758)	herbivore	9.70	117.39	0	9	0	0	5	0
Hemiptera	Heteroptera	Scutelleridae	*Eurygaster testudinaria*	(Geoffroy, 1785)	herbivore	9.20	102.19	2	0	3	1	0	3
Hemiptera	Heteroptera	Tingidae	*Catoplatus fabricii*	(Stål, 1868)	herbivore	4.10	12.30	1	0	0	1	0	0
Hemiptera	Heteroptera	Tingidae	*Derephysia foliacea*	(Fallén, 1807)	herbivore	3.30	6.96	0	0	1	0	0	1
Hemiptera	Heteroptera	Tingidae	*Kalama tricornis*	(Schrank, 1801)	herbivore	3.10	5.91	0	2	3	0	2	3
Hemiptera	Heteroptera	Tingidae	*Lasiacantha capucina*	(Germar, 1837)	herbivore	2.70	4.12	1	0	0	1	0	0
Hemiptera	Heteroptera	Tingidae	*Oncochila simplex*	(Herrich-Schaeffer, 1830)	herbivore	3.20	6.42	1	0	0	1	0	0
Hemiptera	Heteroptera	Tingidae	*Tingis cardui*	(Linnaeus, 1758)	herbivore	3.40	7.53	0	1	1	0	1	1
Orthoptera	Caelifera	Acrididae	*Chorthippus albomarginatus*	(De Geer, 1773)	herbivore	17.00	510.59	0	7	21	0	6	10
Orthoptera	Caelifera	Acrididae	*Chorthippus apricarius*	(Linnaeus, 1758)	herbivore	18.00	593.08	0	0	1	0	0	1
Orthoptera	Caelifera	Acrididae	*Chorthippus biguttulus*	(Linnaeus, 1758)	herbivore	18.00	593.08	13	10	15	9	9	8
Orthoptera	Caelifera	Acrididae	*Chorthippus dorsatus*	(Zetterstedt, 1821)	herbivore	20.00	781.62	0	3	46	0	3	19
Orthoptera	Caelifera	Acrididae	*Chorthippus montanus*	(Charpentier, 1825)	herbivore	18.00	593.08	0	0	9	0	0	5
Orthoptera	Caelifera	Acrididae	*Chorthippus parallelus*	(Zetterstedt, 1821)	herbivore	18.00	593.08	28	9	11	15	8	8
Orthoptera	Caelifera	Acrididae	*Euthystira brachyptera*	(Ocskay, 1826)	herbivore	20.00	781.62	10	0	0	3	0	0

List of species sampled in 2009

								Abundance			Number of plots		
Order	Suborder	Family	Genus/Species	Author/Year	Feeding guild	mean body length [mm]	estimated biomass [g]	Swabian Alb	Hainich-Dün	Schorfheide-Chorin	Swabian Alb	Hainich-Dün	Schorfheide-Chorin
Orthoptera	Caelifera	Acrididae	*Omocestus haemorrhoidalis*	(Charpentier, 1825)	herbivore	14.00	307.01	0	0	2	0	0	1
Orthoptera	Caelifera	Acrididae	*Omocestus viridulus*	(Linnaeus, 1758)	herbivore	18.00	593.08	2	0	0	2	0	0
Orthoptera	Caelifera	Acrididae	*Stenobothrus lineatus*	(Panzer, 1796)	herbivore	20.00	781.62	4	0	0	4	0	0
Orthoptera	Caelifera	Acrididae	*Stethophyma grossum*	(Linnaeus, 1758)	herbivore	26.00	1554.28	0	0	5	0	0	2
Orthoptera	Caelifera	Tetrigidae	*Tetrix bipunctata*	(Linnaeus, 1758)	herbivore	10.00	127.15	1	0	0	1	0	0
Orthoptera	Caelifera	Tetrigidae	*Tetrix subulata*	(Linnaeus, 1758)	herbivore	10.00	127.15	0	1	2	0	1	2
Orthoptera	Caelifera	Tetrigidae	*Tetrix tenuicornis*	(Sahlberg, 1893)	herbivore	9.00	96.48	1	0	0	1	0	0
Orthoptera	Ensifera	Meconematidae	*Meconema thalassinum*	(DeGeer, 1773)	predator	14.00	307.01	1	0	0	1	0	0
Orthoptera	Ensifera	Tettigoniidae	*Metrioptera roeselii*	(Hagenbach, 1822)	herbivore	16.00	435.61	0	1	2	0	1	2
